# Mechanism of substrate transport and inhibition of the human LAT1-4F2hc amino acid transporter

**DOI:** 10.1038/s41421-021-00247-4

**Published:** 2021-03-23

**Authors:** Renhong Yan, Yaning Li, Jennifer Müller, Yuanyuan Zhang, Simon Singer, Lu Xia, Xinyue Zhong, Jürg Gertsch, Karl-Heinz Altmann, Qiang Zhou

**Affiliations:** 1 Westlake Laboratory of Life Sciences and Biomedicine, Key Laboratory of Structural Biology of Zhejiang Province, School of Life Sciences, Westlake University, Hangzhou, Zhejiang 310024 China; 2 Institute of Biology, Westlake Institute for Advanced Study, Hangzhou, Zhejiang 310024 China; 3 Beijing Advanced Innovation Center for Structural Biology, Tsinghua-Peking Joint Center for Life Sciences, School of Life Sciences, Tsinghua University, Beijing 100085, China; 4 ETH Zürich, Department of Chemistry and Applied Biosciences, Institute of Pharmaceutical Sciences, Vladimir-Prelog-Weg 1- 5/10, 8093, Zurich, Switzerland; 5 Institute of Biochemistry and Molecular Medicine, University of Bern, Bühlstrasse 28 3012, Bern, Switzerland

**Keywords:** Cryoelectron microscopy, Mechanisms of disease

## Abstract

LAT1 (SLC7A5) is one of the representative light chain proteins of heteromeric amino acid transporters, forming a heterodimer with its heavy chain partner 4F2hc (SLC3A2). LAT1 is overexpressed in many types of tumors and mediates the transfer of drugs and hormones across the blood-brain barrier. Thus, LAT1 is considered as a drug target for cancer treatment and may be exploited for drug delivery into the brain. Here, we synthesized three potent inhibitors of human LAT1, which inhibit transport of leucine with IC_50_ values between 100 and 250 nM, and solved the cryo-EM structures of the corresponding LAT1-4F2hc complexes with these inhibitors bound at resolution of up to 2.7 or 2.8 Å. The protein assumes an outward-facing occluded conformation, with the inhibitors bound in the classical substrate binding pocket, but with their tails wedged between the substrate binding site and TM10 of LAT1. We also solved the complex structure of LAT1-4F2hc with 3,5-diiodo-l-tyrosine (Diiodo-Tyr) at 3.4 Å overall resolution, which revealed a different inhibition mechanism and might represent an intermediate conformation between the outward-facing occluded state mentioned above and the outward-open state. To our knowledge, this is the first time that the outward-facing conformation is revealed for the HAT family. Our results unveil more important insights into the working mechanisms of HATs and provide a structural basis for future drug design.

## Introduction

L-type amino acid transporter 1 (LAT1, also known as SLC7A5) mediates the pH-independent and sodium-independent exchanging transport of the large neutral amino acids, thyroid hormones, and pharmaceutical drugs across the plasma membrane^1–3^. As a light chain protein, LAT1 is covalently linked with the heavy chain protein 4F2hc (also known as SLC3A2) through a conserved disulfide bond, forming a heterodimeric LAT1-4F2hc complex that belongs to the heteromeric amino acid transporters (HAT) family. 4F2hc is essential for plasma membrane localization, stability, and the transport activity of LAT1^4^. LAT1 is highly expressed in the placenta^3,5,6^ and at the blood-brain barrier (BBB)^7^, mediating the transport of drugs and hormones across the BBB^8^. LAT1 is also overexpressed in many types of tumor cells^6^, continuously supplying amino acids for tumor cell growth and stimulating the activity of the mechanistic target of rapamycin (mTOR) by transporting leucine into cells^9–11^. Therefore, LAT1 is a potential drug target in cancer but it may also be exploited for drug delivery into the brain. The inward-open conformation cryo-EM structures of the human LAT1-4F2hc complex in its apo state or with the non-specific inhibitor 2-amino-2-norbornanecarboxylic acid (BCH) bound and some eukaryotic homologs of the HAT family were solved^12–15^. However, structures of LAT1 or other HAT family members in other conformations with or without inhibitors bound are not available yet. The atomic structures of prokaryotic homologs were commonly used to generate the homology models of LAT1^16–24^, due to the lack of an experimental structure with LAT1 in an outward-facing conformation.

There are more than 100 inhibitors of LAT1 reported^8^, most of which are amino acid derivatives. However, only few of them are potent and selective, including the most advanced compound JPH203, a triiodothyronine (T3)-derived amino acid, which is currently being evaluated in a Phase II clinical trial (UMIN000034080) in patients with advanced biliary tract cancers^25,26^. Apart from JPH203 and JPH203-related structures, other amino acid-based LAT1 inhibitors were also reported, including, for example, substrate tyrosine derivatives such as Diiodo-Tyr^23^, certain *meta*-substituted phenylalanine derivatives^27^, and KMH-233^28^. In addition to amino acid-based inhibitors, other modified amino acids have also been reported to be improved LAT-1 substrates, such as certain conformationally restricted phenylalanine derivatives^29^. In particular, the alkylating agent DL-2-amino-7-bis[(2-chloroethyl)amino]-l,2,3,4-tetrahydro-2-naphthoic acid has been shown to be a potent competitive inhibitor of BCH transport in murine L1210 leukemic cells, and to possess enhanced in vitro antitumor activity and reduced myelo-suppressive activity when compared to the clinically approved drug melphalan^30^.

In this work, we designed and synthesized three bicyclic *meta*-tyrosine-based LAT1 inhibitors, designated JX-075, JX-078, and JX-119. We next solved the cryo-EM structures of the human LAT1-4F2hc complex bound with each of these three inhibitors at a resolution of 2.7 Å. These inhibitor-bound structures adopt an outward-occluded conformation and have undergone significant conformational changes compared with the inward-facing conformation, while the interaction interfaces between LAT1 and 4F2hc remain largely conserved. The amino acid moiety of the inhibitors is bound to the classical binding site in unwound regions of the transmembrane helix (TM) 1 and 6 (TM1 and TM6), while the hydrophobic tail moiety is stuck between the substrate binding site and TM10, thus preventing the rotation of TM10 of LAT1 during the transport cycle. Additionally, we also solved the structure of the LAT1-4F2hc complex bound with Diiodo-Tyr. Structural comparison suggested that this structure might represent an intermediate conformation between the outward-facing occluded and outward-open states. In summary, these results provide important structural insights into the working mechanism of HATs and clues for drug design.

## Results

### Synthesis and biochemical characterizations of LAT1 inhibitors

**Fig. 1:**
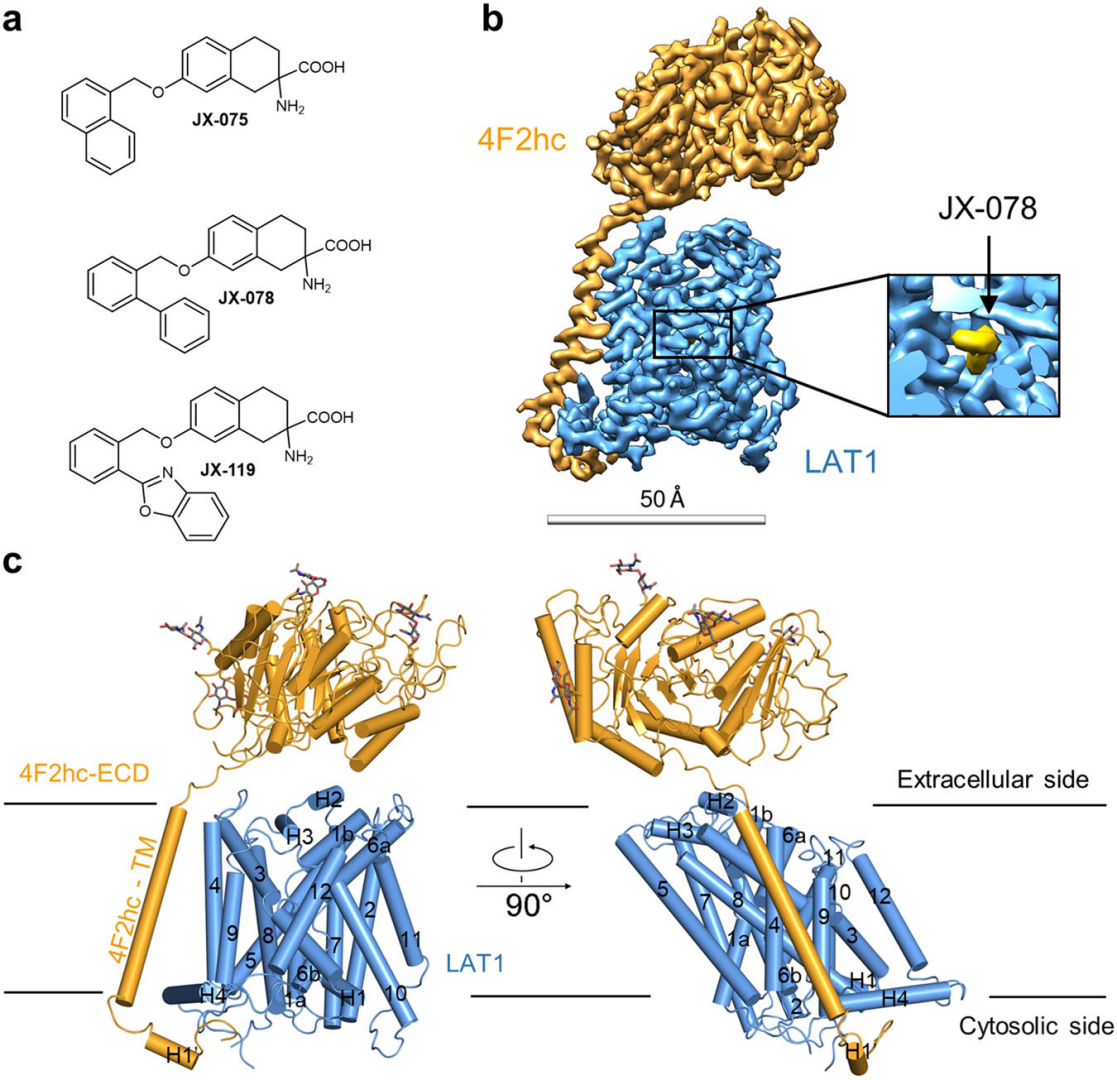
Overall structure of the LAT1-4F2hc bound with JX-078. **a** Chemical formulas of the designed LAT1 inhibitors. **b** The overall cryo-EM map of the LAT1-4F2hc complex bound with JX-078. **c** The overall structure of the LAT1-4F2hc complex bound with JX-078. The glycosylation moieties are shown as sticks. H, helix. 4F2hc and LAT1 are colored orange and deep blue, respectively.

Merging previous literature findings with our own recent observation of the moderate LAT1 inhibitory activity of O-benzyl-l-*meta*-tryrosine with the half maximal inhibitory concentration (IC_50_) of 15 μM (Müller, J., Rubin, M., Gertsch, J., Altmann, K.-H., unpublished data) eventually led to the design and synthesis of the three bicyclic *meta*-tyrosine derivatives JX-075, JX-078, and JX-119 (Fig. 1a). For the details of the synthesis of these compounds, please see the supplementary information. The constrained L-*meta*-tyrosine derivatives JX-075, JX-078, and JX-119 all inhibit transport of [^3^H]-L-leucine into HT-29 cells, which are known to express high levels of LAT1, with IC_50_ values between 100 and 250 nM (Table 1 and Supplementary Fig. S1). In addition, JX-075, JX-078, and JX-119 did not induce measurable leucine efflux at a concentration of up to 100 μM, indicating that none of these compounds is a substrate for LAT1 (Supplementary Fig. S2).

**Table 1:** Inhibition of [^3^H]-l-leucine transport into HT-29 cells by JX-075, JX-078, and JX-119.

Compound	IC_50_ [nM]	95% Confidence interval [nM]
**JX-075**	165	151–180
**JX-078**	121	108–137
**JX-119**	234	87–293

### Structural determination of LAT1-4F2hc complex with inhibitors bound

To investigate the interactions of LAT1 with the above inhibitors, we solved the cryo-EM structures of the human LAT1-4F2hc complex bound with each compound at resolution of up to 2.7 or 2.8 Å (Supplementary Figs. S3–S6). The details of sample preparation, data collection and processing, and model building can be found in the “Materials and methods” section. While the global structures of the LAT1-4F2hc complexes with inhibitors bound are similar to each other and also similar to the previously solved structures of the LAT1-4F2hc complex, as an important difference, LAT1 now exhibits an outward-occluded conformation (Fig. 1b and Supplementary Figs. S7 and S8). For reasons of clarity, only the structure of LAT1 with JX-078 bound is used for structural analysis in the following discussion (Fig. 1b, c).

### JX-078 bound LAT1

**Fig. 2:**
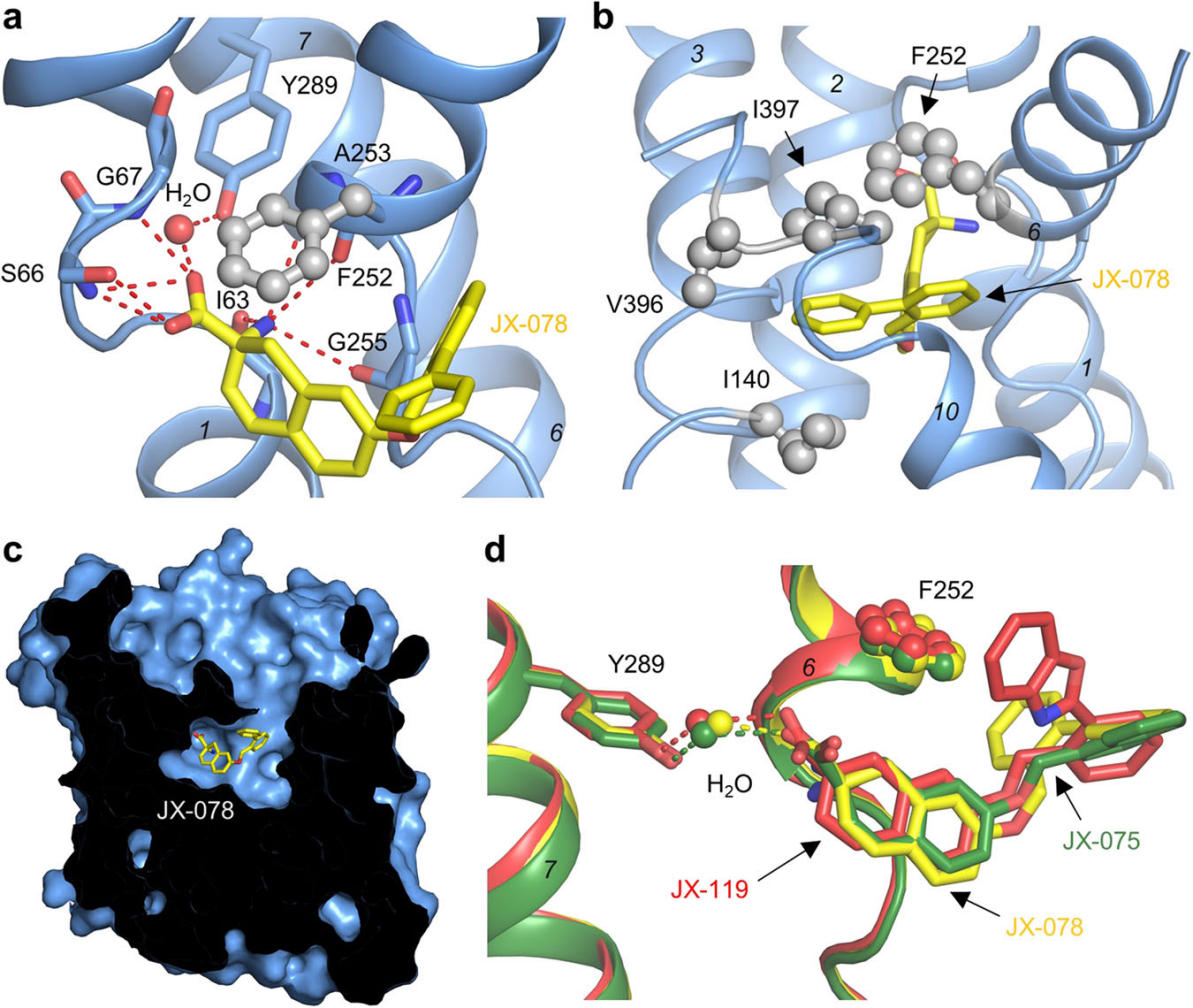
Inhibitor binding analysis. **a** JX-078 binding mode in LAT1. **b** The hydrophobic interactions around the tail of JX-078. **c** LAT1 has a broad extracellular vestibule above JX-078. **d** Comparison of the binding mode of the three inhibitors. JX-075, JX-078, and JX-119 are colored green, yellow, and red, respectively.

The inhibitor JX-078 is bound in the center of LAT1 in a similar way as BCH (Supplementary Fig. S9), with the carboxyl oxygen atoms and the ammonium group being hydrogen bonded with main chain atoms in the unwound regions of TM1 and TM6, respectively. The carboxyl oxygen atom of JX-078 is also hydrogen bonded with the hydroxyl oxygen of Tyr289 on TM7 through a water molecule, similar to the bacterial homolog^31^ (Fig. 2a). The hydrophobic tail of JX-078 is surrounded by the side chains of the conserved hydrophobic residues Ile140 on TM3, Phe252 on TM6, and Val396 and Ile397 on TM10, pushing away TM10 and partially disrupting the secondary structure of TM3 and TM10. This results in the outward-occluded conformation by locking TM10 in place; the latter is believed to rotate during the transition between the inward-facing and the outward-facing conformation (Fig. 2b, c and Supplementary Figs. S9 and S10). The side chain of the gating residue Phe252 on TM6 sits above the bound inhibitor, thereby blocking the putative transport path. JX-075 and JX-119 displayed a similar binding pattern (Fig. 2d).

### LAT1 with Diiodo-Tyr bound

**Fig. 3:**
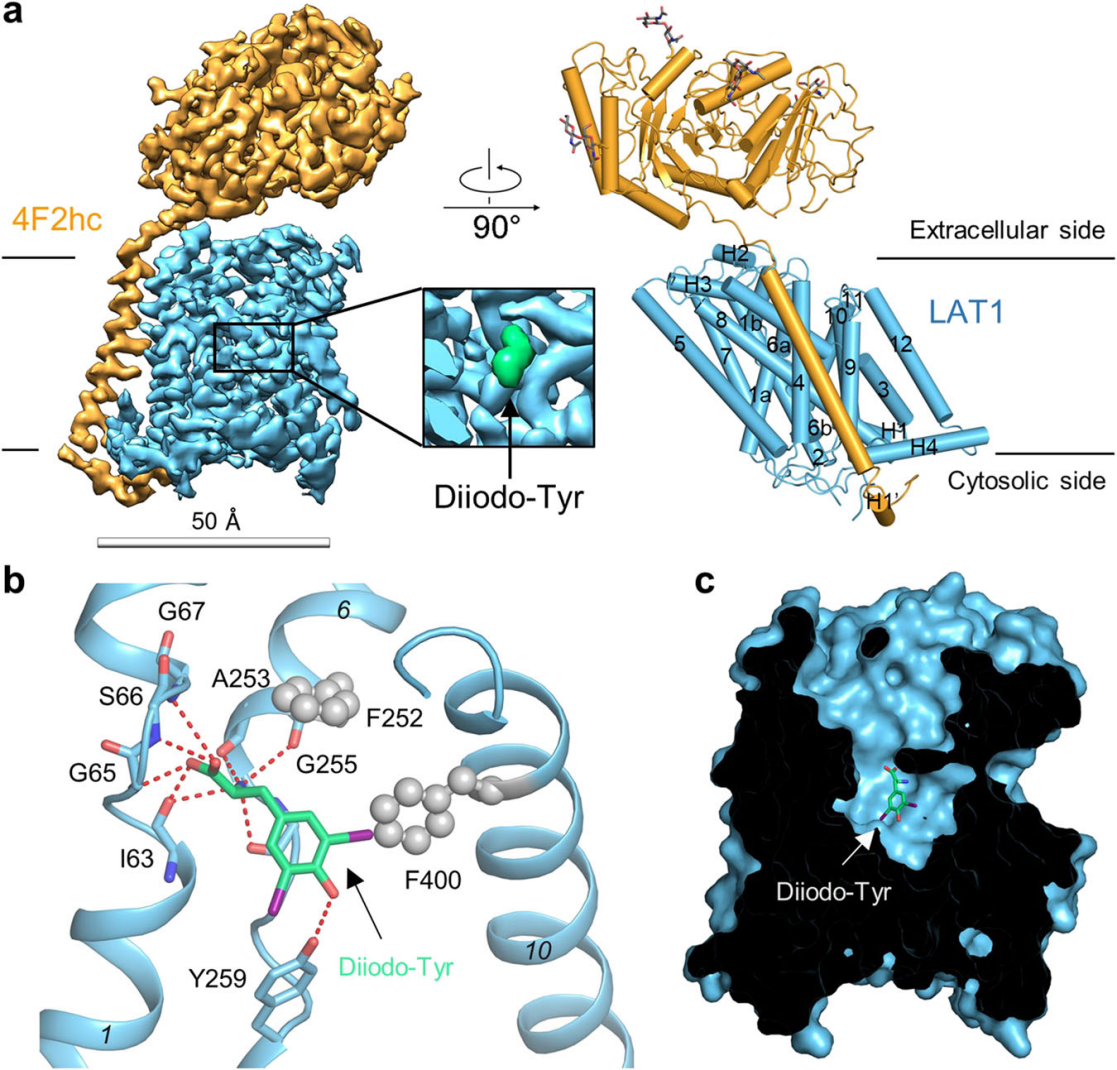
Overall structure of the LAT1-4F2hc bound with Diiodo-Tyr. **a** The overall structure of the LAT1-4F2hc bound with Diiodo-Tyr. The structure on the left is the EM map of the complex. The structure on the right is the overall structure of the complex. The glycosylation moieties are shown as sticks. H, helix. 4F2hc and LAT1 are colored orange and blue, respectively. **b** Diiodo-Tyr binding mode in LAT1. **c** LAT1 has a broad extracellular vestibule above Diiodo-Tyr.

To further investigate the inhibition mechanism of LAT1, we chose Diiodo-Tyr, which has been reported to inhibit LAT1 with an IC_50_ of 7.9 μM, as a promising candidate^23^. The cryo-EM structure of the Diiodo-Tyr bound LAT1-4F2hc complex was solved at 3.4 Å overall resolution, which also exhibits an outward-facing occluded conformation with a slight but distinct difference in the inhibitor binding pattern (Fig. 3a). Again, the α-carboxylate group and the protonated α-amino group of the inhibitor form hydrogen bonds with main chain atoms in the unwound regions of TM1 and TM6 of LAT1, respectively. The phenolic hydroxyl group is hydrogen bonded with the side chain of Tyr259 on TM6. The side chain of Diiodo-Tyr interacts with Phe252 on TM6 and Phe400 on TM10 (Fig. 3b, c) without disrupting the secondary structure of TM10. Diiodo-Tyr might lock LAT1 in an outward-facing conformation due to steric constraints imposed by the bulky iodine substituents. The Tyr259 and Phe400 of LAT1 are not very conserved among light chains of HAT, suggesting the binding site for Diiodo-Tyr can be used to develop homolog-specific inhibitors (Supplementary Fig. S11).

### Outward-occluded conformation of LAT1

**Fig. 4:**
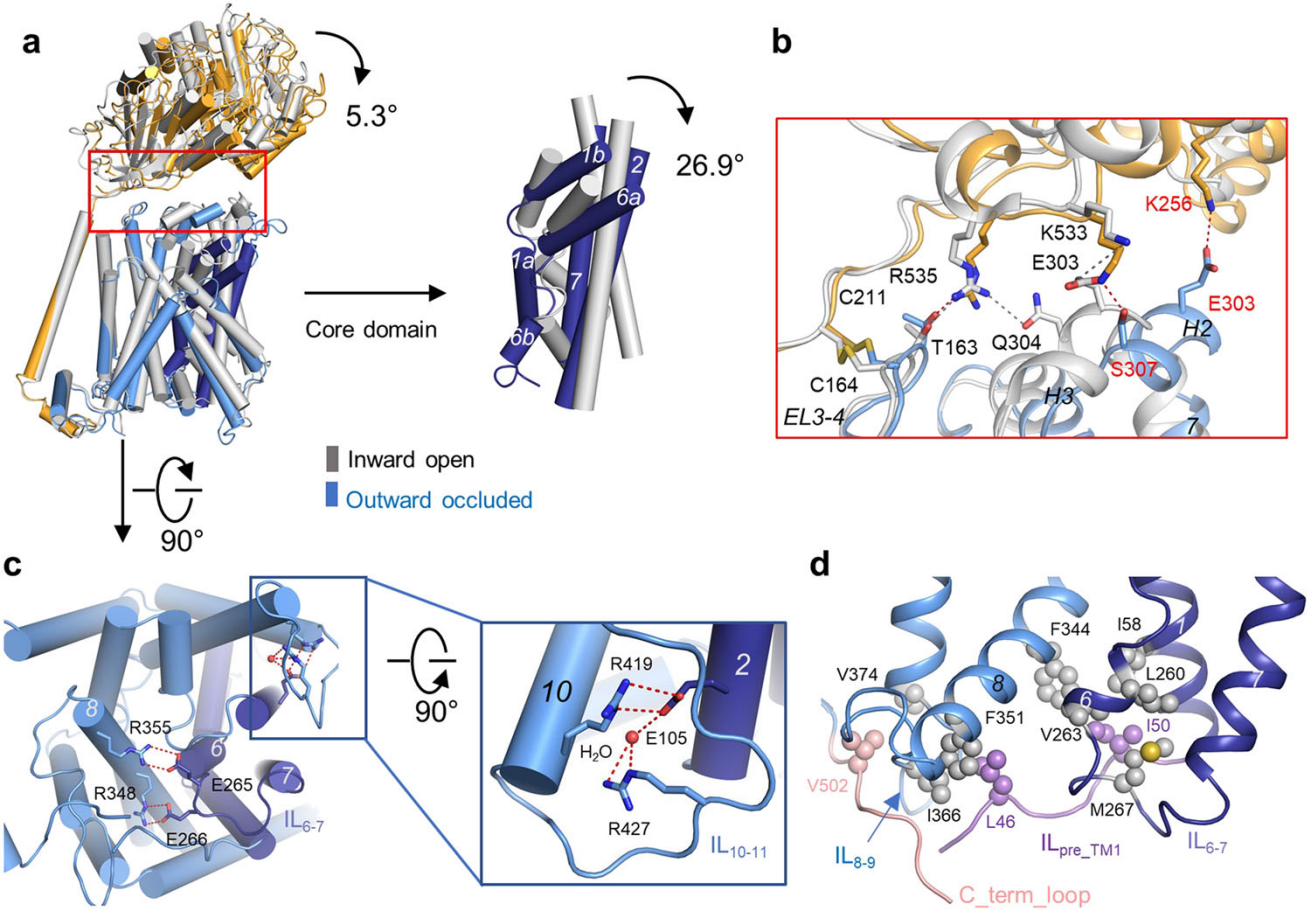
Conformational change between inward-open and outward-occluded state. **a** Comparison of the outward-occluded structure of LAT1 and the inward-facing structure of LAT1 (PDB ID: 6IRT). The core domain of the outward-occluded LAT1 rotates for ~27° compared with that of inward-open LAT1. The interaction mode at the extracellular interface of the LAT1-4F2hc complex is different between the inward-facing and the outward-occluded conformations. The red box corresponds to **b. b** The details of the extracellular interface and the polar interactions are shown as red dashed lines for the outward-occluded conformation and gray dashed lines for the inward-facing conformation. The new residues in the outward-occluded conformation are labeled in red. **c** The hydrogen bond network at the intracellular side of the outward-facing conformation. **d** The hydrophobic interactions stabilize the outward-facing conformation with the hydrophobic residues shown as spheres.

The outward-occluded structure of the LAT1-4F2hc complex with JX-078 bound shows dramatic changes compared to the inward-open conformation (Fig. 4a, Supplementary Fig. S10a, and Movie S1). The extracellular domain (ECD) of 4F2hc rotates by about 5.3° compared with the inward-open conformation. The interfaces between LAT1 and 4F2hc are partially conserved in the outward-occluded conformation, including the intracellular interface and membrane region interface, whereas the extracellular interface exhibits an obvious shift (Fig. 4b). Lys533 of 4F2hc and Glu303 of LAT1, a residue pair interacting with each other in the inward-facing conformation, are now hydrogen bonded with Ser307 of LAT1 and Lys256 of 4F2hc in the JX-078 bound structure, respectively. Arg535 of 4F2hc is hydrogen bonded only with Thr163 of LAT1, no longer interacting with Gln304 of LAT1. The extracellular interface is conserved between the JX-078 bound and the Diiodo-Tyr bound structures (Supplementary Fig. S12a), which also shows dramatic changes compared with the inward-open conformation (Supplementary Fig. S10b and Movie S2). The different interaction patterns between LAT1 and 4F2hc stabilize different conformations of the light chain LAT1, supporting our previous hypothesis that 4F2hc might stabilize the scaffolding domain of LAT1 and facilitate substrate transport^13^.

In the JX-078 bound outward-occluded conformation, the core domain of LAT1 formed by TMs 1, 2, 6, and 7 rotates by about 27°, compared with the inward-open conformation in a rocking bundle mode^32,33^, closing the intracellular vestibule and opening the extracellular vestibule (Fig. 4c). At the intracellular side of the outward-facing conformation, residues Arg348 and Arg355 on TM 8 are hydrogen bonded with residues Glu266 and Glu265 on the intracellular linker between TM6 and TM7 (IL_6–7_), respectively. Arg419 on TM10 and Arg427 on the intracellular linker between TM10 and TM11 (IL_10–11_) are hydrogen bonded with Glu105 on TM2 directly or via a water molecule, respectively (Fig. 4c). In addition to the hydrophilic interactions, the outward-facing conformation of LAT1 is also stabilized by hydrophobic interactions at the intracellular side, which involve Leu46 and Ile50 on the loop preceding TM1 (IL_pre_TM1_), Ile58 on TM1, Leu260 and Val263 on TM6, Met267 on IL_6–7_, Phe344, and Phe351 on TM8, Ile366, and Val374 on the intracellular linker between TM8 and TM9 (IL_8–9_), and Val502 on the C terminal loop (C_term_loop). The IL_pre_TM1_, which is near to C_term_loop, is only visible in the current outward-facing conformation (Fig. 4d) and invisible in the inward-facing conformation. Almost all of the above residues are conserved or conservatively substituted among all of the light chains of HAT (Supplementary Fig. S11), suggesting that the interactions described here also stabilize the outward-facing conformation of the other light chain members.

### The asymmetric movement of TM1 and TM6 of LAT1

**Fig. 5:**
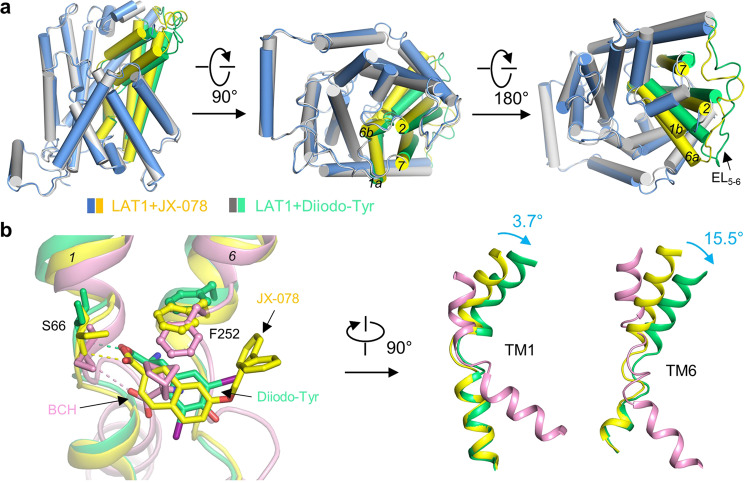
The asymmetrical movement of TM1 and TM6. **a** Comparison between the outward-occluded structure of LAT1 bound with JX-078 and that bound with Diiodo-Tyr, with the side view, the bottom view, and the top view shown in left, middle, and right panel, respectively. **b** Structural comparison among the inward-facing structure of LAT1 bound with BCH and the outward-occluded structure of LAT1 bound with JX-078 or Diiodo-Tyr (left panel). The movement of TM1 and TM6 is shown in the middle and right panel, respectively. BCH, JX-078, and Diiodo-Tyr are colored pink, yellow, and lemon green, respectively.

The Diiodo-Tyr bound LAT1-4F2hc complex exhibits an outward-occluded conformation similar to the JX-078 bound complex. However, clear conformational differences are visible between the two structures. Compared to the JX-078 bound structure, the ECD of 4F2hc rotates by 2.2° in the Diiodo-Tyr bound structure, whereas there is an about 6° rotation around the intracellular end of the core domain, when the hash domains (TMs 3, 4, 8, and 9) are superimposed (Fig. 5a, Supplementary Figs. S10c, S12b, and Movie S3). Further detailed structural comparison between the core domains indicates that TM6a and TM1b in the Diiodo-Tyr bound structure undergo a 15.5° and 3.7° rotation with respect to the JX-078 bound structure, respectively, whereas the other parts can be superimposed very well (Fig. 5b). The side chain of the gating residue Phe252 in the Diiodo-Tyr bound structure, though keeping the occluded conformation, is further pushed away from the substrate binding site with respect to that in the JX-078 bound structure (Fig. 5b).

## Discussion

LAT1 is overexpressed in many cancer cells and regarded as a promising anticancer drug target as the inhibition of LAT1 significantly decreases the viability of cancer cells. In this work, we synthesized three potent LAT1 inhibitors, JX-075, JX-078, and JX-119, and solved the complex structures of these inhibitors with the LAT1-4F2hc complex using cryo-EM. We also solved the cryo-EM structure of the LAT1-4F2hc complex bound with Diiodo-Tyr. All of these complex structures exhibit an outward-occluded conformation for LAT1. For JX-075, JX-078, and JX-119, their hydrophobic tails are stuck between the substrate binding site and TM10 of LAT1, thus blocking the rotation of TM10. In Diiodo-Tyr, the two *meta* hydrogen atoms of tyrosine are replaced by bigger iodine atoms, generating a steric effect between the inhibitor tail and TM6 that locks LAT1 in an outward-occluded state. Therefore, these structures might represent two different inhibitory mechanisms, providing important clues for future drug design.

Another central question for transporters is how the inhibitors get in the current binding site. As the bacterial homologs of the eukaryotic transporters are suggested to be in equilibrium between the outward-facing and the inward-facing conformations^33^, LAT1 may also exist in such an equilibrium. The binding of the inhibitor breaks the equilibrium by selectively locking the outward-facing conformation. The other possibility is that the inhibitors enter into the central binding site through the intracellular vestibule. The binding of the inhibitors induces the transition from the inward-facing conformation to the outward-facing conformation. Then the long tail of the inhibitor prevents the transition from the outward-occluded conformation to the outward-open conformation as it is stuck between the substrate binding site and either TM10 (for JX inhibitors), which requires a longer length of the tail, or TM6 (for Diiodo-Tyr) which does not require a tail length as long as the former. This might explain why the Diiodo-Tyr bound structure exhibits a more open conformation than the JX-bound structures.

**Fig. 6:**
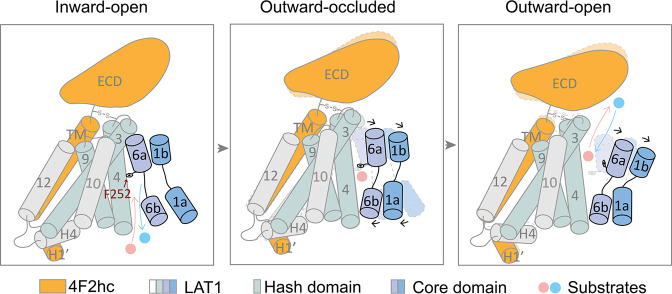
Putative working model for the LAT1-4F2hc complex. The model shows a transport mechanism of LAT1. LAT1 loads substrates in cytoplasm and then TM1a and TM6b rotate to the hash domain to close the inward gate. During the transition from the inward-facing conformation to the outward-facing conformation, TM1a and TM6b continue to rotate and TM1b and TM6a start to rotate away from the hash domain. Then TM1b and TM6a undergo further rotation and push the gating residue Phe252 away from the occluded configuration, triggering substrate release. For simplicity, TM2 and TM7 of the core domain and TM8 of the hash domain are not shown here. ECD, extracellular domain. H, helix. TM, transmembrane helix.

The transporters pass through different conformations to transport substrates across the cell membrane according to the well-established alternating access model. However, the concrete steps for the substrate loading and release are unclear in HAT transporters. In the Diiodo-Tyr bound structure the gating residue Phe252 stays in an occluded state, while TM6a undergoes a further opening rotation with respect to that in the JX bound structures. The former structure might thus represent an intermediate state between the JX bound outward-occluded state and the outward-open state. Based on the structural analysis, we propose a working model for HATs (Fig. 6): during the transition from the inward-facing conformation to the outward-facing conformation that might be triggered by substrate binding, the core domain rotates to the hash domain to close the inward gates. Meanwhile, the unwound regions of TM1 and TM6 undergo a conformational change to accommodate the substrate in the outward-facing conformation of the transporter. Then TM1b and TM6a undergo further rotation to induce the transition to the outward-open conformation and push the gating residue Phe252 away from the occluded configuration, triggering substrate release. During this process, the positions of TM1a and TM6b remain nearly unchanged, whereas TM2 and TM7 undergo a minor rotation. The ECD of the heavy chain 4F2hc also undergoes a rotation to help stabilize LAT1 during a transport cycle.

In summary, the high-resolution inhibitor-bound structures of the LAT1-4F2hc complex in an outward-occluded conformation reported in this work represent a major step towards a detailed understanding of the working mechanism of human HATs, and provides clues for the transporter-directed drug design.

## Supplementary information

supplementary information

Data Set 1

movie S1

movie S2

movie S3

## Data Availability

Atomic coordinates and cryo EM density maps of the LAT1-4F2hc complex bound with JX-075 (PDB: 7DSK; whole map: EMD-30835, TM-focused refined map: EMD-30836), JX-078 (PDB: 7DSL; whole map: EMD-30837, TM-focused refined map: EMD-30838), JX-119 (PDB: 7DSN; whole map: EMD-30839, TM-focused refined map: EMD-30840), Diiodo-Tyr (PDB: 7DSQ; whole map: EMD-30841, TM-focused refined map: EMD-30842) have been deposited to the Protein Data Bank (http://www.rcsb.org) and the Electron Microscopy Data Bank (https://www.ebi.ac.uk/pdbe/emdb/), respectively. Correspondence and requests for materials should be addressed to Q.Z. (zhouqiang@westlake.edu.cn) or K.H.A. (karl-heinz.altmann@pharma.ethz.ch).
