# Supplementary material for: Mechanism of substrate transport and inhibition of the human LAT1-4F2hc amino acid transporter
Source: Cell Discov. 2021 Mar 23;7:16. doi: 10.1038/s41421-021-00247-4 (PMC7988154; doi:10.1038/s41421-021-00247-4)
Supplement: Supplementary file 1 — supplementary information [file 41421_2021_247_MOESM1_ESM.pdf]

Material and Methods

Protein preparation

The full-length human cDNA of LAT1 (accession number: NM_003486.7) was subcloned into pCAG with N-terminal FLAG tag and 4F2hc (isoform b, accession number: NM_001012662.2) into pCAG with N-terminal 10×His tag. The mutations were generated by a standard two-step PCR.

For protein expression, LAT1 and 4F2hc were co-expressed in HEK 293F cells (Invitrogen) which were cultured in SMM 293T-II medium (Sino Biological Inc.) at 37 °C under 5% CO_2 in a Multitron-Pro shaker (Infors, 130 rpm). To transfect one liter of cells, 3 mg of polyethylenimines (PEIs) (Polysciences), 0.75 mg of the LAT1 plasmid and 0.75 mg of the 4F2hc plasmid were preincubated with 50 mL fresh medium for 15 mins and added into cell culture whose cell density reached 2.0×10^6/ml. 48 hours after transfection, cells were harvested by centrifugation at 3800×g for 10 mins and resuspended in a buffer containing 25 mM Tris (pH 8.0), 150 mM NaCl, and mixture of three protease inhibitors, aprotinin (1.3 μg/ml, AMRESCO), pepstatin (0.7 μg/ml, AMRESCO) and leupeptin (5 μg/ml, AMRESCO).

For protein purification, after incubating with 1% (w/v) LMNG (Anatrace) supplemented with 0.1% (w/v) cholesteryl hemisuccinate Tris salt (Anatrace) at 4 °C for 2 hours, cells were centrifugated at 18,700×g for 45 mins to remove cell debris. The supernatant was loaded onto anti-FLAG M2 affinity resin (Sigma). The resin was washed with the wash buffer containing 25 mM Tris (pH 8.0), 150 mM NaCl, 0.04% GDN (w/v), following by protein eluted with wash buffer plus 0.2 mg/mL FLAG peptide. Then elution of anti-FLAG M2 affinity resin was further purified with Ni-NTA affinity resin (Qiagen). Wash buffer and elution buffer of nickel resin was the wash buffer mentioned above plus 10 mM and 300 mM imidazole respectively. Then the protein complex was subjected to size-exclusion chromatography (Superose 6 Increase 10/300 GL, GE Healthcare) in buffer containing 25 mM Tris (pH 8.0), 150 mM NaCl and 0.02% GDN. The peak fractions were collected and concentrated for

EM analysis.

Synthesis of compounds

Please refer to data set for the details of the synthesis of the compounds JX-075, JX-078, JX-119.

Inhibition of [^{3}H]-L-leucine uptake

HT-29 cells were seeded at 60% confluency in a 96-well plate using complete culture medium and cultured until confluent. Cells were washed three times with 37 °C pre-warmed Na^+-free Hank's balanced salt solution (HBBS) containing 125 mM choline-Cl, 25 mM HEPES, 4.8 mM KCl, 1.2 mM $MgSO_4$, 1.2 mM KH_2PO_4, 1.3 mM $CaCl_2$ and 5.6 mM glucose (pH 7.4) and further incubated in the same buffer at 37 °C for 7 min. L-leucine uptake was measured for 3 min at 37 °C in the same buffer containing 30 μM L-[^{3}H]leucine (100 Ci/mmol) and different concentrations of test compounds that were added as stock solutions in DMSO. For JX-119, the compound solution needed to be vortexed thoroughly as it was poorly soluble in DMSO. Uptake was terminated by removing the solution followed by three washings with ice-cold Na^+-free HBBS. Cells were lysed and mixed with Microscint20 (Perkin-Elmer Life Sciences). The radioactivity was measured with a scintillation counter (TopCount NXT, Perkin-Elmer Life Sciences). The IC_{50} of the compounds was determined using the four-parameter logistic to fit the dose-response curves in Graphpad Prism 9.

Induction of [^{3}H]-L-leucine efflux (assessment of substrate properties)

The same protocol as for the assessment of uptake inhibition was used with the following differences after the initial washing and starvation step. Cells were preloaded for 5 min at 37 °C in the Na^+-free HBBS containing 30 μM L-[^{3}H]leucine (100 Ci/mmol). After washing three times with Na^+-free HBBS (4 °C), efflux of radioactivity was induced by incubation in the presence or absence of indicated

concentrations of test compounds for 10 min at 37 °C. The medium was then collected and its radioactivity was counted. The cells were washed three times with ice-cold Na^+-free HBBS. Cells were lysed and mixed with Microscint20 (Perkin-Elmer Life Sciences). The radioactivity was measured with a scintillation counter (TopCount NXT, Perkin-Elmer Life Sciences). The L-[^{3}H]leucine efflux values were expressed as percentage radioactivity (radioactivity of medium)/(radioactivity of the medium + radioactivity of the cells).

Cryo-EM sample preparation and data acquisition

The purified LAT1-4F2hc complex was concentrated to ~ 10 mg/mL and incubated with 50 µM the JX series inhibitors or 1 mM 3,5-Diiodo-L-tyrosine (Diiodo-Tyr) for 1 hours, respectively, before being applied to the grids. Aliquots (3.3 µL) of the protein complex were placed on glow-discharged holey carbon grids (Quantifoil Cu R1.2/1.3). The grids were blotted for 3s or 3.5 s and flash-frozen in liquid ethane cooled by liquid nitrogen with Vitrobot (Mark IV, Thermo Fisher Scientific). The cryo-EM samples were transferred to a Titan Krios operating at 300 kV equipped with a Gatan K3 detector and GIF Quantum energy filter. Movie stacks were automatically collected using AutoEMation [1], with a slit width of 20 eV on the energy filter and a defocus range from -1.2 µm to -2.2 µm in super-resolution mode at a nominal magnification of 81,000×. Each stack was exposed for 2.56 s with an exposure time of 0.08 s per frame, resulting in a total of 32 frames per stack. The total dose rate was approximately 50 $e^-/Å^2$ for each stack. The stacks were motion corrected with MotionCor2 [2] and binned 2-fold, resulting in a pixel size of 1.087 Å/pixel. Meanwhile, dose weighting was performed [3]. The defocus values were estimated with Gctf [4].

Data processing

Particles were automatically picked from manually selected micrographs using Relion 3 [5-8]. After 2D classification, good particles were selected and subjected to a global angular searching 3D classification using the cryo EM map of the LAT1-4F2hc complex (EMD-9722) as the initial model with only one class. For each of the last

several iterations of the global angular searching 3D classification, a local angular searching 3D classification was performed, during which the particles were classified into 4 classes. Non-redundant good particles were selected from the local angular searching 3D classification. Then, these selected particles were subjected to multi-reference 3D classification, local defocus correction, 3D auto-refinement and post-processing. To further improve the map quality in the TM region, focused 3D classification and auto-refinement were performed by applying appropriate mask on the TM region.

The 2D classification, 3D classification and 3D auto-refinement were performed with Relion 3. The resolution was estimated with the gold-standard Fourier shell correlation 0.143 criterion [9] with high-resolution noise substitution [10].

Model building and structure refinement

The atomic models of the LAT1-4F2hc complex bound with inhibitors were built based on the corresponding cryo-EM maps using the previous structure of the LAT1-4F2hc complex (PDB ID: 6IRT) as a template, which were further manually built in Coot [11]. Each residue was manually checked with the chemical properties considered during model building.

Structure real space refinement was performed with Phenix [12] with secondary structure and geometry restraints to prevent structure overfitting. To monitor the overfitting of the model, the model was refined against one of the two independent half maps from the gold-standard 3D refinement approach. Then, the refined model was tested against the other map. Statistics associated with data collection, 3D reconstruction and model refinement can be found in Table S1.

Supplementary Table S1 | Summary of data collection and model statistics for LAT1-4F2hc bound with inhibitors

**Data collection**				
EM equipment	Titan Krios (Thermo Fisher Scientific)			
Voltage (kV)	300			
Detector	Gatan K3 Summit			
Energy filter	Gatan GIF Quantum, 20 eV slit			
Pixel size (Å)	1.087			
Electron dose (e-/Å2)	50			
Defocus range (μm)	-1.2 ~ -2.2			
Sample	LAT1+JX-078	LAT1+JX-075	LAT1+JX-119	LAT1+Diiodo-Tyr
Number of collected micrographs	4,548	3,655	3,123	2,753
Number of selected micrographs	3,942	3,089	3,109	2,348
**3D Reconstruction**				
Software	Relion 3.0			
Number of used particles (Overall)	579,107	378,156	641,590	93,151
Number of used particles (TM)	388,358	244,966	314,179	70,531
Resolution (Å) (Overall/ TM)	2.9/ 2.7	2.9/ 2.7	3.1/ 2.8	3.4/ 3.3
Symmetry	C1			
Map sharpening B-factor (Å2)	-150			
**Refinement**				
Software	Phenix			
Cell dimensions				
a=b=c (Å)	278.272			
α=β=γ (˚)	90			
Model composition				
Protein residues	935	935	935	935
Side chains assigned	935	935	935	935
Sugar	8	8	8	8
inhibitor	1	1	1	1
Phospholipid	1	1	1	1
Cholesterol hemisuccinate	2	2	2	2
Water	8	12	17	15
R.m.s deviations				
Bonds length (Å)	0.010	0.005	0.006	0.007
Bonds Angle (˚)	1.144	0.965	0.996	1.113
Ramachandran plot statistics (%)				
Preferred	90.66	91.41	93.02	90.43
Allowed	8.81	8.27	6.66	9.03
Outlier	0.54	0.32	0.32	0.54

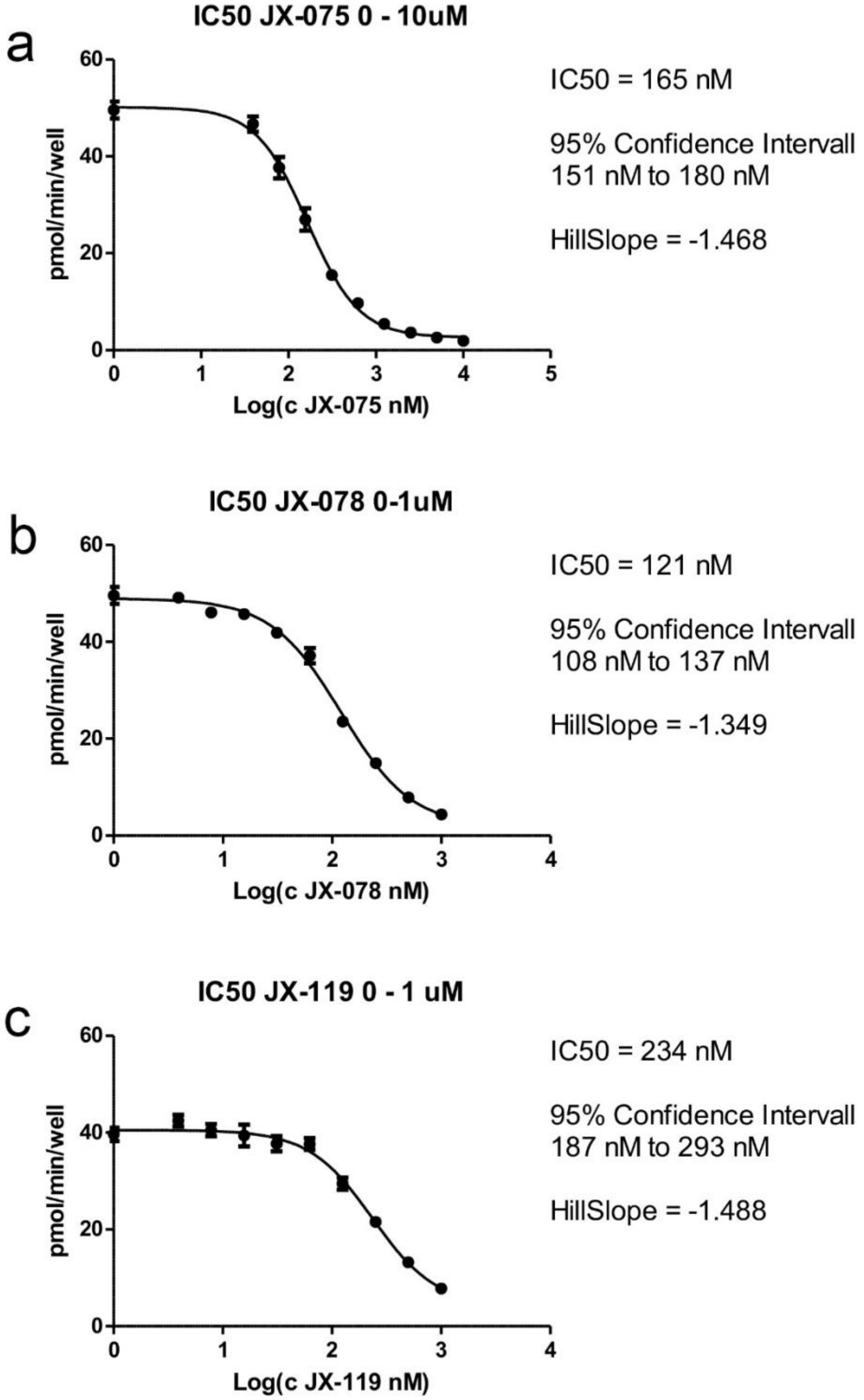

Supplementary Fig. S1. Inhibition of uptake of [^{3}H]-L-leucine into HT-29 cells by JX-075 (a), JX-078 (b), and JX-119 (c).

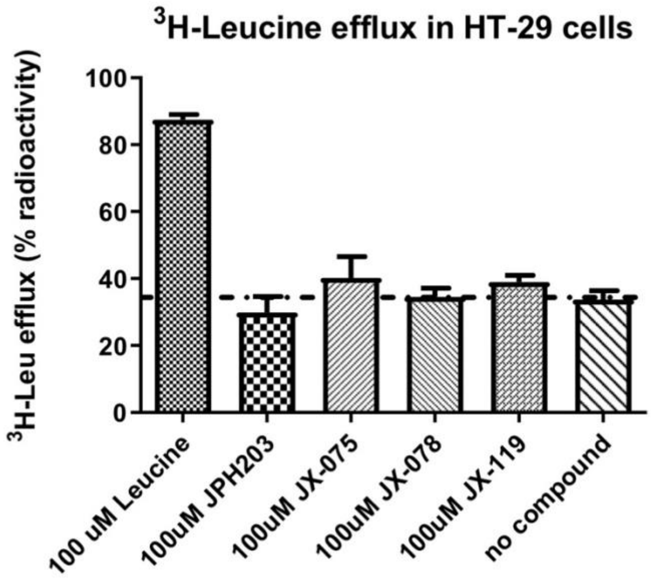

Supplementary Fig. S2. JX-075, JX-078, and JX-119 did not induce measurable leucine efflux at a concentration of up to 100 μM. The same result was obtained with the known LAT1 inhibitor JPH-203. Based on these data, none of the compounds is a substrate for LAT1.

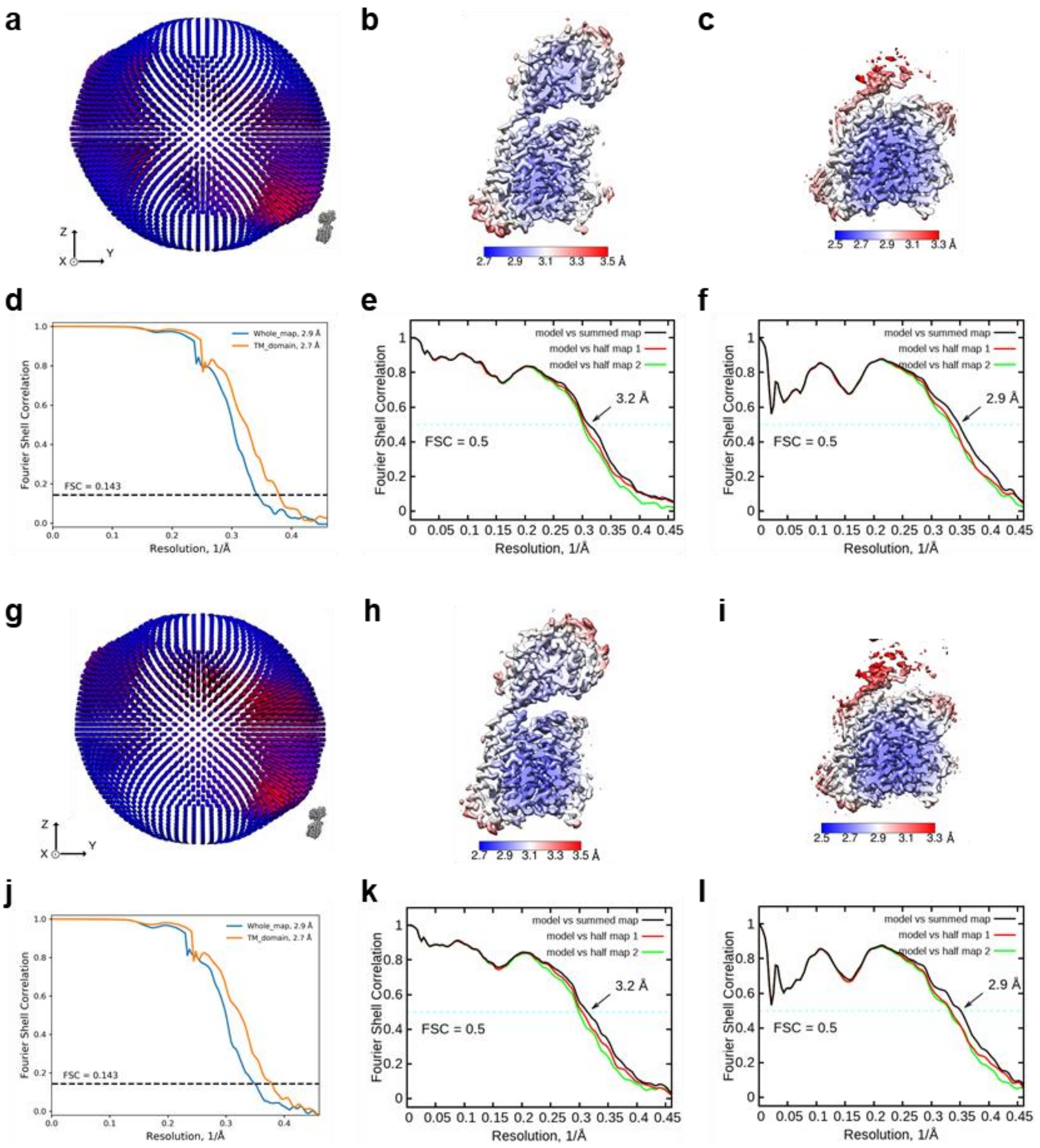

Supplementary Fig. S3. Cryo-EM analysis of the LAT1-4F2hc bound with inhibitors. a Euler angle distribution of the LAT1-4F2hc bound with JX-078. **b, c** are local resolution maps for the 3D EM reconstruction of the overall structure and TM region of the LAT1-4F2hc bound with JX-078, respectively. **d** FSC curve for the 3D refinement of the overall structure (blue) and TM region (orange) of the LAT1-4F2hc bound with JX-078. **e** FSC curve of the refined model of the LAT1-4F2hc bound with JX-078 versus the overall map that it is refined against (black); of the model refined against the first half map versus the same map (red); and of the model refined against

the first half map versus the second half map (green). The small difference between the red and green curves indicates that the refinement of the atomic coordinates did not suffer from overfitting. **f** FSC curve of TM region, which is same to the **(e)**. **g-l** are same to **(a-f)**, but for the LAT1-4F2hc bound with JX-075.

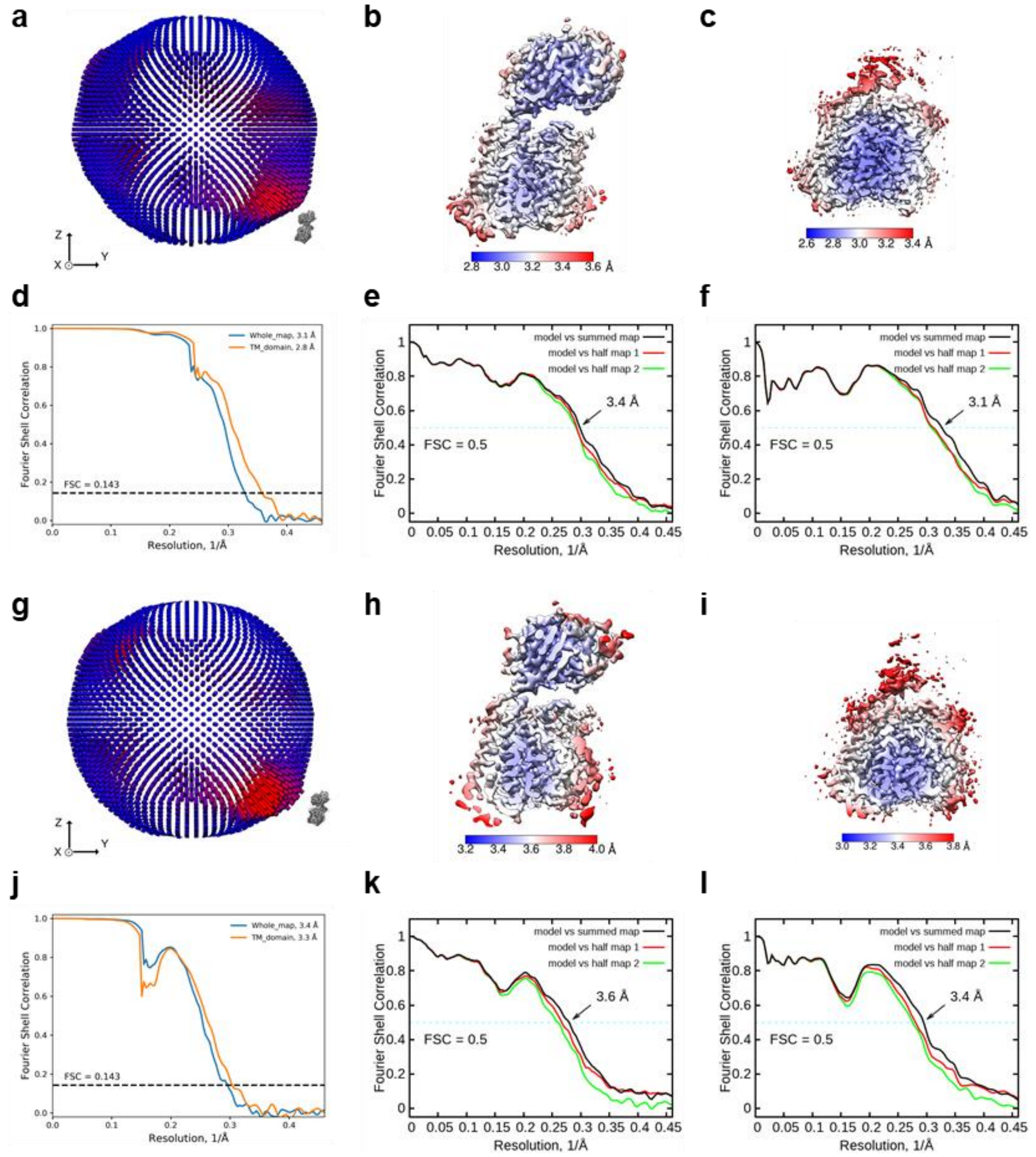

Supplementary Fig. S4. Cryo-EM analysis of the LAT1-4F2hc bound with inhibitors. a-f are same to **(a-f)** of **Supplementary Fig. S3**, but for the LAT1-4F2hc bound with JX-119. **g-l** are same to **(a-f)** of **Supplementary Fig. S3**, but for the LAT1-4F2hc bound with Diiodo-Tyr.

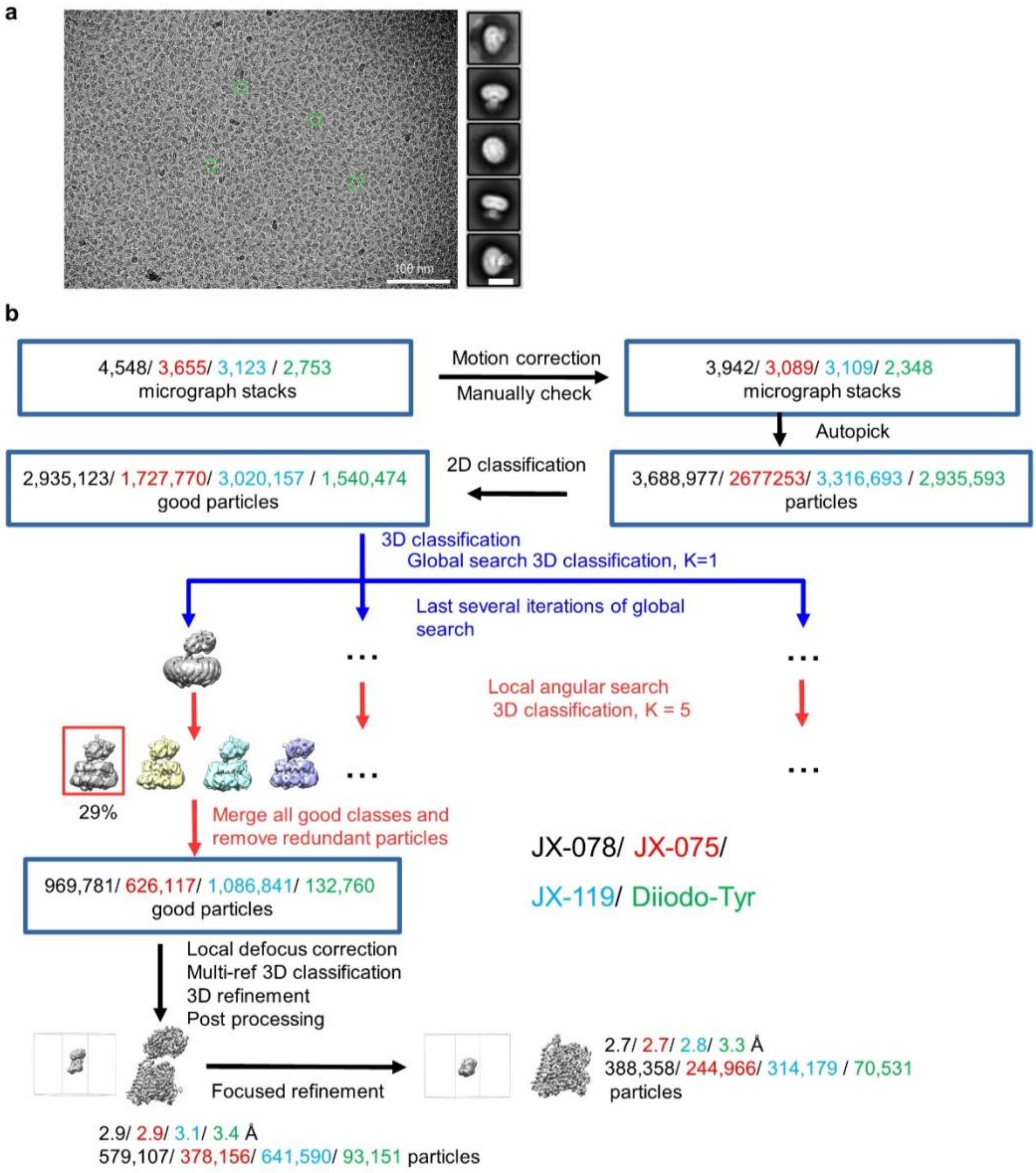

Supplementary Fig. S5. Representative cryo-EM image and Flowchart of the LAT1-4F2hc bound with inhibitors for cryo-EM data processing. a Representative electron micrograph and 2D class averages. The scale bar in 2D class averages represent 10 nm. **b** Please refer to the 'Data Processing' section in Methods for details. K, number of classifications.

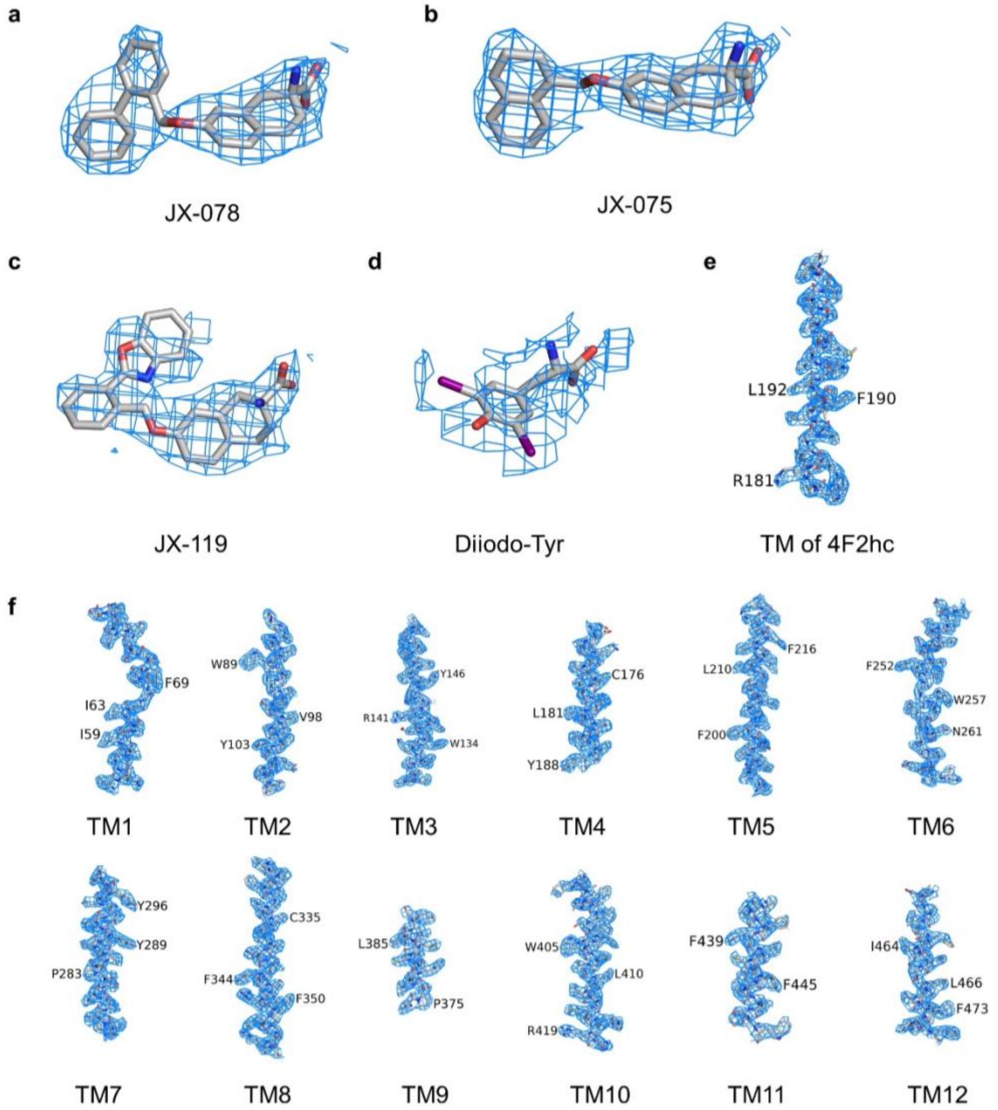

Supplementary Fig. S6 Cryo EM density maps of the LAT1-4F2hc bound with inhibitors. a-d Cryo EM density maps for the inhibitors bound with LAT1-4F2hc shown at threshold of 6 σ. **e** Cryo EM density maps for the transmembrane helix of 4F2hc shown at threshold of 8 σ. **f** Cryo EM density maps for TM segments of the outward occluded LAT1 shown at threshold of 8 σ.

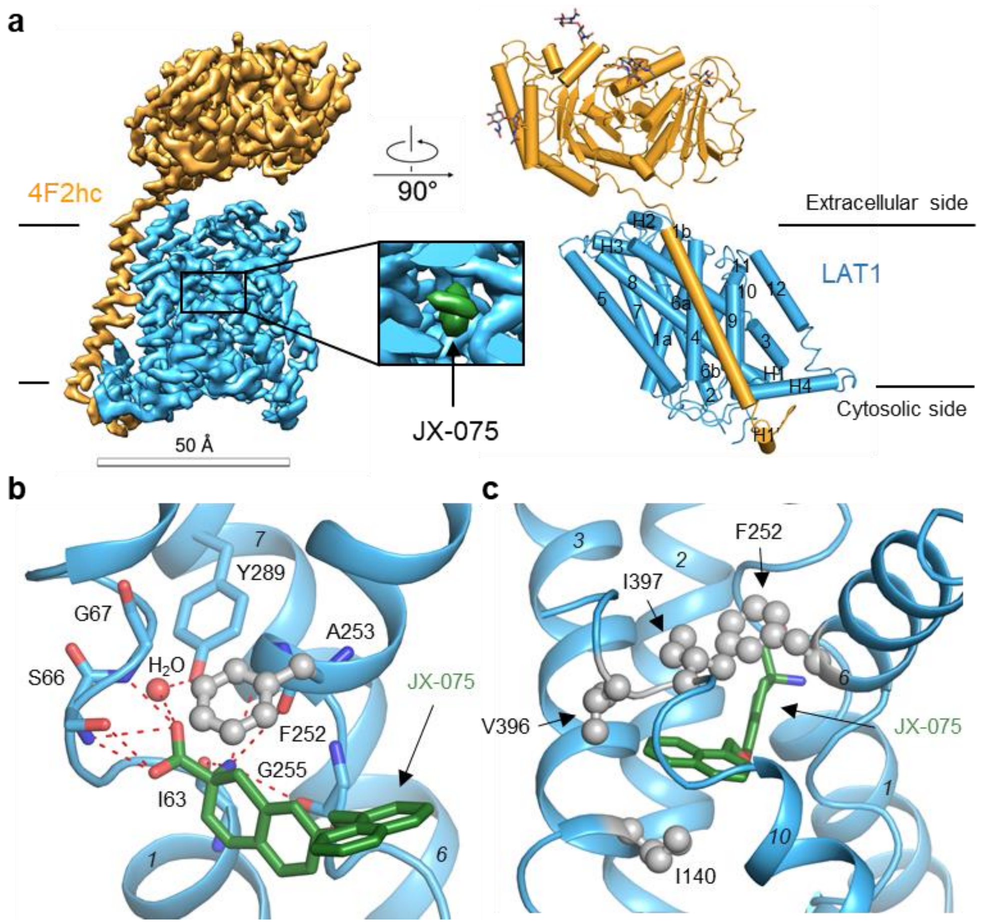

Supplementary Fig. S7. Overall structure of the LAT1-4F2hc bound with JX-075. a The overall structure of the LAT1-4F2hc bound with JX-075. The left is the cryo-EM map of the complex, the overall structure of the complex is in the right. The glycosylation moieties are shown as sticks. ECD, extracellular domain; H, helix; TM, transmembrane domain. 4F2hc and LAT1 are colored orange and blue, respectively. JX-075 are shown as black green. **b** and **c** The details of JX-075 binding mode in LAT1 are shown here, which the residues participating in interaction are indicated as sticks (hydrogen bonds network) and spheres (hydrophobic interaction).

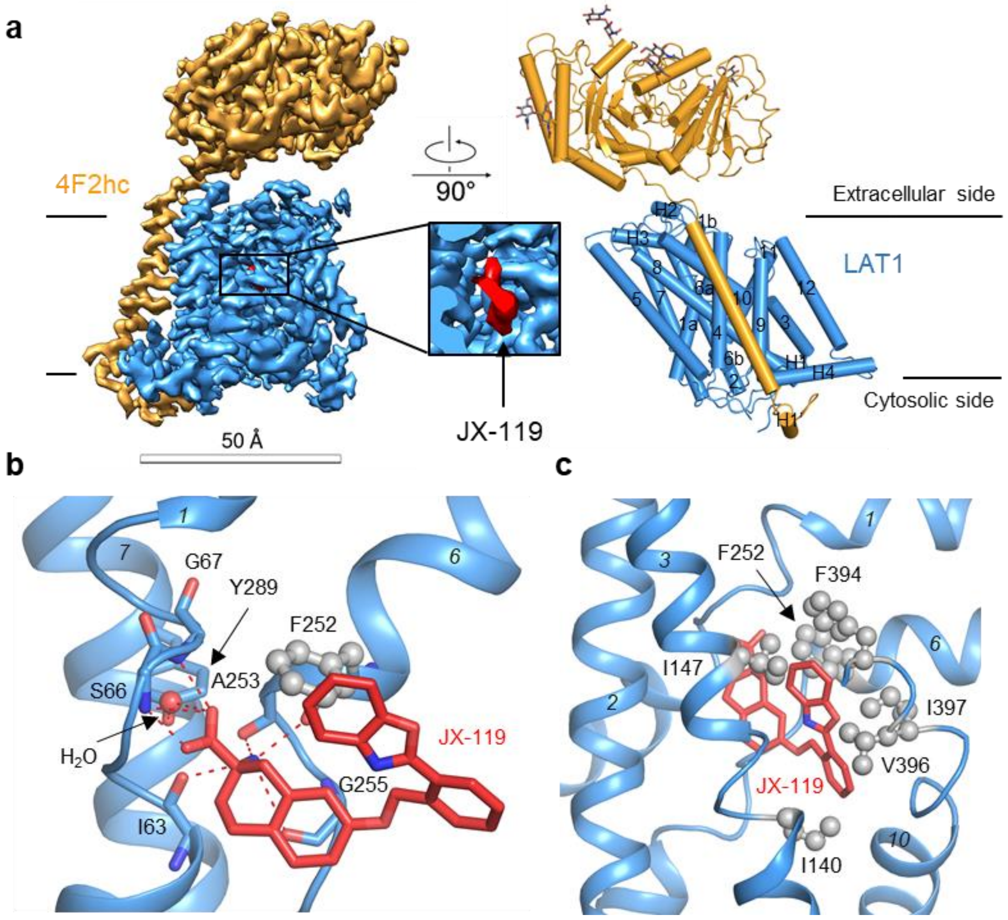

Supplementary Fig. S8. Overall structure of the LAT1-4F2hc bound with JX-119. **a** The overall structure of the LAT1-4F2hc bound with JX-119. The left is the cryo-EM map of the complex, the overall structure of the complex is in the right. The glycosylation moieties are shown as sticks. ECD, extracellular domain; H, helix; TM, transmembrane domain. 4F2hc and LAT1 are colored orange and blue, respectively. JX-119 are shown as red. **b** and **c** The details of JX-119 binding mode in LAT1 are shown here, which the residues participating in interaction are indicated as sticks (hydrogen bonds network) and spheres (hydrophobic interaction).

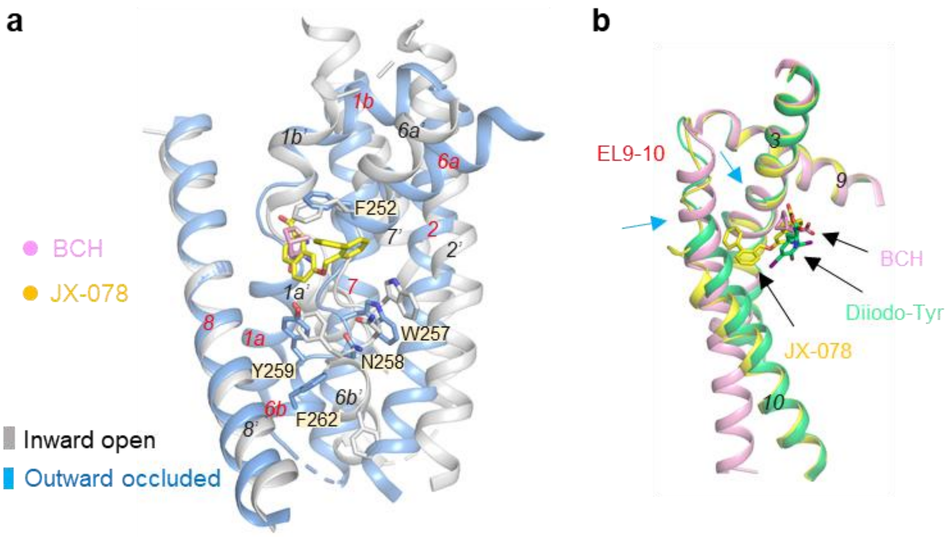

Supplementary Fig. S9. Structural comparison between inward open LAT1 and outward occluded LAT1. a Comparison of the intracellular vestibule of the transport path between inward open LAT1 and outward occluded LAT1. Some of the key residues that are involved in transport are shown as sticks. **b** The TM3, EL9-10 and TM10 of outward occluded LAT1s (yellow and lemon green) could appear broken regions comparing the inward open LAT1 (pink). The broken regions indicate as blue arrows. TM, transmembrane domain; EL, extracellular loop.

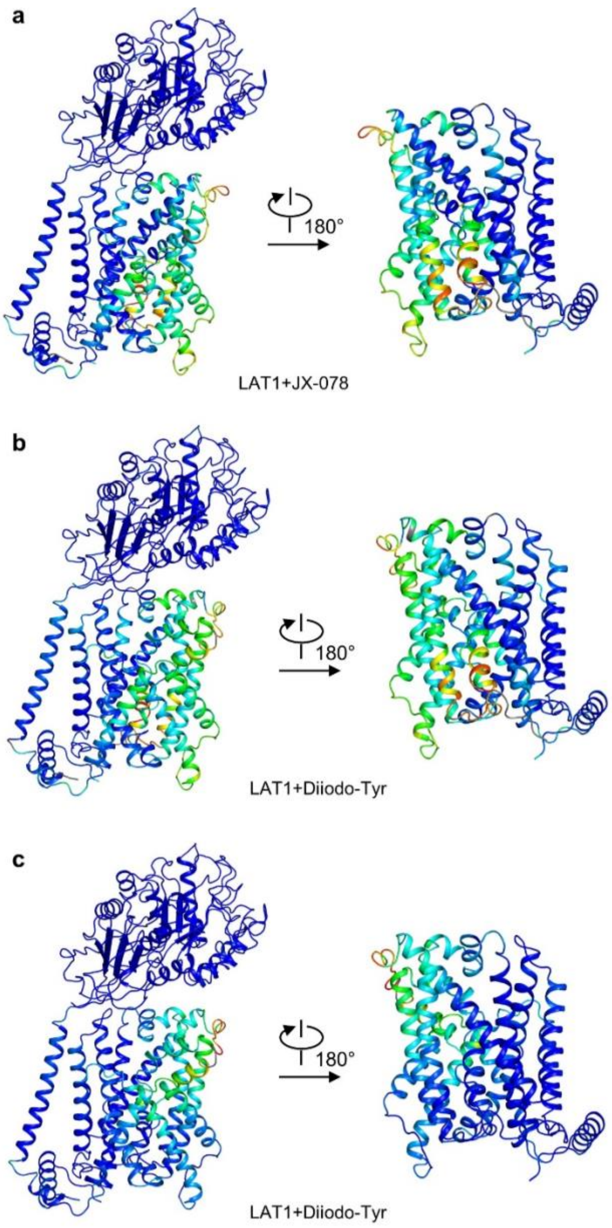

Supplementary Fig. S10. Comparison of different conformations of LAT1 through RMSD analysis. a Comparison of overall structures between LAT1+JX-078 (outward occluded) and LAT1+BCH (inward open) through residue-by-residue RMSD analysis. Conformational change shown by color with a rainbow spectrum. Blue represents the lowest RMSD and red represents the largest RMSD. **b** Comparison of overall structures between LAT1+diiodo-Tyr (outward occluded) and LAT1+BCH (inward open) through residue-by-residue RMSD analysis. **c** Comparison of overall structures between LAT1+diiodo-Tyr (outward occluded) and LAT1+JX-078 (outward occluded) through residue-by-residue RMSD analysis.

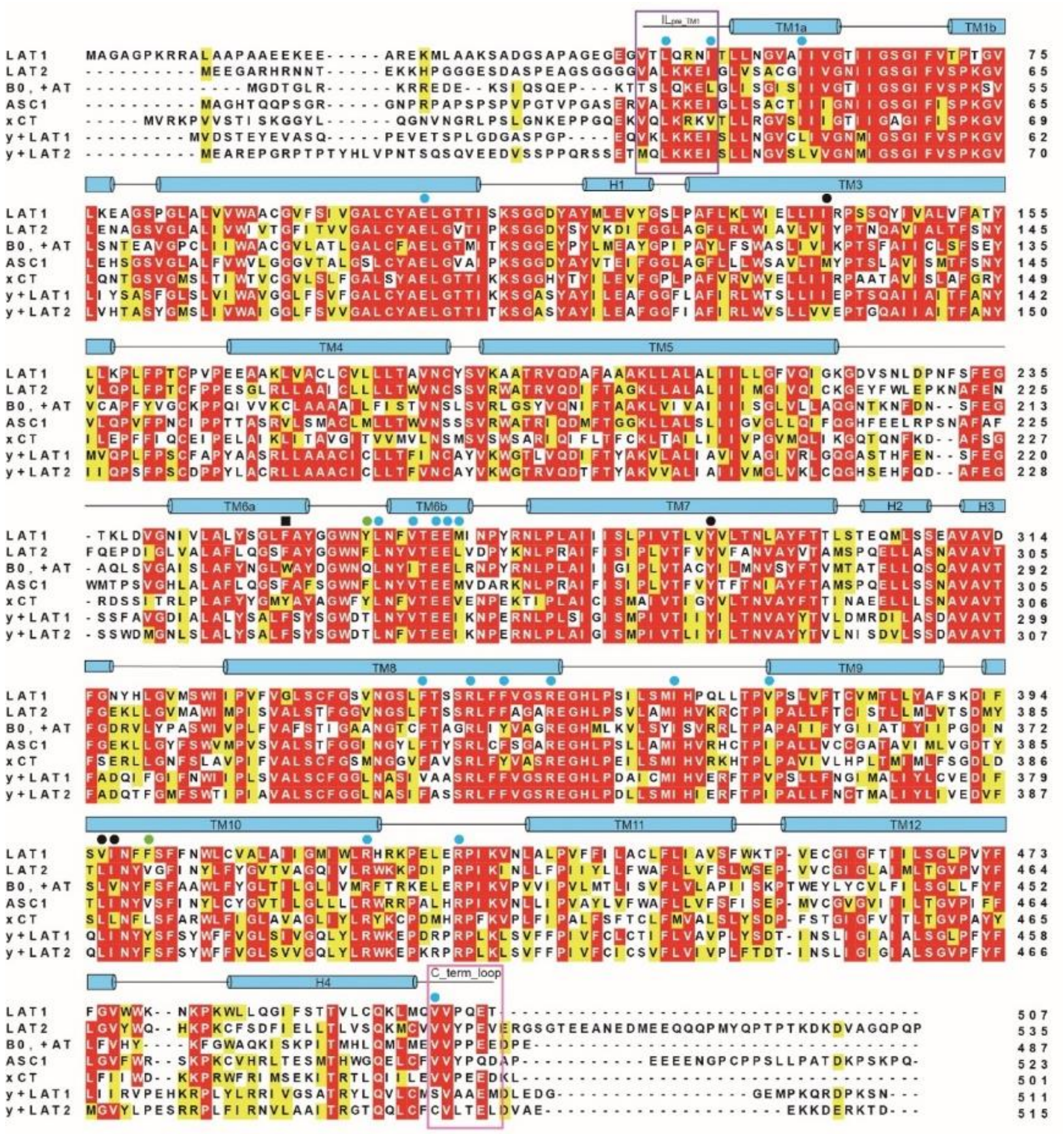

Supplementary Fig. S11. Sequence alignment of LAT1 homologues. The sequences are aligned using clustalX. The seven aligned sequences are LAT1, LAT2, $b^{0,+}$AT, ASC1, xCT, y^{+}LAT1, y^{+}LAT2 from Homo sapiens. Amino acids that are identical or conserved in at least four sequences are coloured red or yellow, respectively. The secondary structural elements of LAT1 are indicated above the sequence alignment. The gating residue Phe252 is labelled with a solid black square. The residues that are involved in interaction with JX-078 are marked with solid black circles. The resides important for interaction with Diiodo-Tyr are indicated by solid green circles. The residues that stabilize the outward-facing conformation are marked with solid blue circles. IL_{pre_TM1} and C_term_loop are outlined with purple and pink boxes, respectively. The UNIPROT

IDs of aligned sequences are listed as below. LAT1: Q01650; LAT2: Q9UHI5; $b^{0,+}$AT: P82251; ASC1: Q9NS82; xCT: Q9UPY5; y^+LAT1: Q9UM01; y^+LAT2: Q92536.

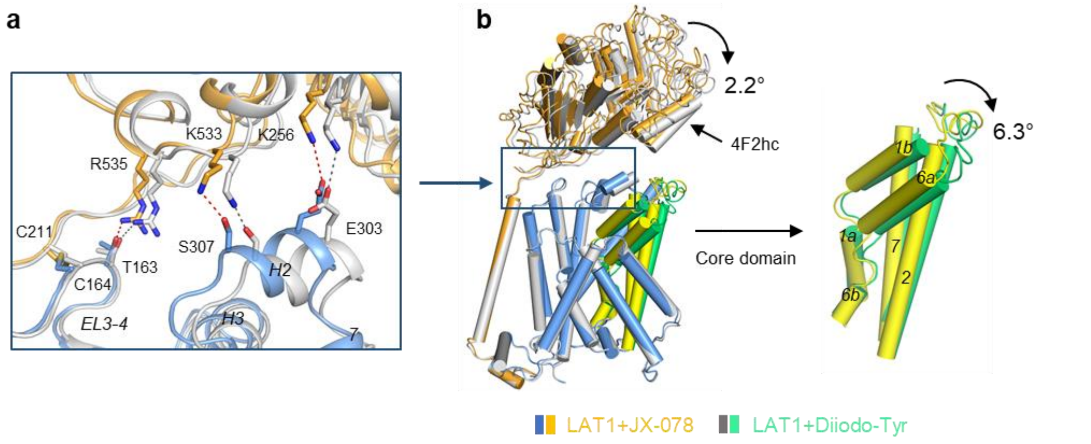

Supplementary Fig. S12. Conformational changes between two outward occluded conformations of the LAT1-4F2hc complex. a The interactions at the extracellular interface between LAT1 and 4F2hc are conserved between the JX-078 bound structure and the Diiodo-Tyr bound structure. **b** Comparison between the outward occluded structures of the LAT1-4F2hc complex bound with JX-078 and bound with Diiodo-Tyr. The core domain of LAT1 bound with Diiodo-Tyr rotates for 6.3 degrees compared with that bound with JX-078. The extracellular interface between LAT1 and 4F2hc is boxed with a deep blue box, the details of which are shown in (a)**.**

Supplementary Movies S1-S3. The conformational change of LAT1 between different conformations. Movie S1 is the conformational change of LAT1 between inward-facing (PDB ID: 6IRT) conformation and the JX-078 bound outward-facing conformation. The structures are superimposed according to their hash domains with the heavy chain 4F2hc shown in wheat and LAT1 shown in rainbow from blue nitrogen terminus to red carboxyl terminus. Movie S2 or S3 are same as movie S1, but between the inward-facing conformation and the Diiodo-Tyr bound outward-facing conformation, or between the JX-078 bound outward-facing conformation and the Diiodo-Tyr bound outward-facing conformation, respectively.

References

1 Lei, J. & Frank, J. Automated acquisition of cryo-electron micrographs for single particle reconstruction on an FEI Tecnai electron microscope. *Journal of structural biology* **150**, 69-80, doi:10.1016/j.jsb.2005.01.002 (2005).

2 Zheng, S. Q. *et al.* MotionCor2: anisotropic correction of beam-induced motion for improved cryo-electron microscopy. *Nature methods* **14**, 331-332, doi:10.1038/nmeth.4193 (2017).

3 Grant, T. & Grigorieff, N. Measuring the optimal exposure for single particle cryo-EM using a 2.6 A reconstruction of rotavirus VP6. *eLife* **4**, e06980, doi:10.7554/eLife.06980 (2015).

4 Zhang, K. Gctf: Real-time CTF determination and correction. *Journal of structural biology* **193**, 1-12, doi:10.1016/j.jsb.2015.11.003 (2016).

5 Kimanius, D., Forsberg, B. O., Scheres, S. H. & Lindahl, E. Accelerated cryo-EM structure determination with parallelisation using GPUs in RELION-2. *eLife* **5**, doi:10.7554/eLife.18722 (2016).

6 Scheres, S. H. A Bayesian view on cryo-EM structure determination. *Journal of molecular biology* **415**, 406-418, doi:10.1016/j.jmb.2011.11.010 (2012).

7 Scheres, S. H. RELION: implementation of a Bayesian approach to cryo-EM structure determination. *Journal of structural biology* **180**, 519-530, doi:10.1016/j.jsb.2012.09.006 (2012).

8 Scheres, S. H. Semi-automated selection of cryo-EM particles in RELION-1.3. *Journal of structural biology* **189**, 114-122, doi:10.1016/j.jsb.2014.11.010 (2015).

9 Rosenthal, P. B. & Henderson, R. Optimal determination of particle orientation, absolute hand, and contrast loss in single-particle electron cryomicroscopy. *Journal of molecular biology* **333**, 721-745 (2003).

10 Chen, S. *et al.* High-resolution noise substitution to measure overfitting and validate resolution in 3D structure determination by single particle electron cryomicroscopy. *Ultramicroscopy* **135**, 24-35, doi:10.1016/j.ultramic.2013.06.004 (2013).

11 Emsley, P., Lohkamp, B., Scott, W. G. & Cowtan, K. Features and development of Coot.

Acta crystallographica. Section D, Biological crystallography **66**, 486-501, doi:10.1107/S0907444910007493 (2010).

12 Adams, P. D. *et al.* PHENIX: a comprehensive Python-based system for macromolecular structure solution. *Acta crystallographica. Section D, Biological crystallography* **66**, 213-221, doi:10.1107/S0907444909052925 (2010).
