# Supplementary material for: Mechanism of substrate transport and inhibition of the human LAT1-4F2hc amino acid transporter
Source: Cell Discov. 2021 Mar 23;7:16. doi: 10.1038/s41421-021-00247-4 (PMC7988154; doi:10.1038/s41421-021-00247-4)
Supplement: Supplementary file 2 — Data Set 1 [file 41421_2021_247_MOESM2_ESM.pdf]

Synthesis of JX-075, JX-078, and JX-119

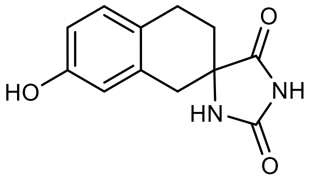

JX-046

7'-Hydroxy-3',4'-dihydro-1'H-spiro[imidazolidine-4,2'-naphthalene]-2,5-dione (JX-046): To a suspension of 7-hydroxy-2-tetralone (131 mg, 0.809 mmol, 1.0 equiv) in EtOH/H_2O (4:1, 2.6 mL) in a microwave vial were added $(NH_4)_2CO_3$ (700 mg, 7.28 mmol, 9.0 equiv) and KCN (79 mg, 1.2 mmol, 1.5 equiv) and the vial was sealed. After 30 min of microwave irradiation at 80 °C, the reaction mixture was diluted with H_2O, the solution was extracted with EtOAc (3 × 200 mL) and the combined organic phases were washed with brine (3 × 100 mL). The organic layer was dried over $MgSO_4$, filtered, and concentrated under reduced pressure to yield crude **JX-046** (172 mg, 0.742 mmol, 92%) as a beige brown solid. This material was used in the following step without purification.

TLC (SiO_2; CH_2Cl_2/MeOH 90:10, UV, $KMnO_4$ stain): $R_f = 0.34$.

^{1}H-NMR (400 MHz, CD_3OD): δ (ppm) = 6.96 (d, J = 8.2 Hz, 1H), 6.61 (dd, J = 8.3, 2.6 Hz, 1H), 6.53 (d, J = 2.6 Hz, 1H), 3.20 (d, J = 16.6 Hz, 1H), 2.94 – 2.84 (m, 2H), 2.72 (dd, J = 16.8, 2.0 Hz, 1H), 2.08 (ddd, J = 13.2, 9.4, 9.4 Hz, 1H), 1.89 (dddd, J = 13.2, 4.7, 4.6, 2.1 Hz, 1H).

^{13}C-NMR (101 MHz, CD_3OD): δ (ppm) = 180.63, 161.17, 156.75, 134.36, 130.82, 126.52, 116.24, 115.12, 63.30, 38.33, 31.81, 25.26.

HR-MS (ESI): Calcd for $C_{12}H_{13}N_2O_3$ $[M+H]^+$, 233.0921 *m/z*; found 233.0921 *m/z*.

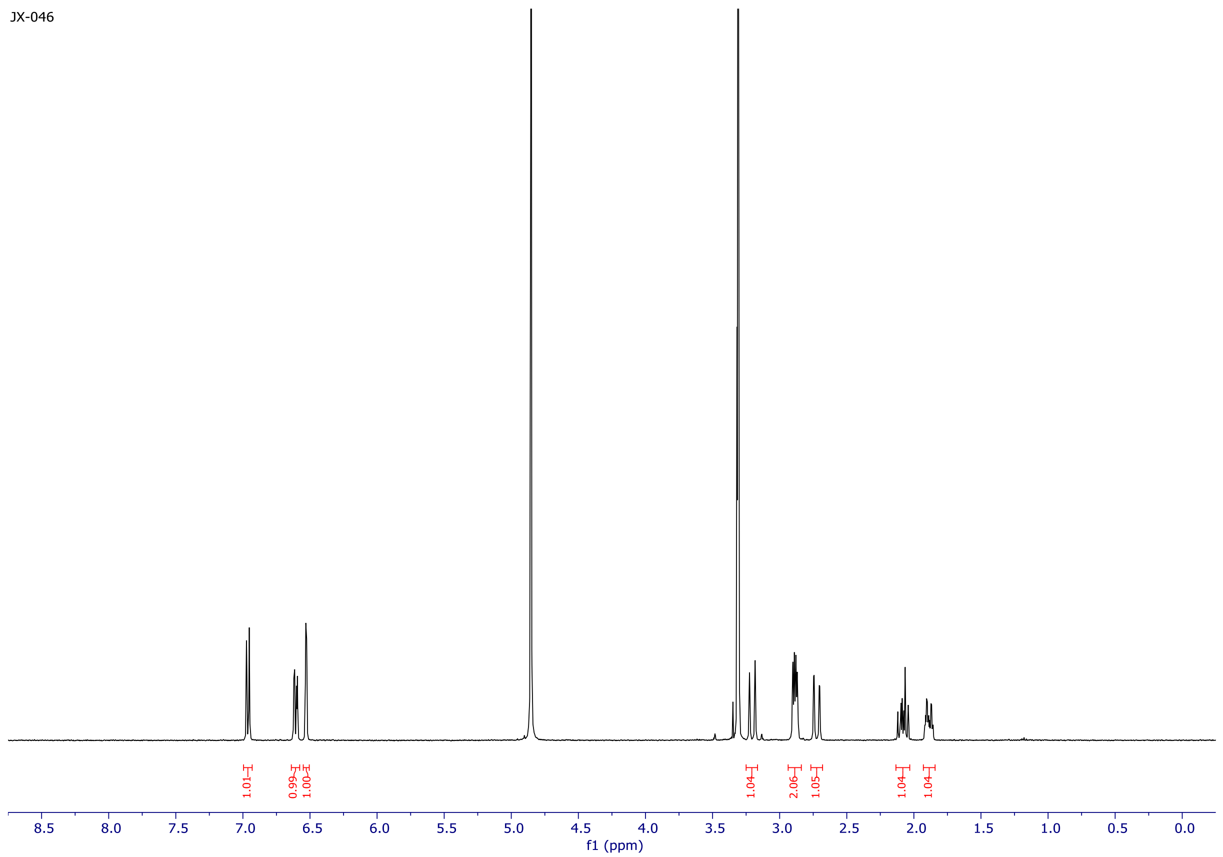

JX-046
1.01
0.99
1.00
1.04
2.06
1.05
1.04
1.04
8.5 8.0 7.5 7.0 6.5 6.0 5.5 5.0 4.5 4.0 3.5 3.0 2.5 2.0 1.5 1.0 0.5 0.0
f1 (ppm)

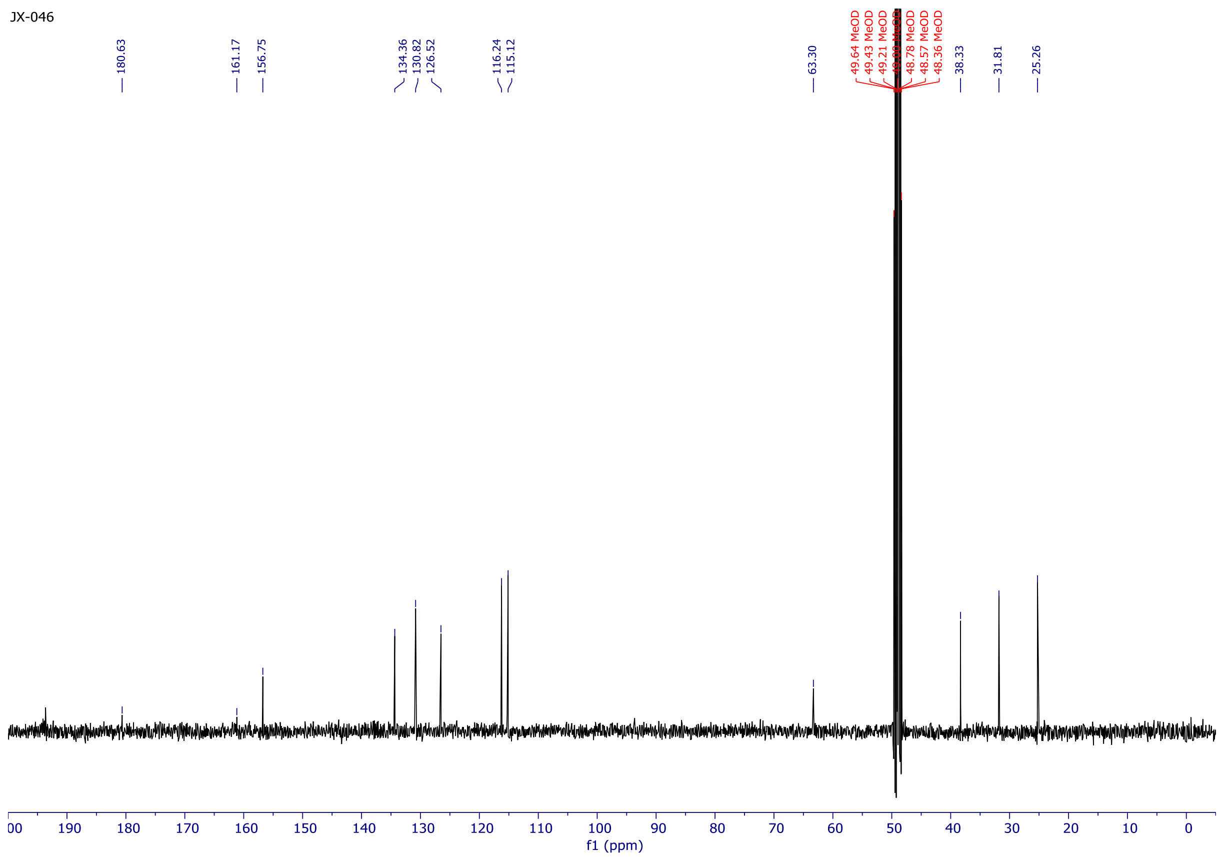

JX-046
180.63
161.17
156.75
134.36
130.82
126.52
116.24
115.12
63.30
49.64 MeOD
49.43 MeOD
49.21 MeOD
48.78 MeOD
48.57 MeOD
48.36 MeOD
38.33
31.81
25.26
f1 (ppm)

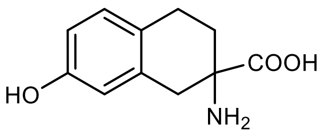

JX-048

2-Amino-7-hydroxy-1,2,3,4-tetrahydronaphthalene-2-carboxylic acid (JX-046): To a suspension of **JX-046** (225 mg, 0.969 mmol, 1.0 equiv) in H_2O (10.0 mL) in a microwave vial was added $Ba(OH)_2{\cdot}8H_2O$ (1.528 g, 4.844 mmol, 5.0 equiv) and the vial was sealed. After 1 h of microwave irradiation at 150 °C, the reaction mixture was concentrated under reduced pressure to obtain 1.33 g of crude product. 798 mg of this material was purified by flash column chromatography ($CHCl_3$/MeOH/H_2O 70:30:5 → 60:40:8) to yield **JX-048** (120 mg, 0.579 mmol, quant.) as a beige solid.

TLC (SiO_2; $CHCl_3$/MeOH/H_2O 70:30:5, UV, $KMnO_4$ stain): R_f = 0.16.

^{1}H-NMR (400 MHz, $(CD_3)_2SO$): δ (ppm) = 9.21 (brs, 1H), 7.62 (brs, 2H), 6.85 (d, J = 8.3 Hz, 1H), 6.53 (dd, J = 8.2, 2.5 Hz, 1H), 6.46 (d, J = 2.5 Hz, 1H), 3.22 (d, J = 17.3 Hz, 1H), 2.75 – 2.53 (m, 3H), 2.03 (ddd, J = 13.5, 9.7, 6.6 Hz, 1H), 1.81 (ddd, J = 13.3, 5.1, 5.1 Hz, 1H).

^{13}C-NMR (101 MHz, $(CD_3)_2SO$): δ (ppm) = 172.72, 155.40, 134.54, 129.25, 125.06, 114.99, 113.25, 58.18, 36.02, 29.74, 23.94.

IR (solid): υ (cm^{-1}) = 3182, 3049, 2947, 2826, 2746, 2564, 1639, 1603, 1586, 1514, 1500, 1458, 1441, 1396, 1335, 1286, 1270, 1246, 1227, 1169, 1152, 1110 ,1088, 950, 876, 853, 818, 775, 750, 705, 692, 620, 607, 545.

MS (ESI): Calcd for $C_{11}H_{14}NO_3$ $[M+H]^+$, 208.1 *m/z*; found 208.2 *m/z* (direct injection). No molecular ion was detectable in the HRMS, probably solubility issues.

JX-048

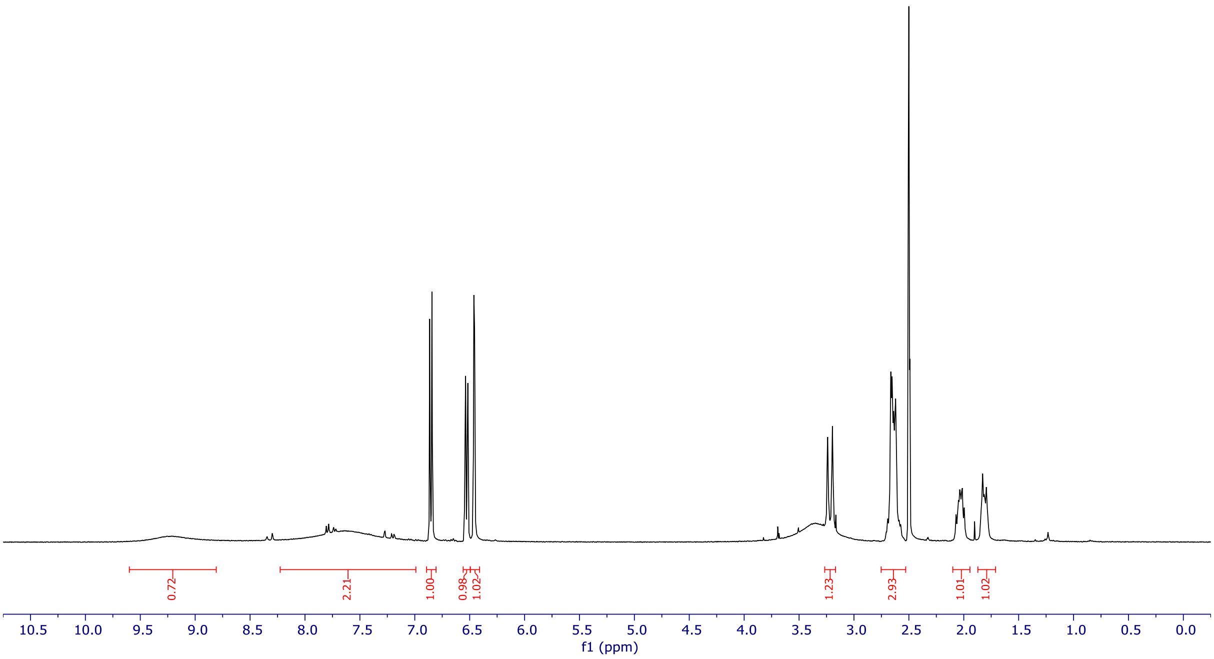
0.72
2.21
1.00
0.98
1.02
1.23
2.93
1.01
1.02
10.5 10.0 9.5 9.0 8.5 8.0 7.5 7.0 6.5 6.0 5.5 5.0 4.5 4.0 3.5 3.0 2.5 2.0 1.5 1.0 0.5 0.0
f1 (ppm)

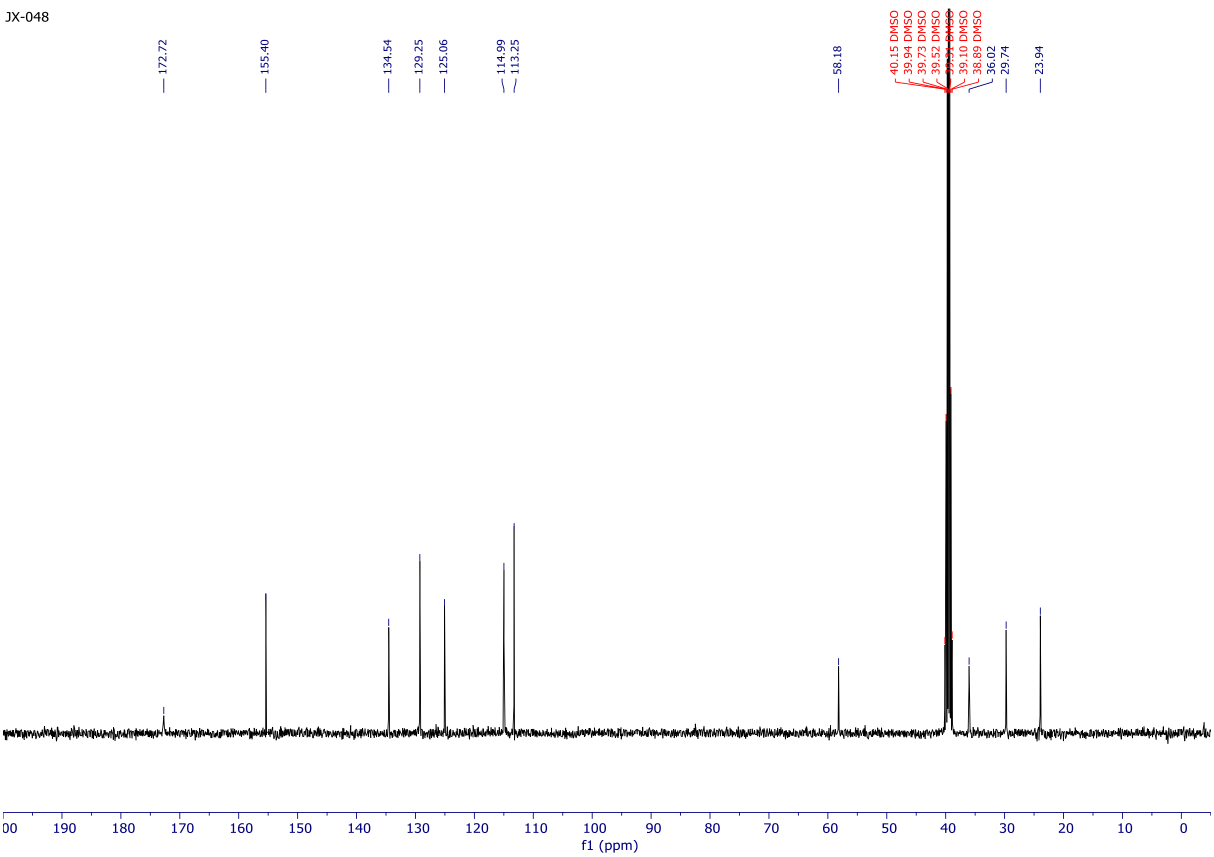
JX-048
172.72
155.40
134.54
129.25
125.06
114.99
113.25
58.18
40.15 DMSO
39.94 DMSO
39.73 DMSO
39.52 DMSO
39.31 DMSO
39.10 DMSO
38.89 DMSO
36.02
29.74
23.94
f1 (ppm)

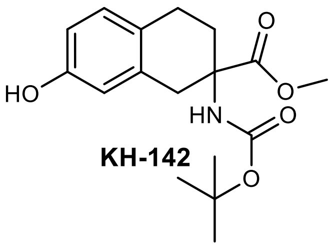

KH-142

Methyl 2-((tert-butoxycarbonyl)amino)-7-hydroxy-1,2,3,4-tetrahydro-naphthalene-2-carboxylate (KH-142): To a solution of **JX-048** (500 mg, 2.41 mmol, 1.0 equiv) in dry MeOH (10.0 mL) was added $SOCl_2$ (1.0 mL, 14 mmol, 5.7 equiv) dropwise under an argon atmosphere over 3 min and the mixture was heated to reflux. After 3 h, the solvent was evaporated under reduced pressure and the residue was redissolved in dry MeOH (10.0 mL) and Et_3N (1.3 mL, 9.3 mmol, 3.9 equiv) was added followed by Boc_2O (944 mg, 4.32 mmol, 1.8 equiv) and the mixture was stirred at rt under argon atmosphere for 20 h. The solvent was then evaporated under reduced pressure and the residue was diluted with H_2O (20 mL), the pH was adjusted to 2 with 1 M HCl (2.0 mL), and the mixture was extracted with EtOAc (2 × 20 mL). The combined organic phases were washed with H_2O (2x), dried with $MgSO_4$, filtered, and concentrated under reduced pressure. The crude product was purified by flash column chromatography (CH_2Cl_2/acetone 9:1) to yield **KH-142** (505 mg, 1.57 mmol, 65%) as a beige solid.

TLC (SiO_2; CH_2Cl_2/MeOH 95:5, UV, $KMnO_4$ stain): $R_f = 0.34$.

^{1}H-NMR (400 MHz, $CDCl_3$): δ (ppm) = 6.96 (d, J = 8.3 Hz, 1H), 6.65 (dd, J = 8.3, 2.7 Hz, 1H), 6.54 (d, J = 2.7 Hz, 1H), 4.82 (brs, 1H), 4.43 (brs, 1H), 3.76 (s, 3H), 3.25 (d, J = 16.8 Hz, 1H), 2.90 (d, J = 16.9 Hz, 1H), 2.80 – 2.72 (m, 2H), 2.49 – 2.35 (m, 1H), 2.08 (ddd, J = 13.5, 9.1, 9.1 Hz, 1H), 1.42 (s, 9H).

^{13}C-NMR (101 MHz, $CDCl_3$): δ (ppm) = 174.76, 155.22, 154.11, 130.02, 126.86, 115.68, 114.11, 76.79, 57.94, 52.66, 38.21, 29.06, 28.40, 24.49. The signal for one quaternary C is missing from the spectrum.

IR (thin film): υ (cm^{-1}) = 3360, 2978, 1693, 1615, 1591, 1505, 1454, 1392, 1367, 1254, 1164, 1109, 1065, 914, 852, 817, 734.

HR-MS (ESI): Calcd for $C_{17}H_{23}NNaO_5$ $[M+Na]^+$, 344.1468 *m/z*; found, 344.1462 *m/z*.

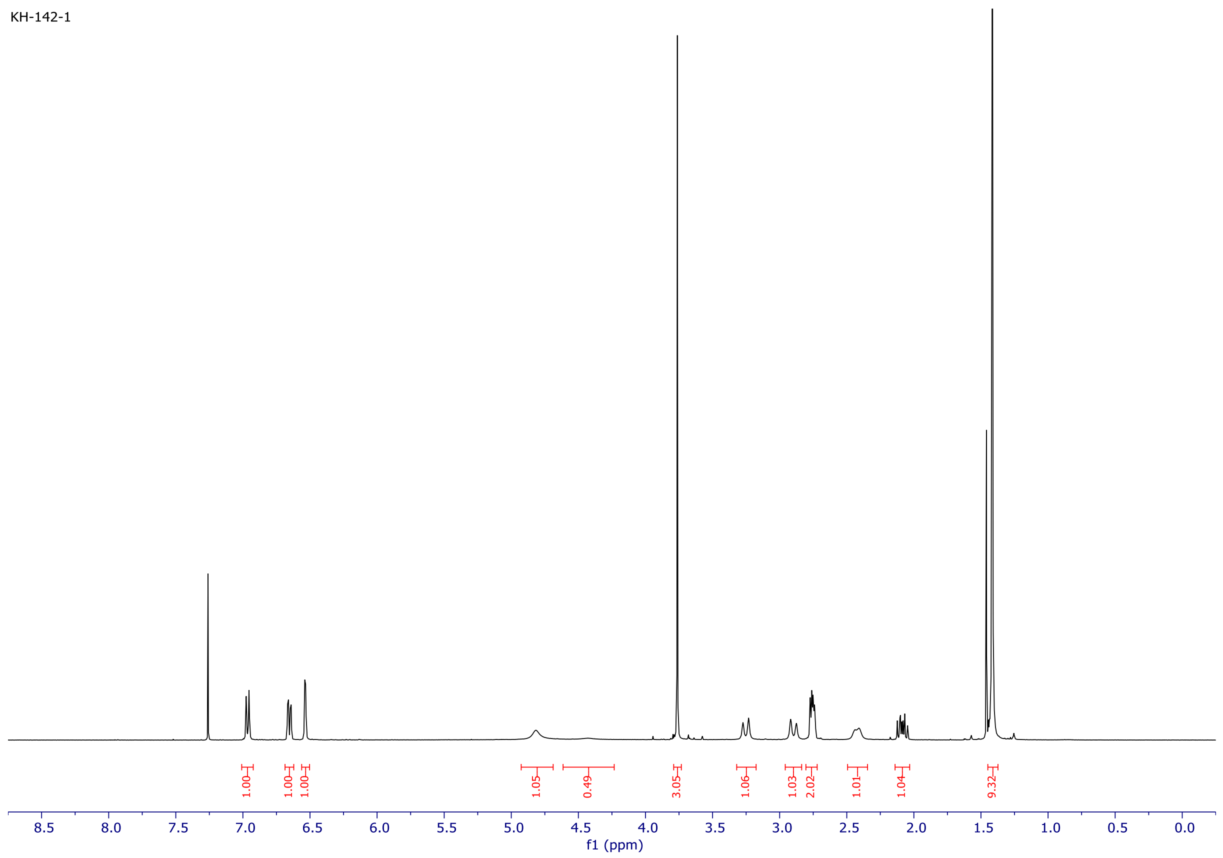

KH-142-1
1.00
1.00
1.00
1.05
0.49
3.05
1.06
1.03
2.02
1.01
1.04
9.32
8.5
8.0
7.5
7.0
6.5
6.0
5.5
5.0
4.5
4.0
3.5
3.0
2.5
2.0
1.5
1.0
0.5
0.0
f1 (ppm)

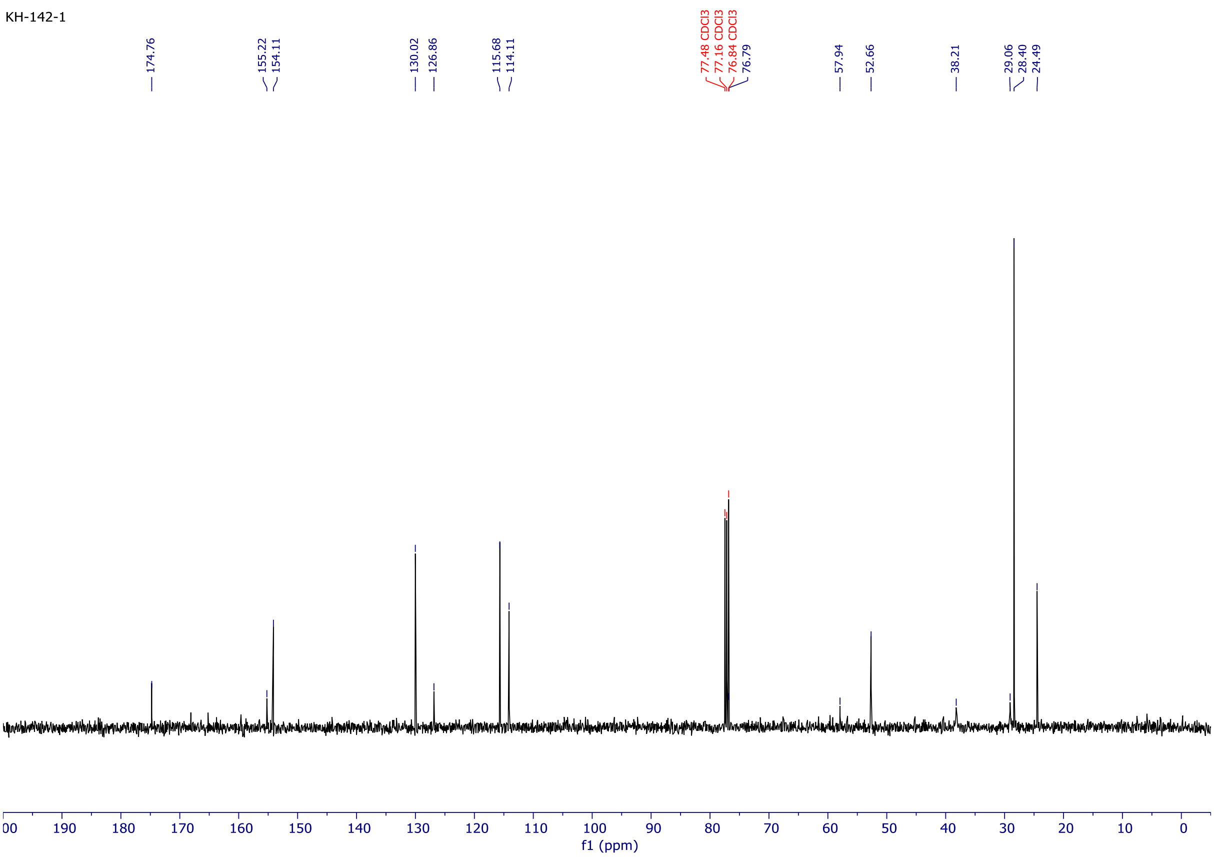

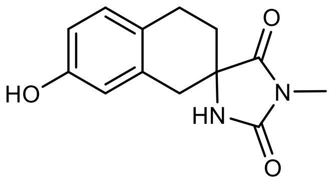

JX-059

7'-Hydroxy-1-methyl-3',4'-dihydro-1'H-spiro[imidazolidine-4,2'-naphthalene]-2,5-dione (JX-059): To a stirred solution of **JX-046** (642 mg, 2.76 mmol, 1.0 equiv) in dry DMF (18 mL) was added K_2CO_3 (764 mg, 5.52 mmol, 2.0 equiv) in one portion at rt under argon and the mixture was continued to stir. After 10 min, MeI (0.17 mL, 2.8 mmol, 1.0 equiv) was added dropwise and the reaction mixture was heated to 35 °C. After 15 h, the solution was concentrated under reduced pressure, the residue was redissolved in EtOAc (150 mL), and the solution was washed with brine (3 × 100 mL). The organic layer was dried with $MgSO_4$, filtered, and concentrated under reduced pressure. The crude product was purified by flash column chromatography (hexane/EtOAc 4:6 and CH_2Cl_2/MeOH 96:4) to yield **JX-059** (519 mg, 76%) as beige solid.

TLC (SiO_2; CH_2Cl_2/MeOH 95:5, UV, $KMnO_4$ stain): R_f = 0.18.

mp: 215.1-215.6 °C.

^{1}H-NMR (400 MHz, $(CD_3)_2SO$): δ (ppm) = 9.11 (s, 1H), 8.54 (s, 1H), 6.91 (d, *J* = 8.3 Hz, 1H), 6.56 (dd, *J* = 8.2, 2.6 Hz, 1H), 6.47 (d, *J* = 2.5 Hz, 1H), 3.05 (d, *J* = 17.0 Hz, 1H), 2.85 (s, 3H), 2.82 – 2.71 (m, 2H), 2.65 (d, *J* = 16.9 Hz, 1H), 1.91 (ddd, *J* = 13.1, 10.5, 6.9 Hz, 1H), 1.81 – 1.71 (m, 1H).

^{13}C-NMR (101 MHz, $(CD_3)_2SO$): δ (ppm) = 176.67, 156.05, 155.38, 133.34, 129.45, 124.84, 114.98, 113.69, 59.83, 36.99, 30.34, 24.16, 23.85.

IR (thin film): υ (cm^{-1}) = 3287, 2928, 1768, 1615, 1590, 1504, 1466, 1396, 1348, 1313, 1273, 1224, 1155, 1021, 1001, 764, 718, 642, 589.

HR-MS (ESI): Calcd for $C_{13}H_{14}N_2NaO_3$ $[M+Na]^+$, 269.0897 *m/z*; found, 269.0897 *m/z*.

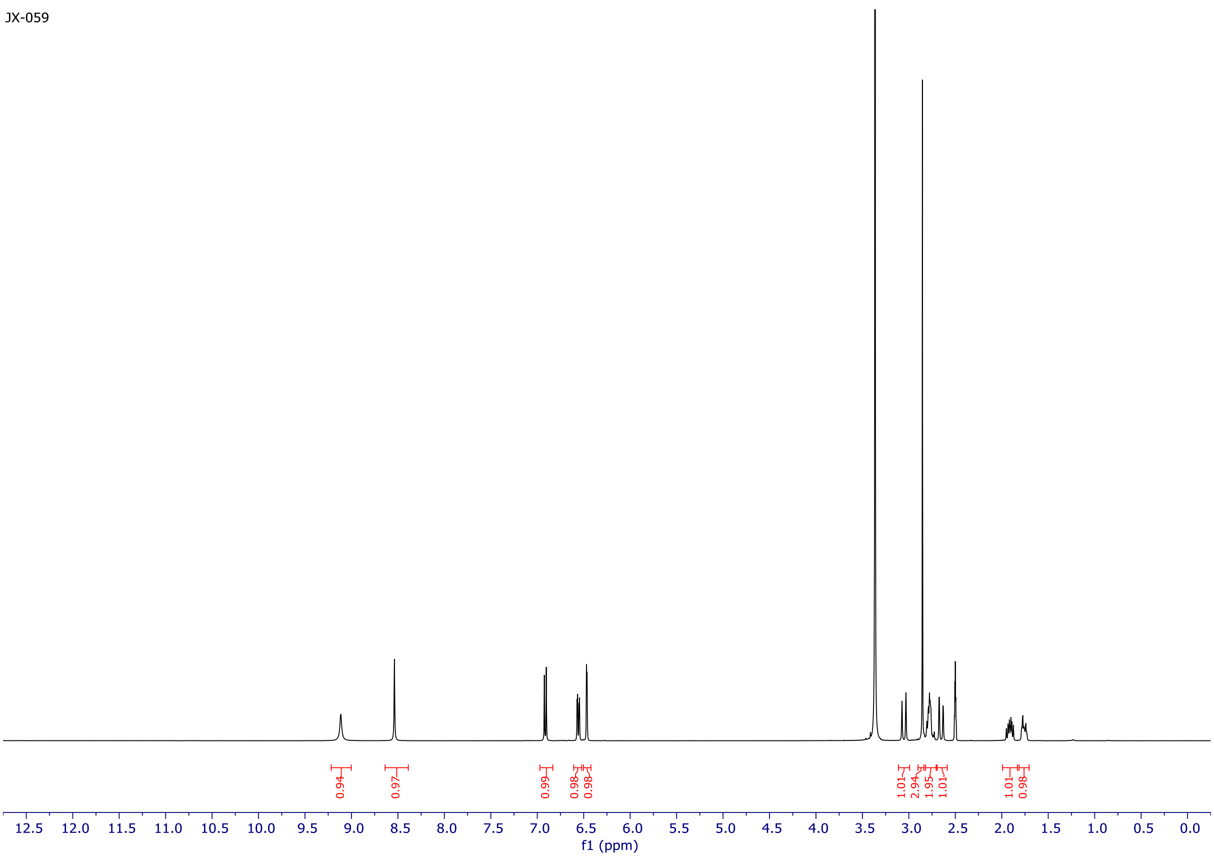
JX-059
0.94
0.97
0.99
0.98
0.98
1.01
2.94
1.95
1.01
1.01
0.98
12.5 12.0 11.5 11.0 10.5 10.0 9.5 9.0 8.5 8.0 7.5 7.0 6.5 6.0 5.5 5.0 4.5 4.0 3.5 3.0 2.5 2.0 1.5 1.0 0.5 0.0
f1 (ppm)

JX-059

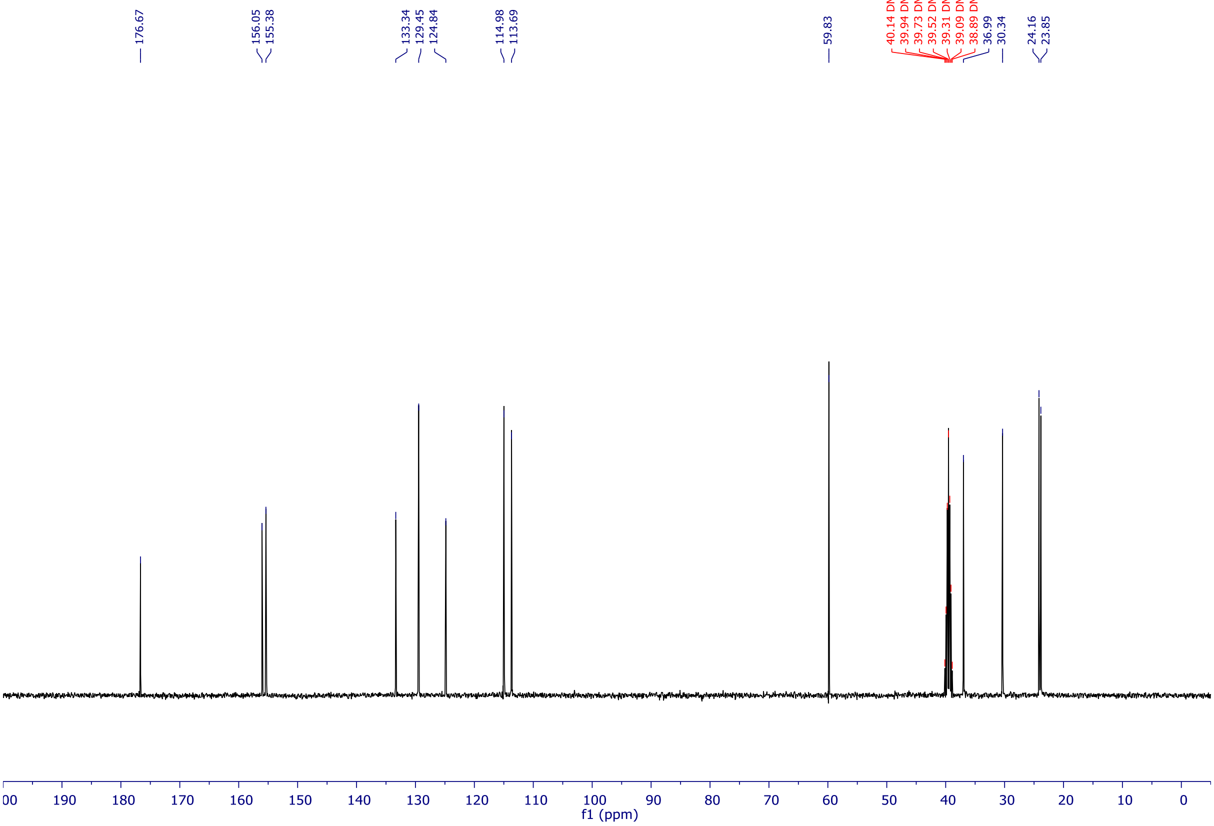
176.67
156.05
155.38
133.34
129.45
124.84
114.98
113.69
59.83
40.14 DMSO
39.94 DMSO
39.73 DMSO
39.52 DMSO
39.31 DMSO
39.09 DMSO
38.89 DMSO
36.99
30.34
24.16
23.85
00
190
180
170
160
150
140
130
120
110
100
90
80
70
60
50
40
30
20
10
0
f1 (ppm)

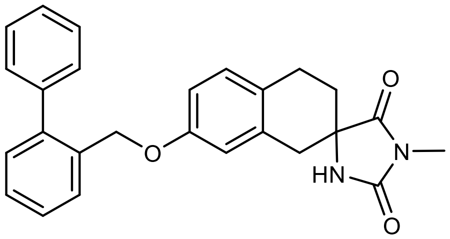

JX-065

7'-([1,1'-Biphenyl]-2-ylmethoxy)-1-methyl-3',4'-dihydro-1'H-spiro[imidazolidine-4,2'-naphthalene]-2,5-dione (JX-065): To a stirred solution of **JX-059** (52 mg, 0.21 mmol, 1.0 equiv) in dry DMF (2.0 mL) was added Cs_2CO_3 (103 mg, 0.317 mmol, 1.5 equiv) in one portion at rt under argon and the mixture was continued to stir. After 10 min, 2-phenylbenzyl bromide (0.040 mL, 0.22 mmol, 1.05 equiv) was added dropwise and the reaction mixture was heated to 60 °C. After 15 h, the reaction was allowed to cool to rt, the mixture was diluted with EtOAc (100 mL), and the solution was washed with brine (3 × 25 mL). The organic layer was dried with $MgSO_4$, filtered, and concentrated under reduced pressure. The crude product was purified by flash column chromatography (CH_2Cl_2/MeOH 98:2) to yield **JX-065** (76 mg, 87%) as a white beige solid.

TLC (SiO_2; CH_2Cl_2/MeOH 98:2, UV, $KMnO_4$ stain): $R_f = 0.43$.

mp: 112.8-116.8 °C.

^{1}H-NMR (400 MHz, $CDCl_3$): δ (ppm) = 7.60 (dd, J = 5.5, 3.6 Hz, 1H), 7.48 – 7.30 (m, 8H), 7.01 (d, J = 8.5 Hz, 1H), 6.71 (dd, J = 8.5, 2.7 Hz, 1H), 6.52 (d, J = 2.6 Hz, 1H), 5.59 (s, 1H), 4.90 (s, 2H), 3.30 (d, J = 16.4 Hz, 1H), 3.04 (s, 3H), 2.96 (ddd, J = 17.4, 6.7, 2.7 Hz, 1H), 2.81 (ddd, J = 16.4, 11.4, 5.6 Hz, 1H), 2.63 (dd, J = 16.6, 2.3 Hz, 1H), 2.19 (ddd, J = 13.4, 12.0, 6.7 Hz, 1H), 1.87 (dddd, J = 13.4, 6.2, 2.6, 2.6 Hz, 1H).

^{13}C-NMR (101 MHz, $CDCl_3$): δ (ppm) = 176.56, 157.31, 156.64, 141.98, 140.56, 134.06, 132.82, 130.26, 130.04, 129.39, 129.28, 128.42, 128.29, 127.83, 127.48, 125.99, 115.16, 114.41, 68.25, 60.86, 37.80, 30.29, 24.84, 24.24.

IR (thin film): υ (cm^{-1}) = 3291, 2928, 1772, 1704, 1610, 1502, 1456, 1394, 1347, 1308, 1265, 1225, 1160, 1115, 1018, 999, 909, 750, 730, 704, 647, 585.

HR-MS (ESI): Calcd for $C_{26}H_{25}N_2O_3$ $[M+H]^+$, 413.1860 *m/z*; found, 413.1862 *m/z*.

JX-065

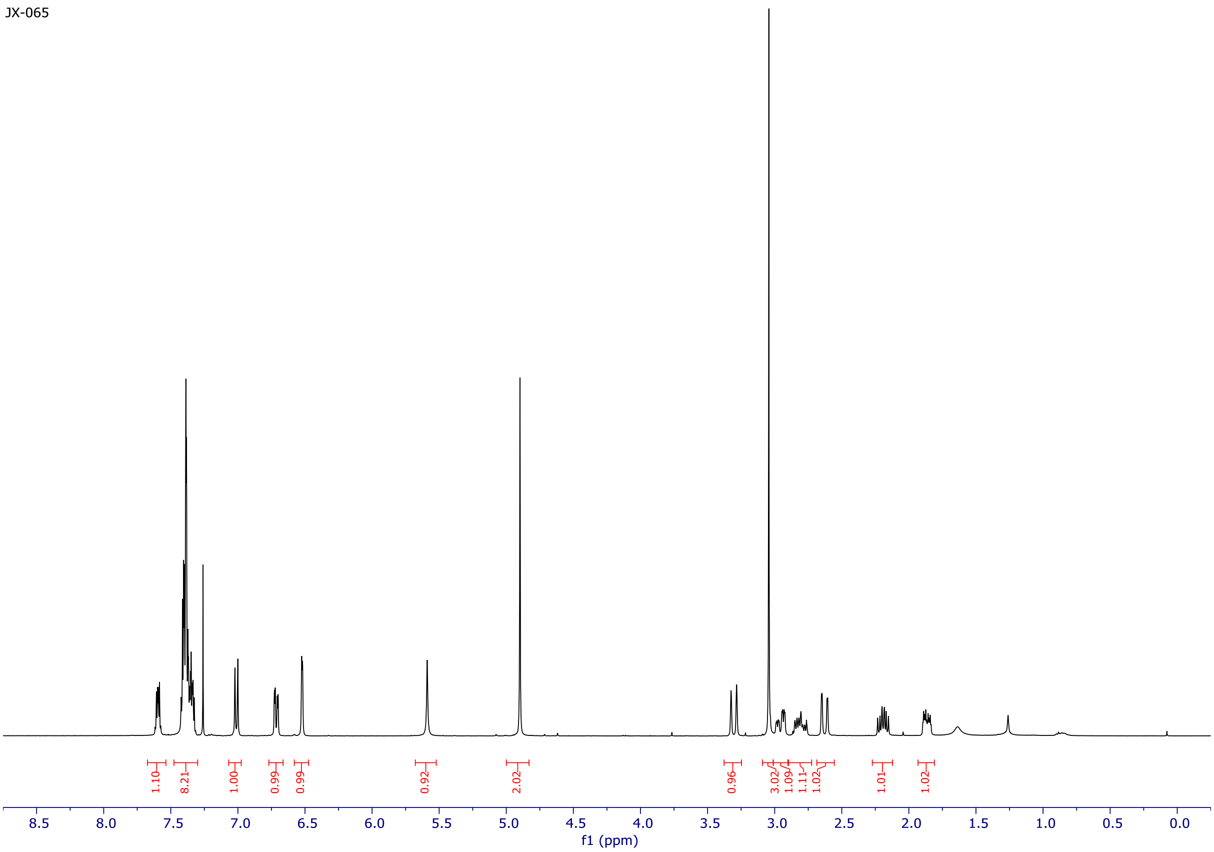
1.10
8.21
1.00
0.99
0.99
0.92
2.02
0.96
3.02
1.09
1.11
1.02
1.01
1.02
8.5
8.0
7.5
7.0
6.5
6.0
5.5
5.0
4.5
4.0
3.5
3.0
2.5
2.0
1.5
1.0
0.5
0.0
f1 (ppm)

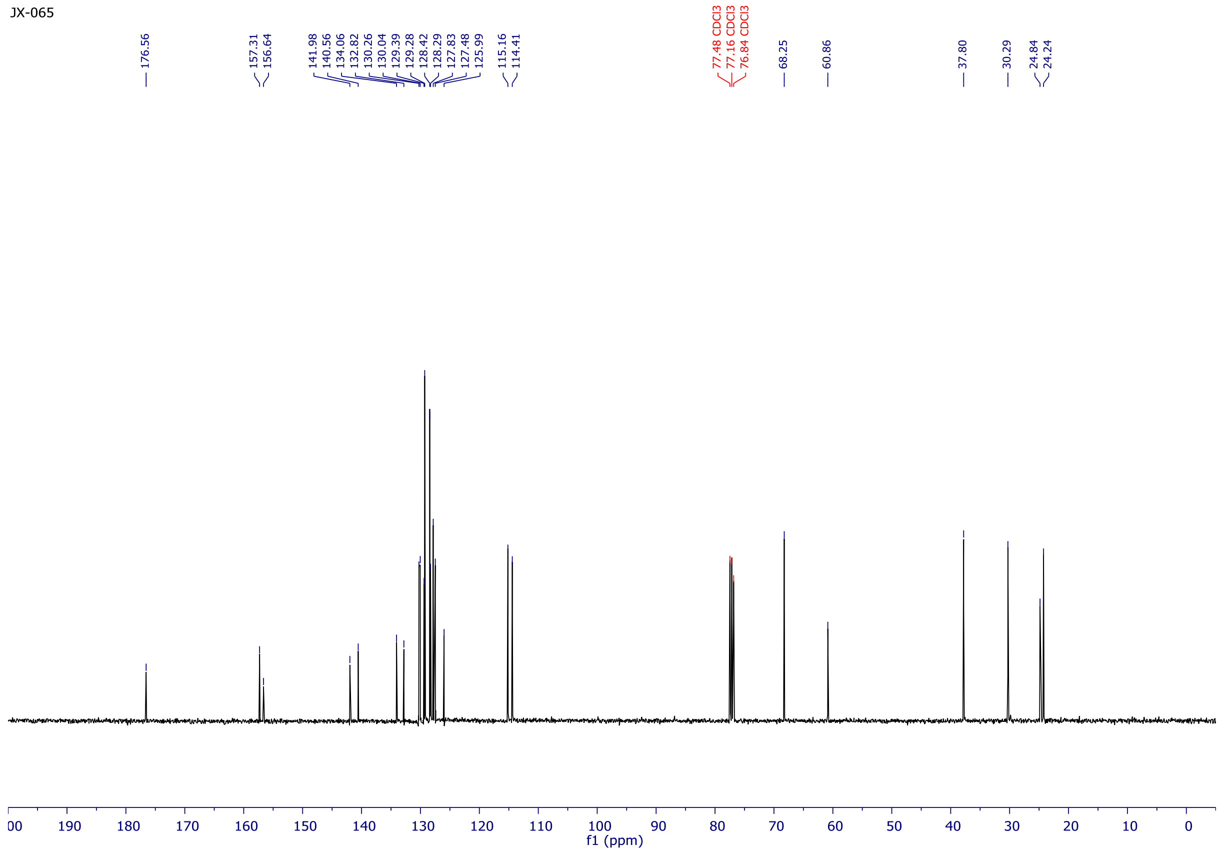
JX-065
176.56
157.31
156.64
141.98
140.56
134.06
132.82
130.26
130.04
129.39
129.28
128.42
128.29
127.83
127.48
125.99
115.16
114.41
77.48 CDCl3
77.16 CDCl3
76.84 CDCl3
68.25
60.86
37.80
30.29
24.84
24.24
f1 (ppm)

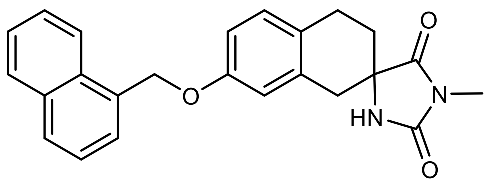

JX-070

1-Methyl-7'-(naphthalen-1-ylmethoxy)-3',4'-dihydro-1'H-spiro[imidazolidine-4,2'-naphthalene]-2,5-dione (JX-070): To a solution of **JX-059** (50 mg, 0.20 mmol, 1.0 equiv) in dry DMF (2.0 mL) was added Cs_2CO_3 (100 mg, 0.306 mmol, 1.5 equiv) in one portion at rt under argon and the mixture was continued to stir. After 10 min, 2-methylnaphthalene bromide (0.047 mg, 0.21 mmol, 1.05 equiv) was added and the reaction mixture was heated to 60 °C. After 15 h, the reaction was allowed to cool to rt, the mixture was diluted with EtOAc (100 mL), and the solution was washed with brine (3 × 25 mL). The organic layer was dried with $MgSO_4$, filtered, and concentrated under reduced pressure. The crude product was purified by flash column chromatography (CH_2Cl_2/MeOH 98:2) to yield **JX-070** (69 mg, 87%) as a white solid.

TLC (SiO_2; CH_2Cl_2/MeOH 98:2, UV, $KMnO_4$ stain): R_f = 0.42.

mp: 208.2-208.9 °C.

^{1}H-NMR (400 MHz, $CDCl_3$): δ (ppm) = 8.07 – 7.98 (m, 1H), 7.92 – 7.87 (m, 1H), 7.86 (d, *J* = 8.2 Hz, 1H), 7.58 (dd, *J* = 7.0, 1.2 Hz, 1H), 7.56 – 7.49 (m, 2H), 7.46 (dd, *J* = 8.2, 7.0 Hz, 1H), 7.08 (d, *J* = 8.4 Hz, 1H), 6.90 (dd, *J* = 8.4, 2.7 Hz, 1H), 6.75 (d, *J* = 2.6 Hz, 1H), 5.74 (s, 1H), 5.45 (s, 2H), 3.36 (d, *J* = 16.5 Hz, 1H), 3.05 (s, 3H), 2.99 (ddd, *J* = 17.3, 6.6, 2.8 Hz, 1H), 2.85 (ddd, *J* = 17.5, 12.0, 6.2 Hz, 1H), 2.70 (dd, *J* = 16.6, 2.4 Hz, 1H), 2.22 (ddd, *J* = 13.4, 12.0, 6.7 Hz, 1H), 1.89 (dddd, *J* = 13.3, 6.2, 2.6, 2.6 Hz, 1H).

^{13}C-NMR (101 MHz, $CDCl_3$): δ (ppm) = 176.57, 157.55, 156.69, 133.91, 133.02, 132.28, 131.61, 130.16, 129.20, 128.85, 126.71, 126.60, 126.28, 126.06, 125.43, 123.75, 115.31, 114.34, 68.85, 60.86, 37.86, 30.32, 24.83, 24.27.

IR (thin film): ʋ (cm^{-1}) = 3293, 2927, 1771, 1704, 1609, 1502, 1461, 1394, 1344, 1308, 1265, 1224, 1160, 1113, 1017, 999, 794, 755, 715, 636, 586.

HR-MS (ESI): Calcd for $C_{24}H_{23}N_2O_3$ $[M+H]^+$, 387.1703 *m/z*; found, 387.1697 *m/z.*

JX-070

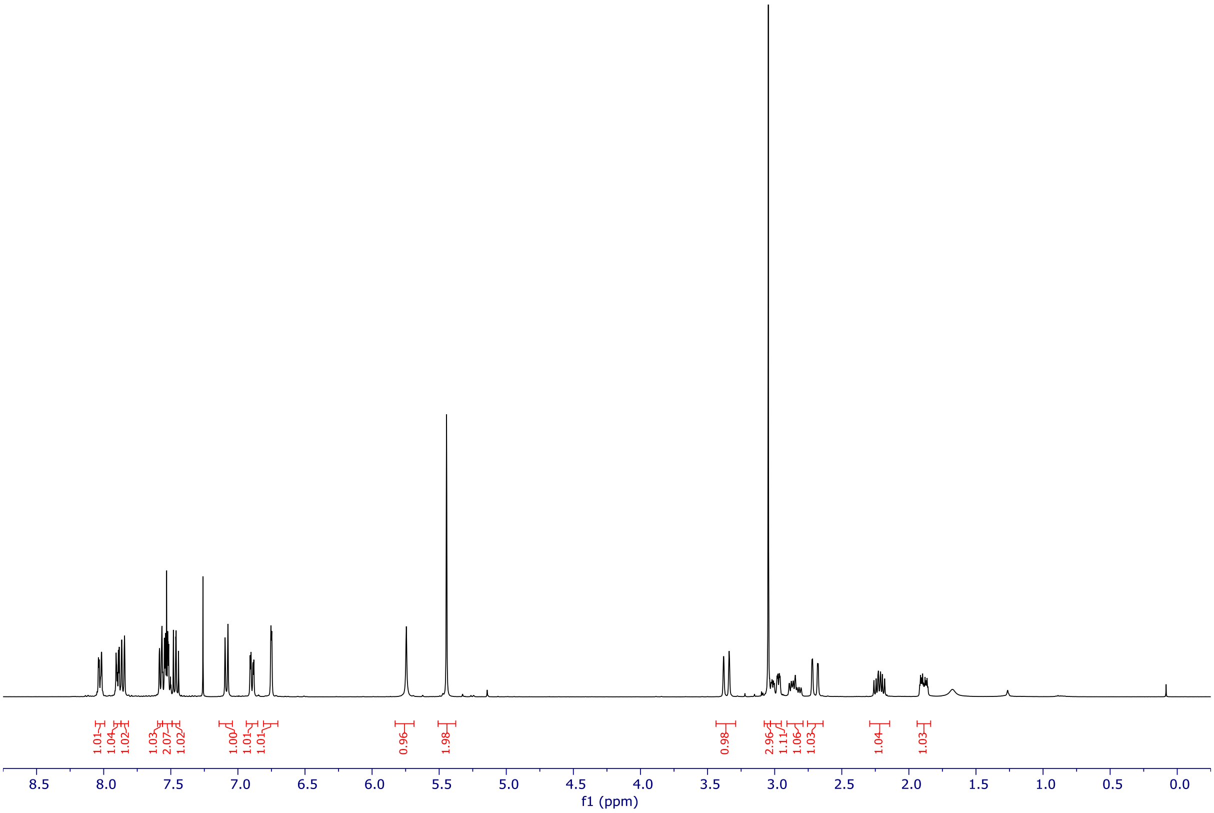

1.01
1.04
1.02
1.03
2.07
1.02
1.00
1.01
1.01
0.96
1.98
0.98
2.96
1.11
1.06
1.03
1.04
1.03
8.5
8.0
7.5
7.0
6.5
6.0
5.5
5.0
4.5
4.0
3.5
3.0
2.5
2.0
1.5
1.0
0.5
0.0
f1 (ppm)

JX-070

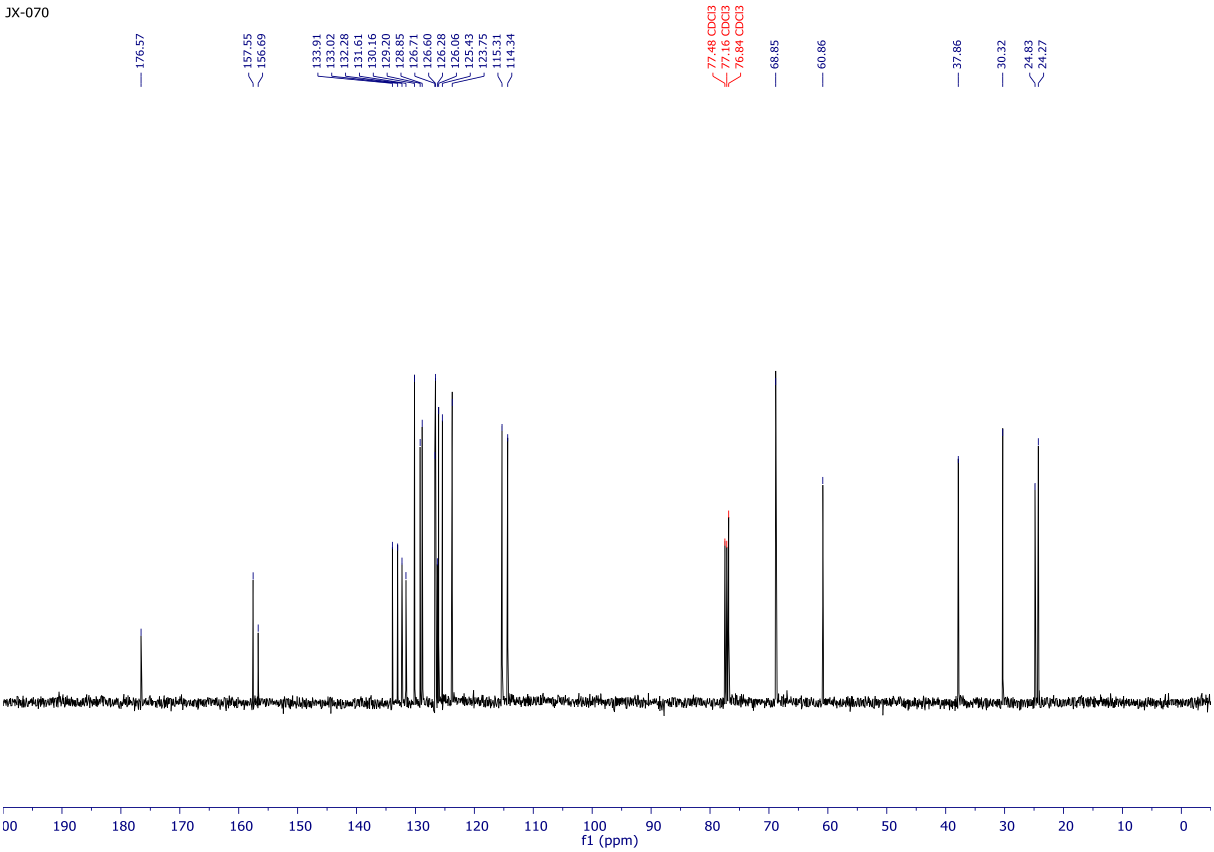
176.57
157.55
156.69
133.91
133.02
132.28
131.61
130.16
129.20
128.85
126.71
126.60
126.28
126.06
125.43
123.75
115.31
114.34
77.48 CDCl3
77.16 CDCl3
76.84 CDCl3
68.85
60.86
37.86
30.32
24.83
24.27
f1 (ppm)

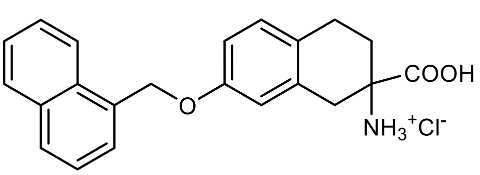

JX-075

2-Carboxy-7-(naphthalen-1-ylmethoxy)-1,2,3,4-tetrahydronaphthalen-2-aminium chloride (JX-075): A sealed microwave vial containing **JX-070** (21 mg, 0.053 mmol, 1.0 equiv) and $Ba(OH)_2{\cdot}8H_2O$ (84 mg, 0.27 mmol, 5.0 equiv) in H_2O/EtOH (2:1, 0.5 mL) was irradiated with microwaves for 2 h of at 150 °C. The pH was then adjusted to 1 using HCl (aq., 3 M) and the resulting mixture was extracted with EtOAc (3 × 25 mL). The combined organic phases were washed with brine (3 × 10 mL), dried with $MgSO_4$, filtered, and concentrated under reduced pressure. The crude product was purified by flash column chromatography ($CHCl_3$/MeOH/H_2O 70:30:5) to yield **JX-075** (16 mg, 0.045 mmol, 85%) as a white solid.

To ascertain that all of the material is present as the HCl-salt, **JX-075** was suspended in HCl in CPME (3 M, 1.5 mL) and stirred at rt under argon atmosphere. After the appearance of a precipitate, the mixture was filtered over celite, and the pad was washed with CPME. The remaining solid was washed down with MeOH and the filtrate was concentrated under reduced pressure to yield **JX-075** (17 mg, 0.044 mmol, 82%) as a white solid.

TLC (SiO_2; $CHCl_3$/MeOH/H_2O 70:30:5), UV, $KMnO_4$ stain): $R_f = 0.39$.

^{1}H-NMR (400 MHz, CD_3OD): δ (ppm) = 8.10 – 8.03 (m, 1H), 7.93 – 7.87 (m, 1H), 7.85 (d, *J* = 8.2 Hz, 1H), 7.59 (dd, *J* = 7.0, 1.1 Hz, 1H), 7.56 – 7.48 (m, 2H), 7.44 (dd, *J* = 8.3, 7.0 Hz, 1H), 7.12 (d, *J* = 8.5 Hz, 1H), 6.94 (dd, *J* = 8.5, 2.6 Hz, 1H), 6.87 (d, *J* = 2.6 Hz, 1H), 5.49 (s, 2H), 3.48 (d, *J* = 17.1 Hz, 1H), 3.01 (d, *J* = 16.7 Hz, 1H), 2.97 (ddd, *J* = 17.3, 6.4, 6.4 Hz, 1H), 2.84 (ddd, *J* = 16.9, 10.1, 6.0 Hz, 1H), 2.38 (ddd, *J* = 13.9, 10.0, 6.5 Hz, 1H), 2.16 (dddd, *J* = 13.9, 5.6, 5.2, 1.7 Hz, 1H).

^{13}C-NMR (101 MHz, CD_3OD): δ (ppm) = 173.62, 158.94, 135.27, 133.95, 132.95, 131.13, 129.94, 129.65, 127.68, 127.35, 127.33, 126.92, 126.26, 124.85, 116.00, 115.58, 69.69, 59.53, 36.43, 30.40, 24.59.

IR (solid): υ (cm^{-1}) = 3043, 2914, 2854, 1731, 1671, 1611, 1578, 1456, 1445, 1389,

1338, 1274, 1241, 1222, 1158, 1117, 1068, 1017, 1005, 919, 848, 771, 736, 699, 544, 532, 451, 444.

HR-MS (ESI): Calcd for $C_{22}H_{21}NNaO_3$ $[M+Na]^+$, 370.1414 *m/z*; found, 370.1421 *m/z*.

JX-075

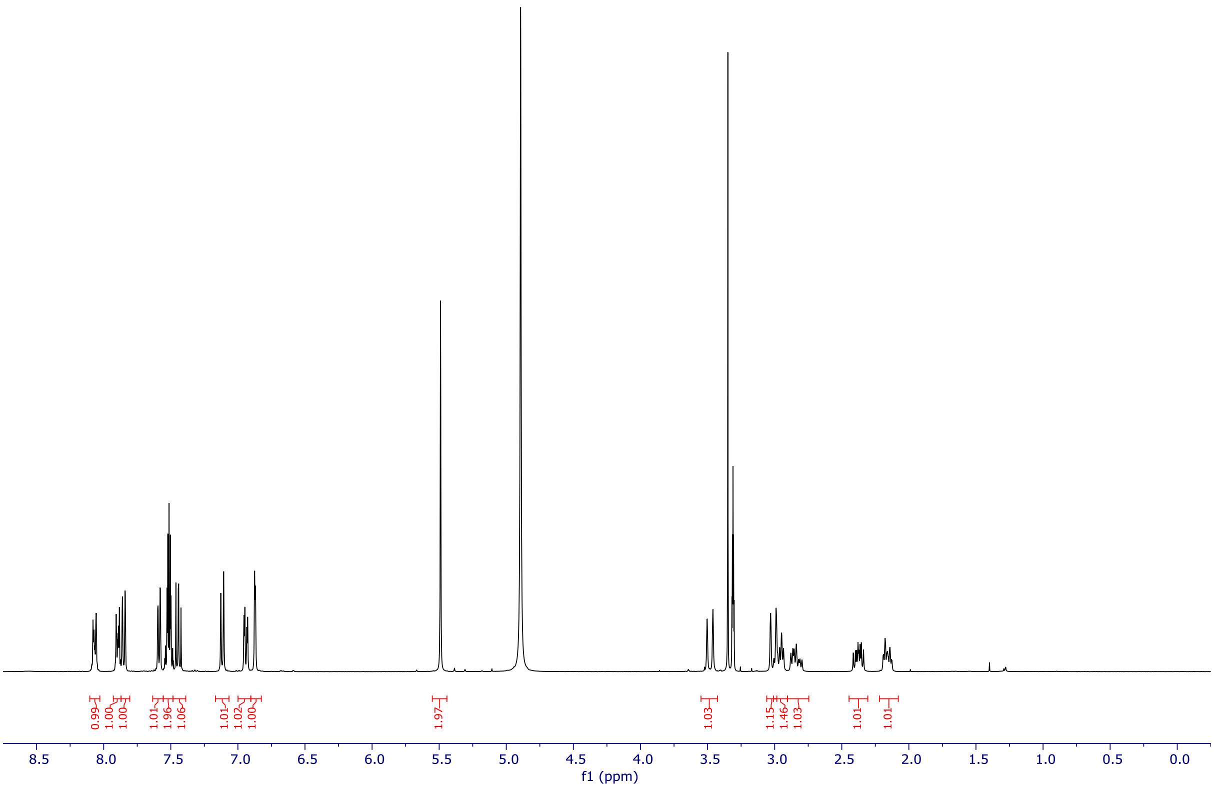

0.99
1.00
1.00
1.01
1.96
1.06
1.01
1.02
1.00
1.97
1.03
1.15
1.46
1.03
1.01
1.01
8.5
8.0
7.5
7.0
6.5
6.0
5.5
5.0
4.5
4.0
3.5
3.0
2.5
2.0
1.5
1.0
0.5
0.0
f1 (ppm)

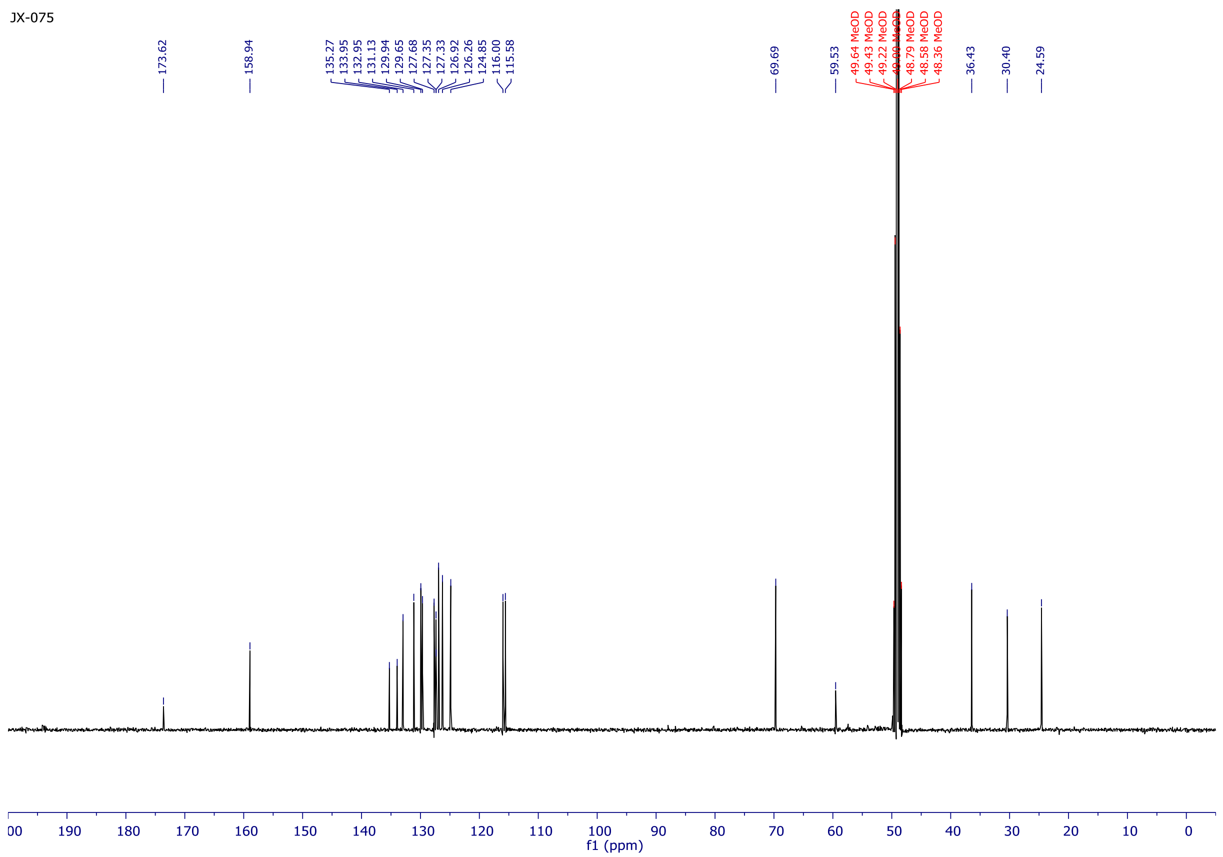

JX-075
173.62
158.94
135.27
133.95
132.95
131.13
129.94
129.65
127.68
127.35
127.33
126.92
126.26
124.85
116.00
115.58
69.69
59.53
49.64 MeOD
49.43 MeOD
49.22 MeOD
49.00 MeOD
48.79 MeOD
48.58 MeOD
48.36 MeOD
36.43
30.40
24.59
f1 (ppm)

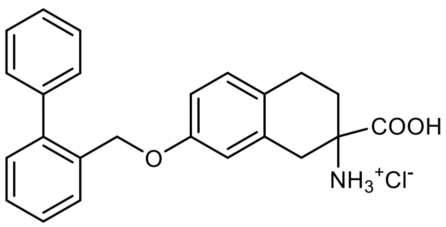

JX-078

7-([1,1'-Biphenyl]-2-ylmethoxy)-2-carboxy-1,2,3,4-tetrahydronaphthalen-2-aminium chloride (JX-078): In a microwave vial, **JX-065** (20 mg, 0.049 mmol, 1.0 equiv) and $Ba(OH)_2 \cdot 8H_2O$ (77 mg, 0.24 mmol, 5.0 equiv) was put in H_2O/EtOH (2:1, 0.5 mL) and the vial was sealed. After 2 h of irradiation at 150 °C in the microwave, the pH was adjusted to 1 using HCl (aq., 3 M). The resulting mixture was extracted with EtOAc (3 × 25 mL) and the combined organic phases were washed with brine (3 × 10 mL), dried with $MgSO_4$, filtered, and concentrated under reduced pressure. The crude product was purified by flash column chromatography ($CHCl_3$/MeOH/H_2O 70:30:5). to yield **JX-078** (13 mg, 0.034 mmol, 69%) as a white solid.

To ascertain that all of the material is present as the HCl-salt, **JX-078** was dissolved in HCl in CPME (3 M, 1 mL) and stirred at rt under argon atmosphere. After the appearance of a precipitate, the mixture was filtered over celite, and the pad was washed with CPME. The remaining solid was washed down with MeOH and concentrated under reduced pressure to yield **JX-078** (13 mg, 0.031 mmol, 63%) as a white solid.

TLC (SiO_2; $CHCl_3$/MeOH/H_2O 70:30:5), UV, $KMnO_4$ stain): R_f = 0.36.

^{1}**H-NMR** (400 MHz, CD_3OD): δ (ppm) = 7.61 – 7.50 (m, 1H), 7.43 – 7.32 (m, 7H), 7.32 – 7.27 (m, 1H), 7.05 (d, *J* = 8.4 Hz, 1H), 6.72 (dd, *J* = 8.4, 2.6 Hz, 1H), 6.61 (d, *J* = 2.6 Hz, 1H), 4.90 (s, 2H), 3.42 (d, *J* = 17.2 Hz, 1H), 3.02 – 2.87 (m, 1H), 2.93 (d, *J* = 17.6 Hz, 1H), 2.80 (ddd, *J* = 17.0, 10.2, 6.0 Hz, 1H), 2.36 (ddd, *J* = 14.0, 10.2, 6.5 Hz, 1H), 2.20 – 2.07 (m, 1H).

^{13}**C-NMR** (101 MHz, CD_3OD): δ (ppm) = 173.68, 158.68, 143.59, 142.00, 135.46, 132.83, 131.06, 130.68, 130.26, 129.28, 128.62, 128.40, 127.16, 115.61, 115.48, 69.23, 59.52, 36.39, 30.35, 24.52.

IR (solid): υ (cm^{-1}) = 3397, 3022, 2926, 1725, 1609, 1503, 1481, 1453, 1384, 1263, 1240, 1158, 1118, 1009, 950, 916, 875, 848, 811, 775, 748, 702, 616, 557, 542, 440, 414.

HR-MS (ESI): Calcd for $C_{24}H_{24}NO_3$ $[M+H]^+$, 374.1751 *m/z*; found, 374.1758 *m/z*.

JX-078

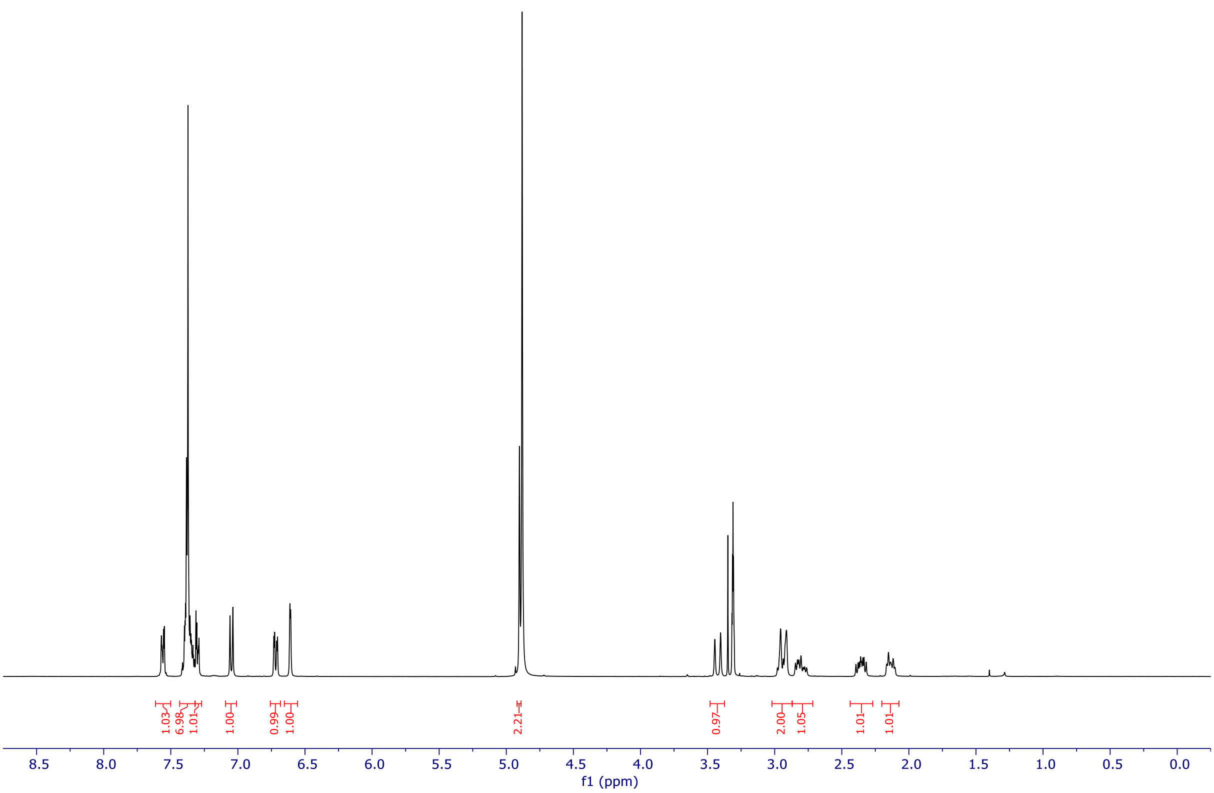

1.03
6.98
1.01
1.00
0.99
1.00
2.21
0.97
2.00
1.05
1.01
1.01
8.5 8.0 7.5 7.0 6.5 6.0 5.5 5.0 4.5 4.0 3.5 3.0 2.5 2.0 1.5 1.0 0.5 0.0
f1 (ppm)

JX-078

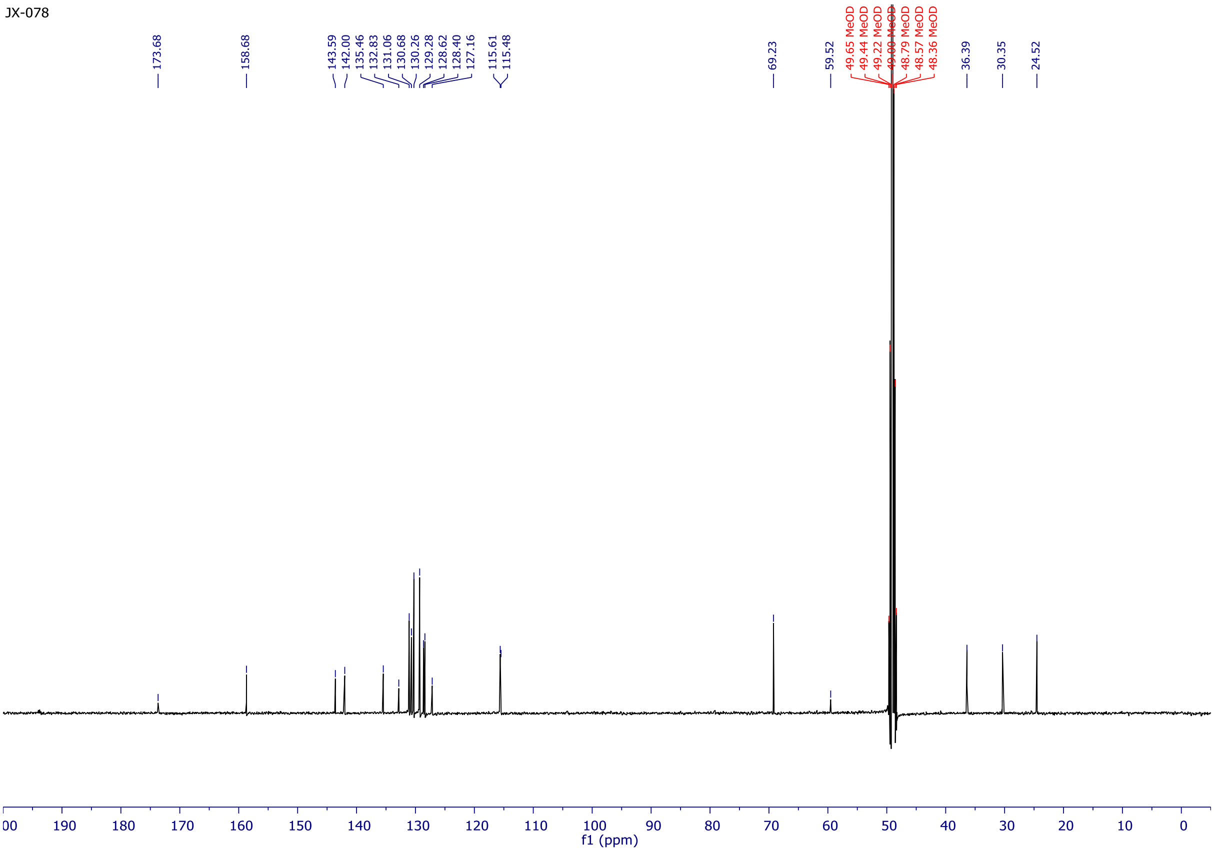

173.68
158.68
143.59
142.00
135.46
132.83
131.06
130.68
130.26
129.28
128.62
128.40
127.16
115.61
115.48
69.23
59.52
49.65 MeOD
49.44 MeOD
49.22 MeOD
49.00 MeOD
48.79 MeOD
48.57 MeOD
48.36 MeOD
36.39
30.35
24.52
190 180 170 160 150 140 130 120 110 100 90 80 70 60 50 40 30 20 10 0
f1 (ppm)

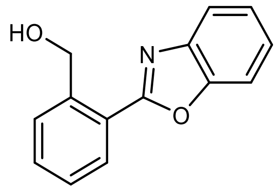

JX-107

(2-(Benzo[d]oxazol-2-yl)phenyl)methanol (JX-107): In a microwave vial, **2-chlorobenzoxazole** (0.10 mL, 0.88 mmol, 1.0 equiv), **2-(hydroxy-methyl)benzeneboronic acid** (200 mg, 1.32 mmol, 1.5 equiv), K_2CO_3 (243 mg, 1.76 mmol, 2.0 equiv), and $Pd(PPh_3)_4$ (51 mg, 0.044 mmol, 0.05 equiv) were dissolved in degassed 1,2-dimethoxyethane/H_2O (8:3, 13.0 mL) and the vial was sealed. After 30 min of microwave irradiation at 90 °C, the organic phase was separated from the aqueous phase, which was extracted with EtOAc (3 × 50 mL). The combined organic phases were washed with brine (3 × 25 mL), dried with $MgSO_4$, filtered, and concentrated under reduced pressure. The crude product was purified by flash column chromatography (CH_2Cl_2/MeOH 98:2) to yield **JX-107** (145 mg, 0.646 mmol, 73%) as a yellowish white solid.

TLC (SiO_2; hexane/EtOAc 7:3, UV, $KMnO_4$ stain): R_f = 0.39.

mp: 90.6-92.3 °C.

^{1}H-NMR (400 MHz, $CDCl_3$): δ (ppm) = 8.24 (ddd, *J* = 6.9, 1.4, 1.4 Hz, 1H), 7.81 – 7.75 (m, 1H), 7.65 – 7.59 (m, 1H), 7.55 – 7.45 (m, 3H), 7.43 – 7.36 (m, 2H), 6.13 (brs, 1H), 4.87 (s, 2H).

^{13}C-NMR (101 MHz, $CDCl_3$): δ (ppm) = 162.51, 150.28, 141.72, 141.38, 131.92, 131.12, 129.88, 128.38, 125.92, 125.83, 124.99, 120.14, 110.87, 64.81.

IR (thin film): υ (cm^{-1}) = 3386, 3064, 2954, 2870, 1614, 1548, 1486, 1475, 1454, 1442, 1343, 1301, 1270, 1244, 1203, 1192, 1126, 1108, 1055, 1024, 959, 924, 894, 836, 820, 798, 778, 759, 742, 725, 708, 607, 452, 427.

HR-MS (ESI): Calcd for $C_{14}H_{11}NNaO_2$ $[M+Na]^+$, 248.0682 *m/z*; found, 248.0685 *m/z*.

JX-107

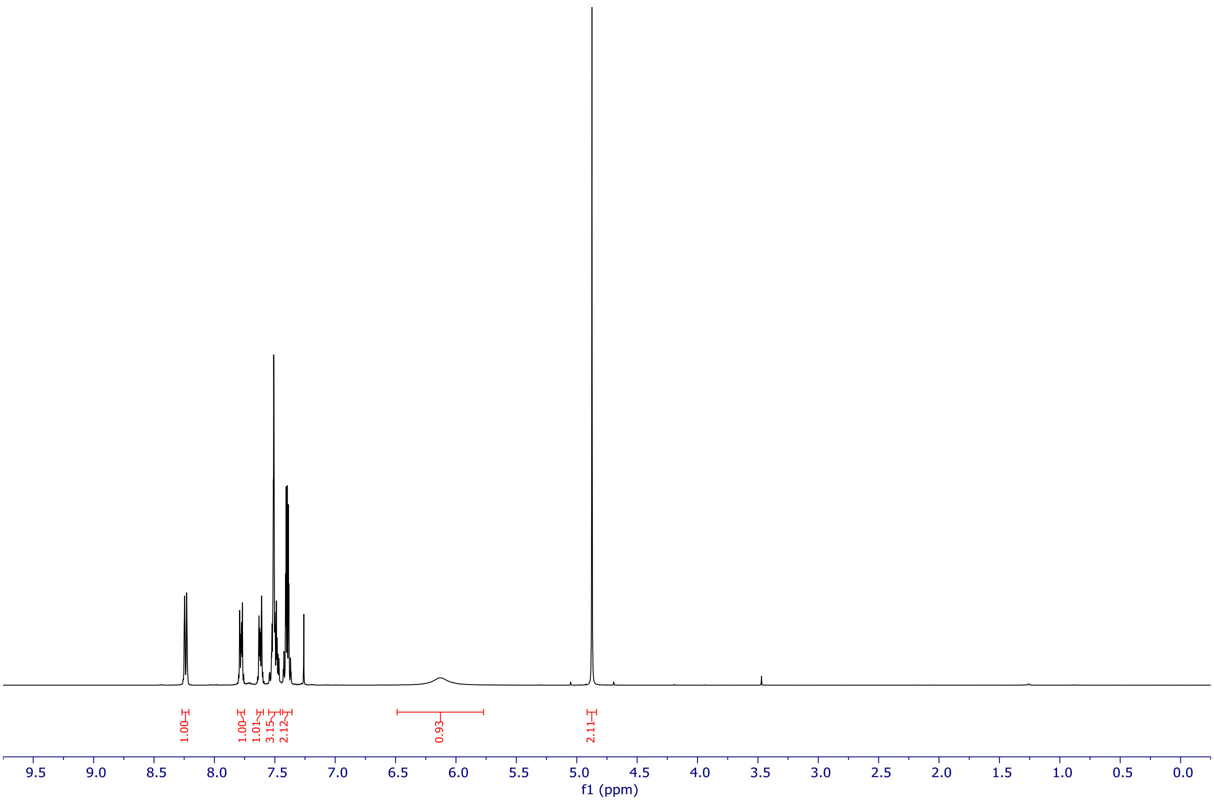
1.00
1.00
1.01
3.15
2.12
0.93
2.11
9.5 9.0 8.5 8.0 7.5 7.0 6.5 6.0 5.5 5.0 4.5 4.0 3.5 3.0 2.5 2.0 1.5 1.0 0.5 0.0
f1 (ppm)

JX-107

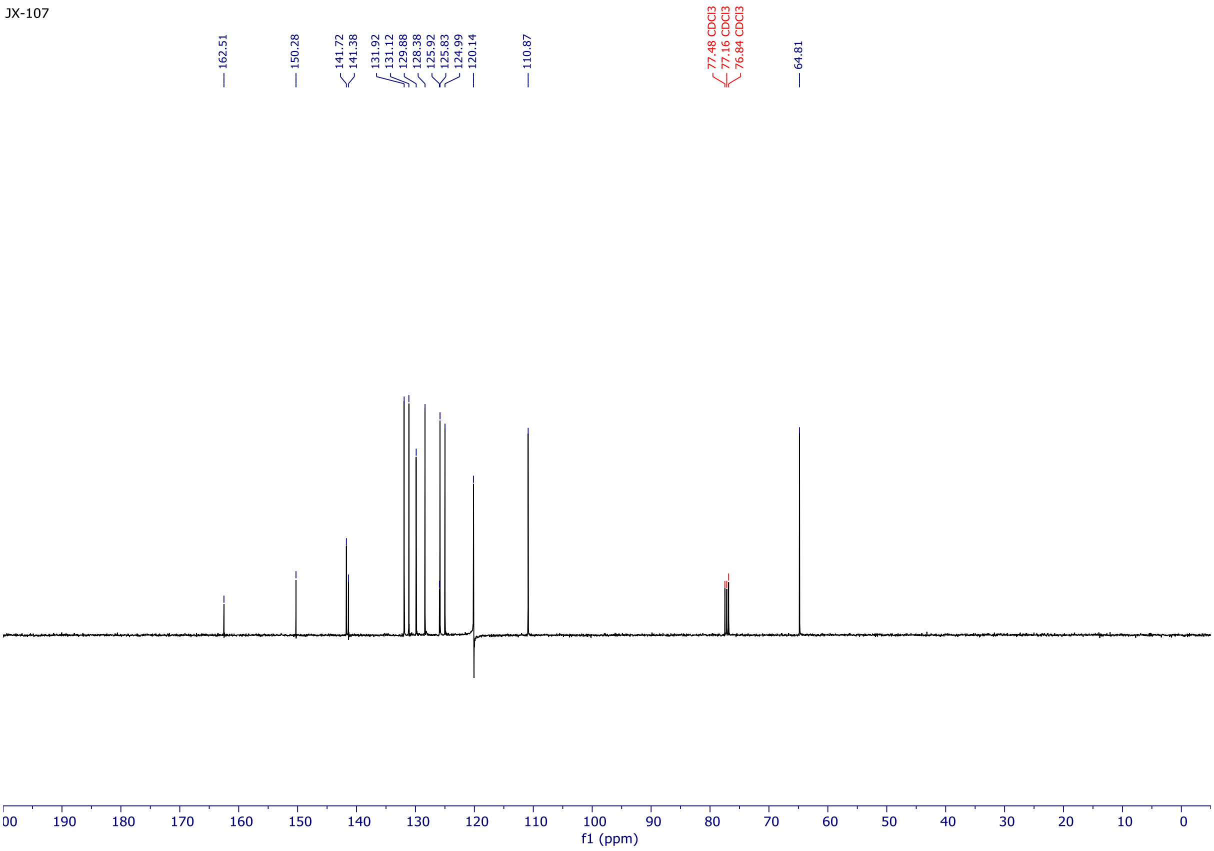

162.51
150.28
141.72
141.38
131.92
131.12
129.88
128.38
125.92
125.83
124.99
120.14
110.87
77.48 CDCl3
77.16 CDCl3
76.84 CDCl3
64.81
00
190
180
170
160
150
140
130
120
110
100
90
80
70
60
50
40
30
20
10
0
f1 (ppm)

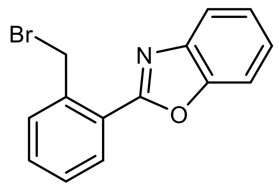

JX-108

2-(2-(Bromomethyl)phenyl)benzo[d]oxazole (JX-108): To a suspension of **JX-107** (78 mg, 0.35 mmol, 1.0 equiv) in dry CH_2Cl_2 (3.5 mL) were added CBr_4 (172 mg, 0.519 mmol, 1.5 equiv) and PPh_3 (136 mg, 0.519 mmol, 1.5 equiv) at 0 °C under argon. After 1 h, the reaction was quenched by adding H_2O. The mixture was extracted with CH_2Cl_2 (3x25 mL) and the combined organi extracts were dried with $MgSO_4$, filtered, and concentrated under reduced pressure. The crude product was purified by flash column chromatography (hexane/EtOAc 9:1) to yield **JX-108** (82 mg, 0.29 mmol, 82%) as a beige white solid.

TLC (SiO_2; hexane/EtOAc 9:1, UV, $KMnO_4$ stain): R_f = 0.35.

mp: 93.6-94.4 °C.

^{1}H-NMR (400 MHz, $CDCl_3$): δ (ppm) = 8.23 (dd, J = 7.4, 1.9 Hz, 1H), 7.90 – 7.79 (m, 1H), 7.66 – 7.55 (m, 2H), 7.54 – 7.43 (m, 2H), 7.43 – 7.35 (m, 2H), 5.34 (s, 2H).

^{13}C-NMR (101 MHz, $CDCl_3$): δ (ppm) = 161.66, 150.28, 142.23, 138.09, 132.18, 131.47, 130.37, 128.90, 125.75, 125.59, 124.73, 120.56, 110.71, 32.35.

IR (thin film): υ (cm^{-1}) = 3059, 1613, 1550, 1491, 1474, 1453, 1443, 1342, 1311, 1285, 1268, 1242, 1223, 1208, 1195, 1112, 1031, 1003, 926, 894, 880, 831, 795, 777, 760, 743, 702, 626, 603, 580, 531, 504.

HR-MS (ESI): Calcd for $C_{14}H_{11}BrNO$ $[M+H]^+$, 288.0019 *m/z*; found, 288.0019 *m/z*.

JX-108

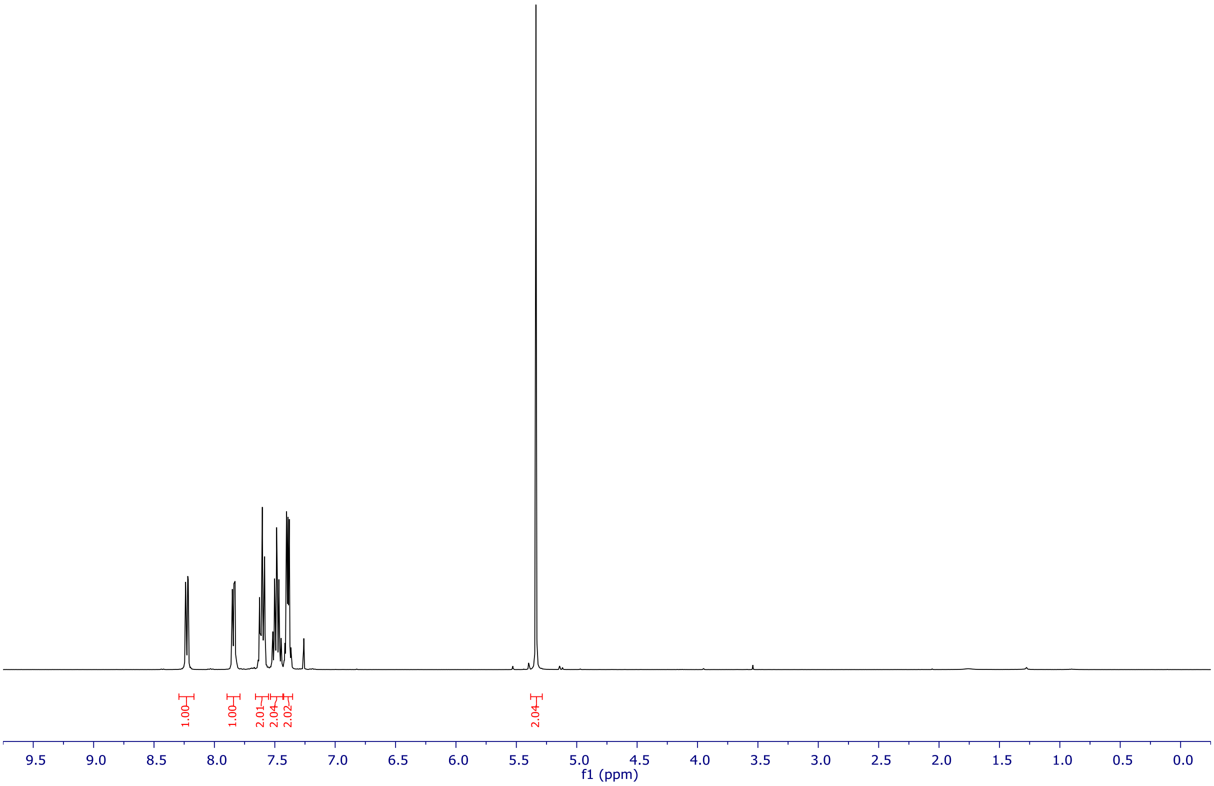

1.00
1.00
2.01
2.04
2.02
2.04
9.5
9.0
8.5
8.0
7.5
7.0
6.5
6.0
5.5
5.0
4.5
4.0
3.5
3.0
2.5
2.0
1.5
1.0
0.5
0.0
f1 (ppm)

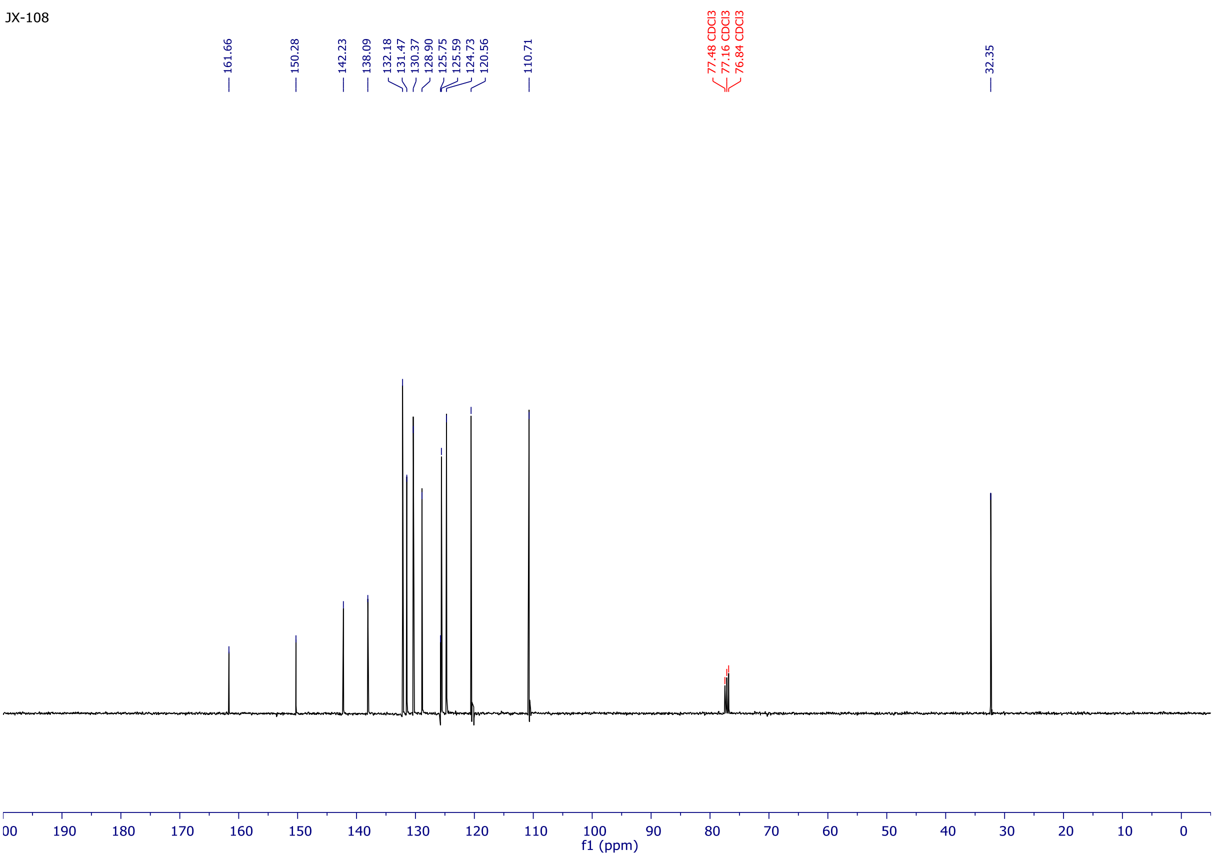

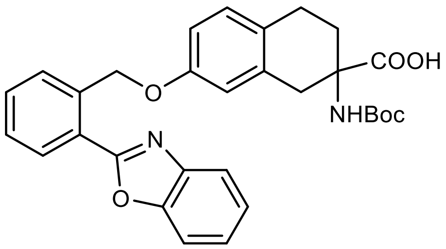

JX-110

7-((2-(Benzo[d]oxazol-2-yl)benzyl)oxy)-2-((tert-butoxycarbonyl)amino)-1,2,3,4-tetrahydronaphthalene-2-carboxylic acid (JX-110): To a stirred solution of **KH-142** (15 mg, 0.048 mmol, 1.0 equiv) in dry THF (0.5 mL) was added NaH (1 mg, 0.06 mmol, 1.2 equiv) , followed by **JX-108** (17 mg, 0.057 mmol, 1.2 equiv) at rt under argon. After 16 h, additional NaH (1 mg, 0.06 mmol, 1.2 equiv) was added and the reaction mixture was left stirring for 8 h. The reaction mixture was diluted with H_2O (1.5 mL) and after 17 h heated to 50 °C with additional NaOH (spatula tip). After 3 h at 50 °C, the reaction mixture was extracted with EtOAc (3 × 25 mL) and the combined organic phases were washed with brine (3 × 10 mL), dried with $MgSO_4$,

filtered, and concentrated under reduced pressure. The crude product was purified by flash column chromatography (CH_2Cl_2/MeOH 93:7) to yield **JX-110** (2 mg, 7%) as a white solid together with unreacted methyl ester.

The unreacted methyl ester and LiOH·H_2O (20 mg, 0.48 mmol, 10.0 equiv) were dissolved in THF/H_2O (1:1, 1.0 mL) and stirred at rt. After 6 h, the reaction mixture was heated to 60 °C. After 17 h, additional LiOH·H_2O (20 mg, 0.48 mmol, 10.0 equiv) was added. After 74 h, the reaction mixture was extracted with EtOAc (3 × 25 mL) and the combined organic phases were washed with brine (3 × 10 mL), dried with $MgSO_4$, filtered, and concentrated under reduced pressure. The crude product was purified by flash column chromatography (CH_2Cl_2/MeOH 93:7 → 95:5) to yield **JX-110** (21 mg, 85%).

TLC (SiO_2; CH_2Cl_2/MeOH 95:5, UV, $KMnO_4$ stain): R_f = 0.18.

mp: 183.9-186.5 °C.

^{1}H-NMR (400 MHz, $(CD_3)_2SO$): δ (ppm) = 12.36 (brs, 1H), 8.23 (dd, *J* = 7.8, 1.5 Hz, 1H), 7.89 – 7.79 (m, 1H), 7.80 – 7.72 (m, 2H), 7.65 (ddd, *J* = 7.5, 1.5 Hz, 1H), 7.57 (ddd, *J* = 7.6, 1.4 Hz, 1H), 7.44 (pd, *J* = 7.4, 1.4 Hz, 2H), 7.03 (s, 1H), 6.96 (d, *J* = 8.5 Hz, 1H), 6.72 (dd, *J* = 8.3, 2.7 Hz, 1H), 6.68 (d, *J* = 2.7 Hz, 1H), 5.66 (s, 2H), 3.11 (d, *J* = 16.7 Hz, 1H), 2.96 (d, *J* = 16.9 Hz, 1H), 2.78 – 2.65 (m, 1H), 2.60 (ddd, *J* = 16.6, 5.3 Hz, 1H), 2.30 – 2.13 (m, 1H), 1.93 – 1.79 (m, 1H), 1.34 (s, 9H).

^{13}C-NMR (101 MHz, $(CD_3)_2SO$): δ (ppm) = 175.71, 161.59, 156.24, 155.06, 149.72, 141.45, 137.71, 135.40, 131.58, 129.55, 129.13, 128.15, 128.06, 127.48, 125.73, 124.82, 124.17, 120.04, 115.11, 112.58, 110.90, 77.86, 67.82, 56.93, 36.62, 28.99, 28.18, 24.15.

IR (thin film): υ (cm^{-1}) = 3426, 3325, 3061, 2976, 2926, 1712, 1613, 1580, 1551, 1501, 1454, 1392, 1367, 1243, 1162, 1127, 1109, 1065, 1028, 924, 894, 853, 805, 778, 760, 743, 704.

HR-MS (ESI): Calcd for $C_{30}H_{30}N_2NaO_6$ $[M+Na]^+$, 537.1996 *m/z*; found, 537.1999 *m/z*.

JX-110

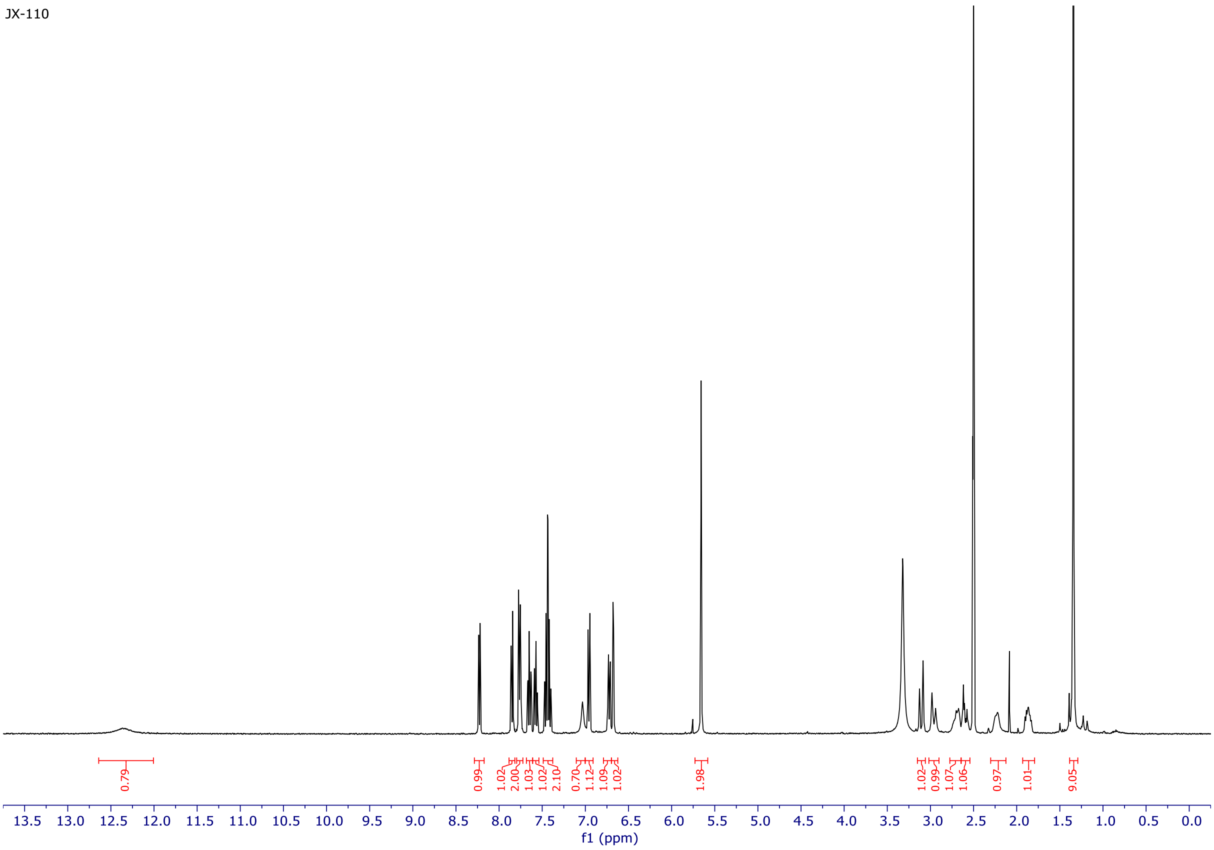
0.79
0.99
1.02
2.00
1.03
1.02
2.10
0.70
1.12
1.09
1.02
1.98
1.02
0.99
1.07
1.06
0.97
1.01
9.05
13.5 13.0 12.5 12.0 11.5 11.0 10.5 10.0 9.5 9.0 8.5 8.0 7.5 7.0 6.5 6.0 5.5 5.0 4.5 4.0 3.5 3.0 2.5 2.0 1.5 1.0 0.5 0.0
f1 (ppm)

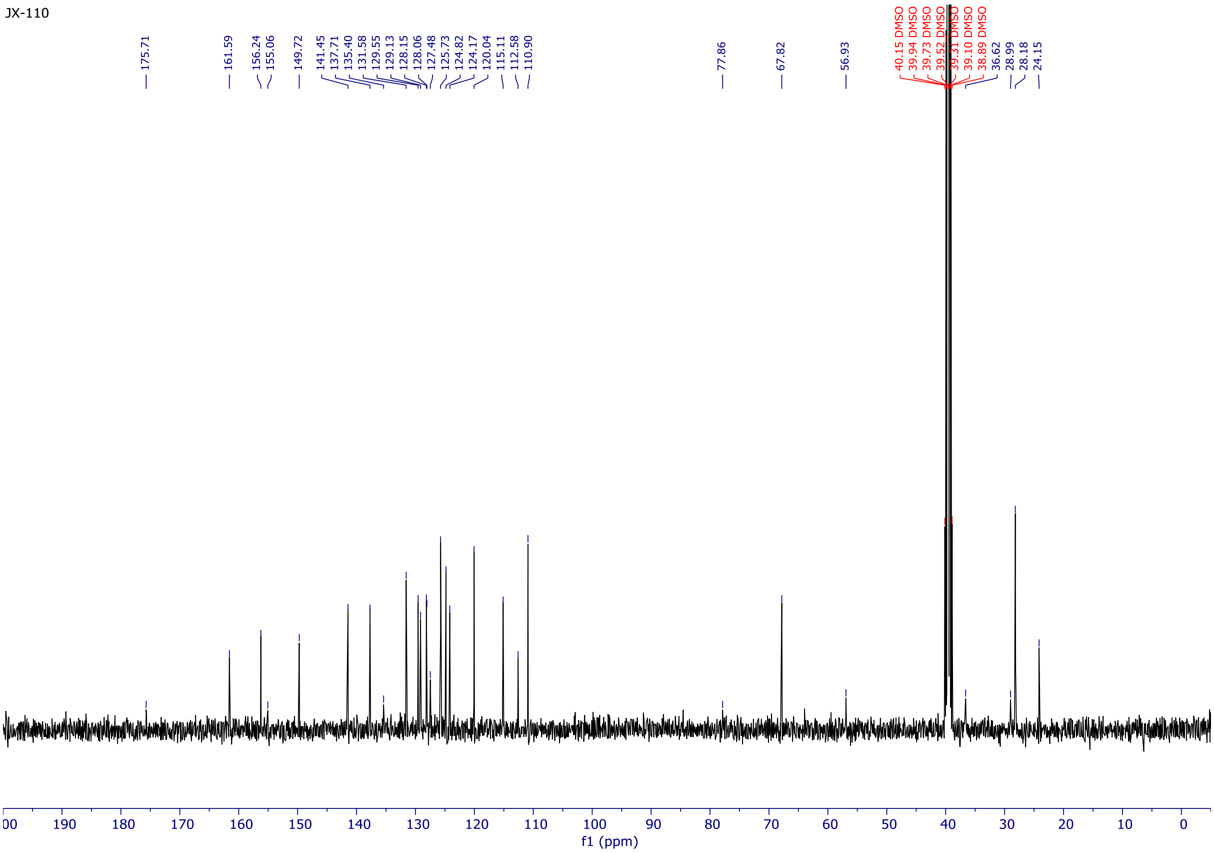

JX-110
175.71
161.59
156.24
155.06
149.72
141.45
137.71
135.40
131.58
129.55
129.13
128.15
128.06
127.48
125.73
124.82
124.17
120.04
115.11
112.58
110.90
77.86
67.82
56.93
40.15 DMSO
39.94 DMSO
39.73 DMSO
39.52 DMSO
39.31 DMSO
39.10 DMSO
38.89 DMSO
36.62
28.99
28.18
24.15
f1 (ppm)

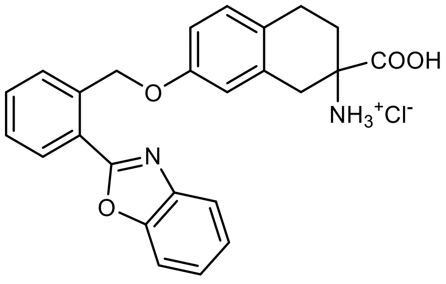

JX-119

7-((2-(Benzo[d]oxazol-2-yl)benzyl)oxy)-2-carboxy-1,2,3,4-tetrahydronaphthalen-2-aminium chloride (JX-119): JX-110 (6 mg, 0.01 mmol, 1.0 equiv) was dissolved in HCl (3 M in CPME, 1.0 mL) and stirred at rt under argon. After 16 h a precipitate was formed, which was collected by filtration using a Pasteur pipette equipped with cotton and a layer of celite. The residue was thoroughly washed with CPME and then flushed down using MeOH. The solvent was evaporated under reduced pressure to yield **JX-119** (4 mg, 0.01 mmol, 78%) as a beige solid.

TLC (SiO_2; $CHCl_3$/MeOH/H_2O 70:30:5), UV, $KMnO_4$ stain): R_f = 0.41.

mp: 234-238 °C (burned/decomposition).

^{1}H-NMR (400 MHz, CD_3OD): δ (ppm) = 8.24 (dd, *J* = 7.7, 1.5 Hz, 1H), 7.79 (dd, *J* = 7.7, 1.0 Hz, 1H), 7.76 (ddd, *J* = 5.9, 2.3, 0.5 Hz, 1H), 7.65 – 7.61 (m, 1H), 7.60 (ddd, *J* = 7.9, 7.5, 1.5 Hz, 1H), 7.54 (ddd, *J* = 7.6, 7.4, 1.5 Hz, 1H), 7.46 – 7.37 (m, 2H), 7.10 (d, *J* = 8.5 Hz, 1H), 6.86 (dd, *J* = 8.5, 2.7 Hz, 1H), 6.75 (d, *J* = 2.6 Hz, 1H), 5.69 (s, 2H), 3.44 (d, *J* = 17.3 Hz, 1H), 3.02 – 2.91 (m, 1H), 2.94 (d, *J* = 16.6 Hz, 1H), 2.82 (ddd, *J* = 17.0, 10.1, 5.9 Hz, 1H), 2.38 (ddd, *J* = 14.0, 10.1, 6.5 Hz, 1H), 2.14 (dddd, *J* = 13.8, 6.1, 4.9, 1.6 Hz, 1H).

^{13}C-NMR (101 MHz, CD_3OD): δ (ppm) = 173.58, 163.74, 158.90, 151.64, 143.02, 139.16, 132.88, 132.56, 131.17, 130.87, 129.49, 129.15, 127.33, 126.84, 126.02, 125.92, 120.96, 116.02, 115.54, 111.71, 69.69, 59.49, 36.40, 30.37, 24.54.

IR (solid): υ (cm^{-1}) = 3411, 2925, 1728, 1614, 1582, 1548, 1498, 1455, 1379, 1315, 1273, 1241, 1160, 1130, 1056, 1030, 920, 874, 849, 803, 759, 751, 721, 537, 484, 452, 415.

HR-MS (ESI): Calcd for $C_{25}H_{23}N_2O_4$ $[M+H]^+$, 415.1652 *m/z*; found, 415.1656 *m/z*. Calcd for $C_{25}H_{21}N_2O_4$ $[M-H]^-$, 413.1507 *m/z*; found, 413.1514 *m/z*.

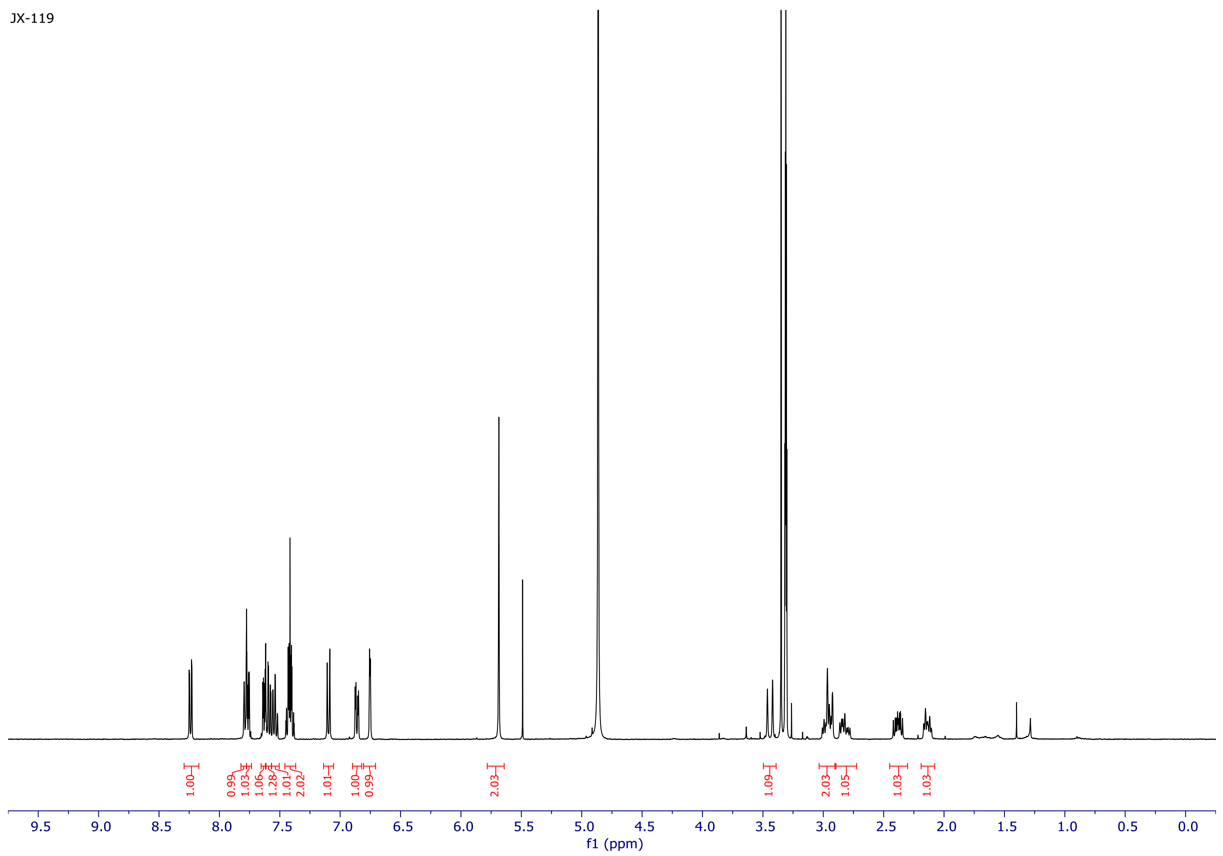
JX-119
1.00
0.99
1.03
1.06
1.28
1.01
2.02
1.01
1.00
0.99
2.03
1.09
2.03
1.05
1.03
1.03
9.5 9.0 8.5 8.0 7.5 7.0 6.5 6.0 5.5 5.0 4.5 4.0 3.5 3.0 2.5 2.0 1.5 1.0 0.5 0.0
f1 (ppm)

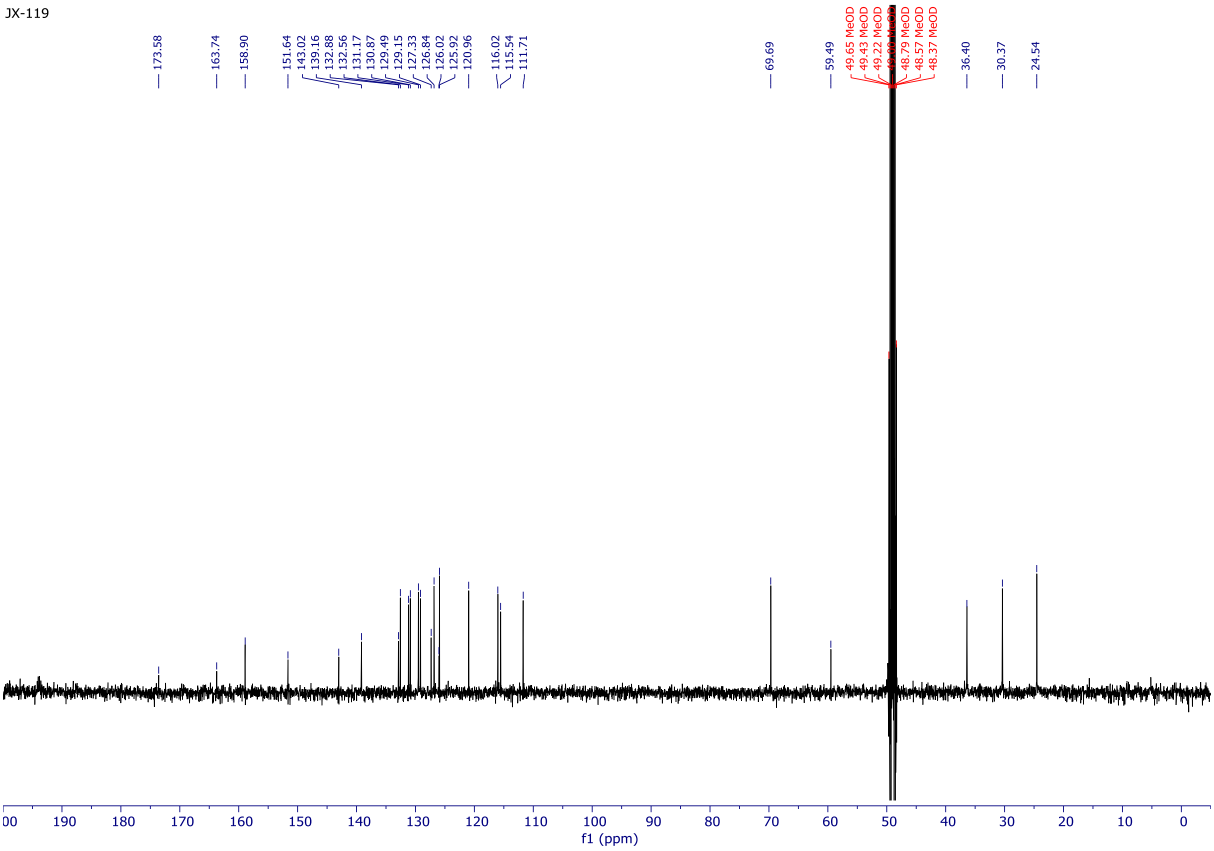

Inhibition of $[^3H]$-l-leucine uptake

HT-29 ells were seeded at 60% confluency in a 96-well plate using complete culture medium and cultured until confluent. Cells were washed three times with 37 °C pre-warmed Na^+-free Hank's balanced salt solution (HBBS) containing 125 mM choline-Cl, 25 mM HEPES, 4.8 mM KCl, 1.2 mM $MgSO_4$, 1.2 mM KH_2PO_4, 1.3 mM $CaCl_2$ and 5.6 mM glucose (pH 7.4) and further incubated in the same buffer at 37 °C for 7 min. L-leucine uptake was measured for 3 min at 37 °C in the same buffer containing 30 µM L-$[^3H]$leucine (100 Ci/mmol) and different concentrations of test compounds. Uptake was terminated by removing the solution followed by three washings with ice-cold Na^+-free HBBS. Cells were lysed and mixed with Microscint20 (Perkin-Elmer Life Sciences). The radioactivity was measured with a scintillation counter (TopCount NXT, Perkin-Elmer Life Sciences). Dose-response curves for cpds. **JX-075**, **JX-078**, and **JX-119** are depicted below:

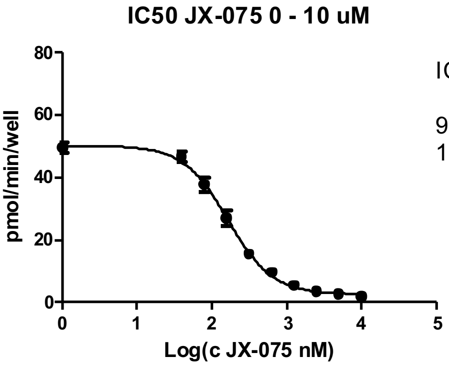

IC50 = 165 nM

95% confidence intervall
151 nM to 180 nM

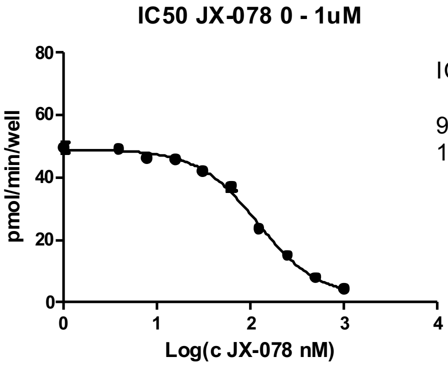

IC50 = 121 nM

95% confidence intervall
108 nM to 137 nM

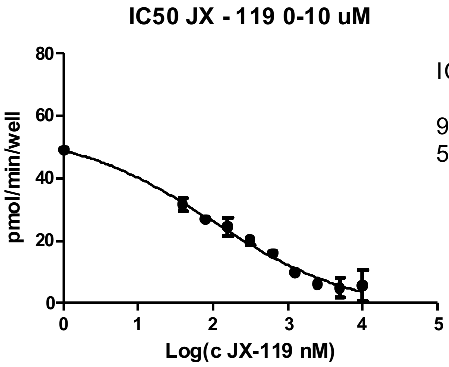

IC50 = 98 nM

95% confidence intervall
56 nM to 170 nM

Induction of [^{3}H]-l-leucine efflux (assessment of substrate properties)

The same protocol as for the assessment of uptake inhibition was used with the following differences after the initial washing and starvation step. Cells were preloaded for 5 min at 37 °C in the Na^{+}-free HBBS containing 30 μM L-[^{3}H]leucine (100 Ci/mmol). After washing three times with Na^{+}-free HBBS (4 °C), efflux of radioactivity was induced by incubation in the presence or absence of indicated concentrations of test compounds for 10 min at 37 °C. The medium was then collected and its radioactivity was counted. The cells were washed three times with ice-cold Na^{+}-free HBBS. Cells were lysed and mixed with Microscint20 (Perkin-Elmer Life Sciences). The radioactivity was measured with a scintillation counter (TopCount NXT, Perkin-Elmer Life Sciences). The L-[^{3}H]leucine efflux values were expressed as percentage radioactivity (radioactivity of medium)/(radioactivity of the medium + radioactivity of the cells). As shown below, **JX-075**, **JX-078**, and **JX-119** did not induce measurable leucine efflux at a concentration of up to 100 μM. The same result was obtained with the known LAT-1 inhibitor **JPH-203**. Based on these data, none of the compouns is a substrate for LAT-1.

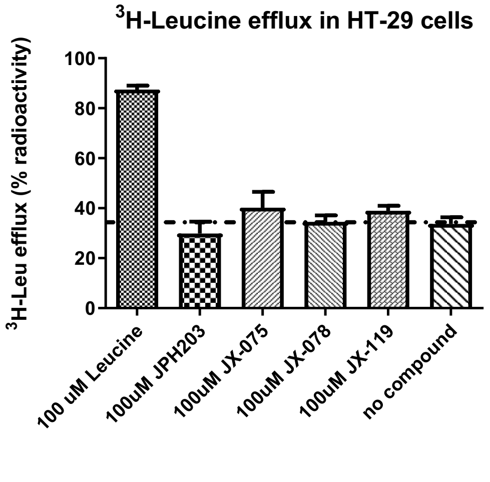

References

Synthesis of JX-075, JX-078, and JX-119

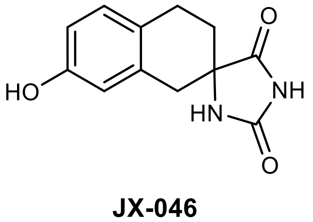

JX-046

7'-Hydroxy-3',4'-dihydro-1'H-spiro[imidazolidine-4,2'-naphthalene]-2,5-dione (JX-046): To a suspension of 7-hydroxy-2-tetralone (131 mg, 0.809 mmol, 1.0 equiv) in EtOH/H_2O (4:1, 2.6 mL) in a microwave vial were added $(NH_4)_2CO_3$ (700 mg, 7.28 mmol, 9.0 equiv) and KCN (79 mg, 1.2 mmol, 1.5 equiv) and the vial was sealed. After 30 min of microwave irradiation at 80 °C, the reaction mixture was diluted with H_2O, the solution was extracted with EtOAc (3 × 200 mL) and the combined organic phases were washed with brine (3 × 100 mL). The organic layer was dried over $MgSO_4$, filtered, and concentrated under reduced pressure to yield crude **JX-046** (172 mg, 0.742 mmol, 92%) as a beige brown solid. This material was used in the following step without purification.

TLC (SiO_2; CH_2Cl_2/MeOH 90:10, UV, $KMnO_4$ stain): R_f = 0.34.

^{1}H-NMR (400 MHz, CD_3OD): δ (ppm) = 6.96 (d, *J* = 8.2 Hz, 1H), 6.61 (dd, *J* = 8.3, 2.6 Hz, 1H), 6.53 (d, *J* = 2.6 Hz, 1H), 3.20 (d, *J* = 16.6 Hz, 1H), 2.94 – 2.84 (m, 2H), 2.72 (dd, *J* = 16.8, 2.0 Hz, 1H), 2.08 (ddd, *J* = 13.2, 9.4, 9.4 Hz, 1H), 1.89 (dddd, *J* = 13.2, 4.7, 4.6, 2.1 Hz, 1H).

^{13}C-NMR (101 MHz, CD_3OD): δ (ppm) = 180.63, 161.17, 156.75, 134.36, 130.82, 126.52, 116.24, 115.12, 63.30, 38.33, 31.81, 25.26.

HR-MS (ESI): Calcd for $C_{12}H_{13}N_2O_3$ $[M+H]^+$, 233.0921 *m/z*; found 233.0921 *m/z*.

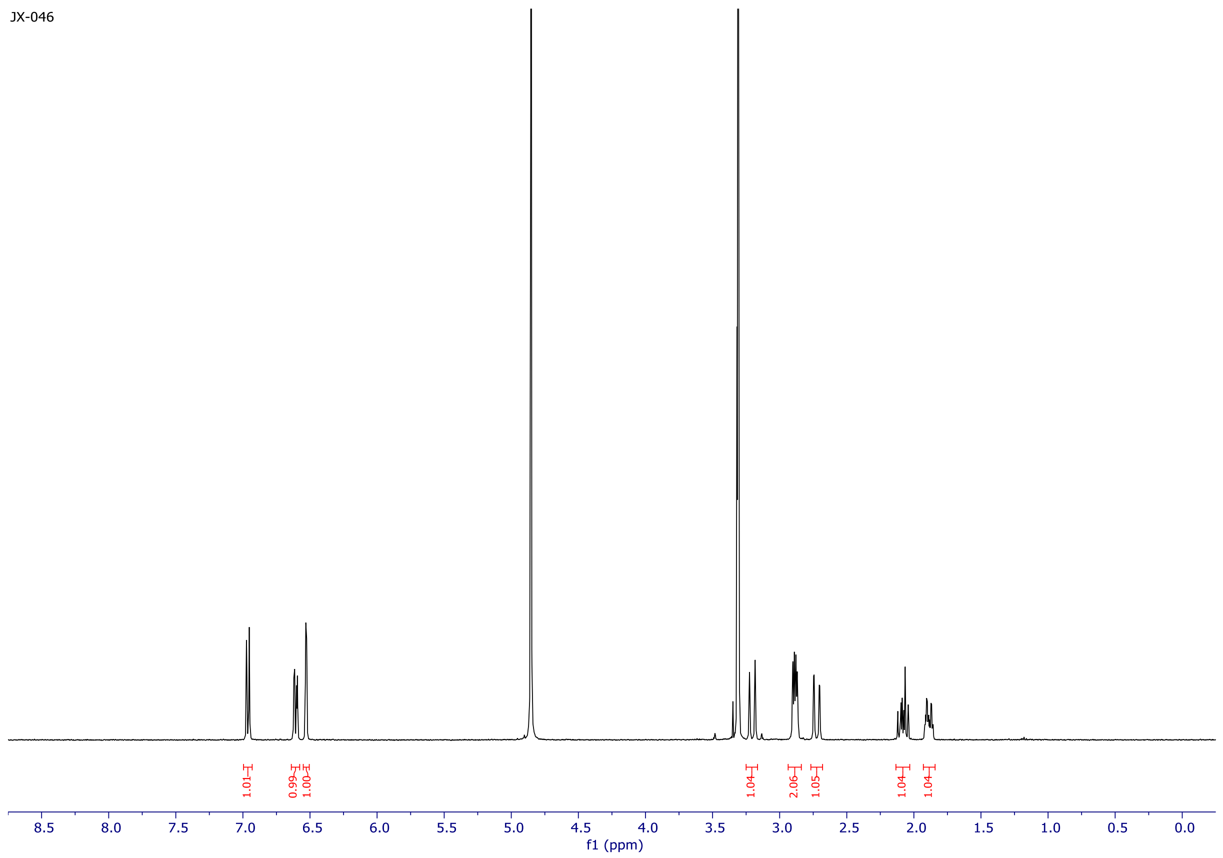
JX-046
1.01
0.99
1.00
1.04
2.06
1.05
1.04
1.04
8.5
8.0
7.5
7.0
6.5
6.0
5.5
5.0
4.5
4.0
3.5
3.0
2.5
2.0
1.5
1.0
0.5
0.0
f1 (ppm)

JX-046

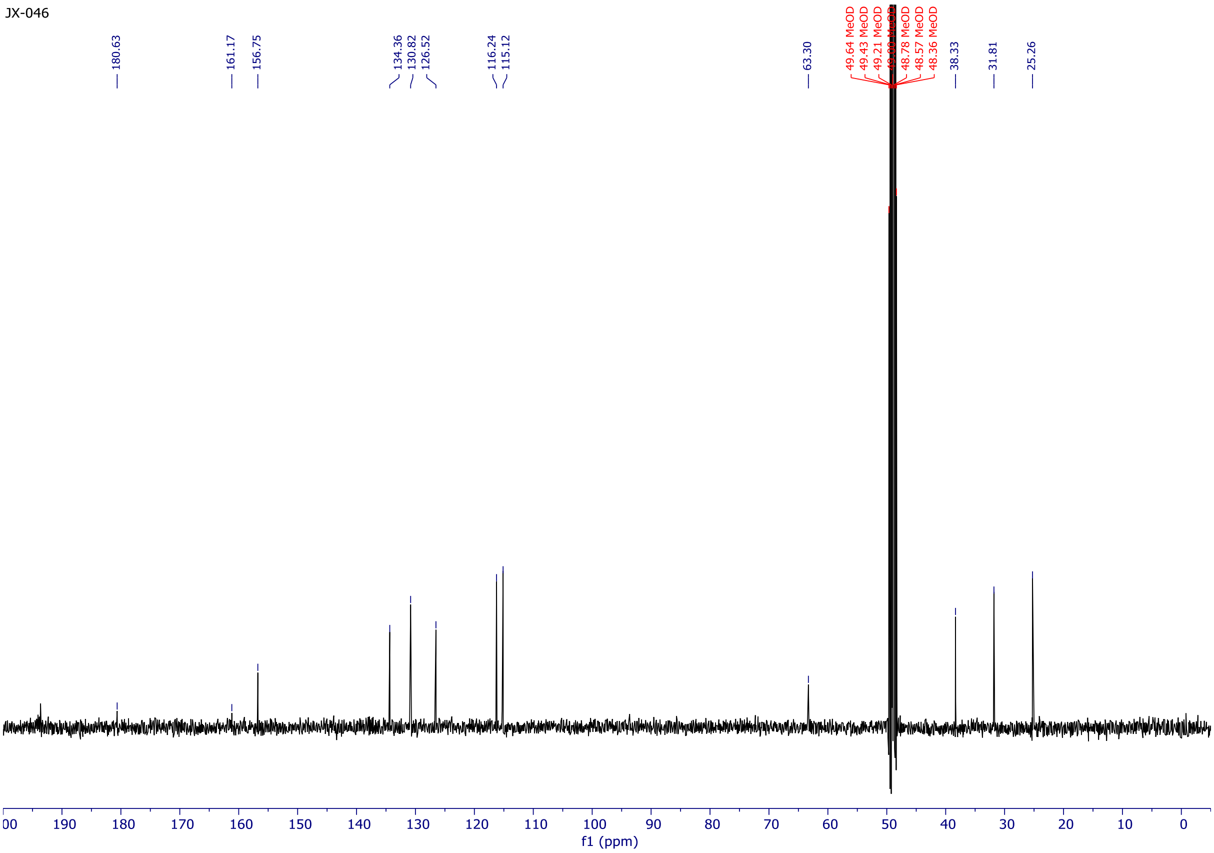

180.63
161.17
156.75
134.36
130.82
126.52
116.24
115.12
63.30
49.64 MeOD
49.43 MeOD
49.21 MeOD
48.78 MeOD
48.57 MeOD
48.36 MeOD
38.33
31.81
25.26
190
180
170
160
150
140
130
120
110
100
90
80
70
60
50
40
30
20
10
0
f1 (ppm)

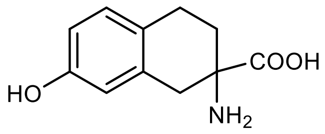

JX-048

2-Amino-7-hydroxy-1,2,3,4-tetrahydronaphthalene-2-carboxylic acid (JX-046): To a suspension of **JX-046** (225 mg, 0.969 mmol, 1.0 equiv) in H_2O (10.0 mL) in a microwave vial was added $Ba(OH)_2{\cdot}8H_2O$ (1.528 g, 4.844 mmol, 5.0 equiv) and the vial was sealed. After 1 h of microwave irradiation at 150 °C, the reaction mixture was concentrated under reduced pressure to obtain 1.33 g of crude product. 798 mg of this material was purified by flash column chromatography ($CHCl_3$/MeOH/H_2O 70:30:5 → 60:40:8) to yield **JX-048** (120 mg, 0.579 mmol, quant.) as a beige solid.

TLC (SiO_2; $CHCl_3$/MeOH/H_2O 70:30:5, UV, $KMnO_4$ stain): R_f = 0.16.

^{1}H-NMR (400 MHz, $(CD_3)_2SO$): δ (ppm) = 9.21 (brs, 1H), 7.62 (brs, 2H), 6.85 (d, *J* = 8.3 Hz, 1H), 6.53 (dd, *J* = 8.2, 2.5 Hz, 1H), 6.46 (d, *J* = 2.5 Hz, 1H), 3.22 (d, *J* = 17.3 Hz, 1H), 2.75 – 2.53 (m, 3H), 2.03 (ddd, *J* = 13.5, 9.7, 6.6 Hz, 1H), 1.81 (ddd, *J* = 13.3, 5.1, 5.1 Hz, 1H).

^{13}C-NMR (101 MHz, $(CD_3)_2SO$): δ (ppm) = 172.72, 155.40, 134.54, 129.25, 125.06, 114.99, 113.25, 58.18, 36.02, 29.74, 23.94.

IR (solid): υ (cm^{-1}) = 3182, 3049, 2947, 2826, 2746, 2564, 1639, 1603, 1586, 1514, 1500, 1458, 1441, 1396, 1335, 1286, 1270, 1246, 1227, 1169, 1152, 1110 ,1088, 950, 876, 853, 818, 775, 750, 705, 692, 620, 607, 545.

MS (ESI): Calcd for $C_{11}H_{14}NO_3$ $[M+H]^+$, 208.1 *m/z*; found 208.2 *m/z* (direct injection). No molecular ion was detectable in the HRMS, probably solubility issues.

JX-048

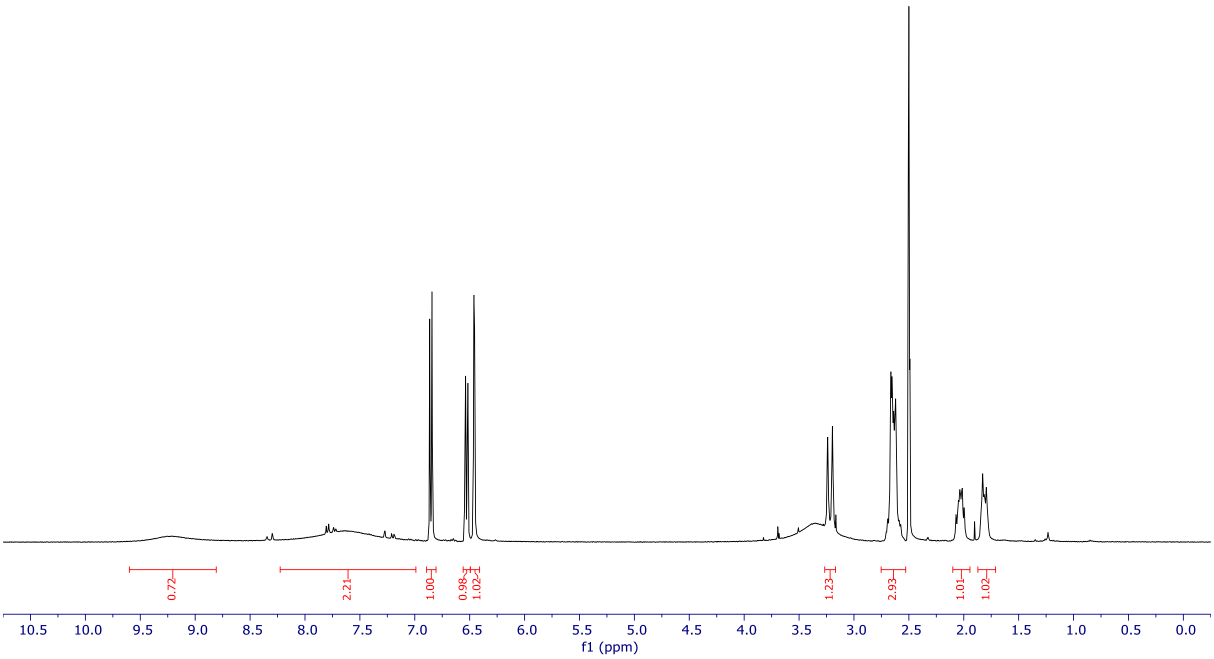

0.72
2.21
1.00
0.98
1.02
1.23
2.93
1.01
1.02
10.5
10.0
9.5
9.0
8.5
8.0
7.5
7.0
6.5
6.0
5.5
5.0
4.5
4.0
3.5
3.0
2.5
2.0
1.5
1.0
0.5
0.0
f1 (ppm)

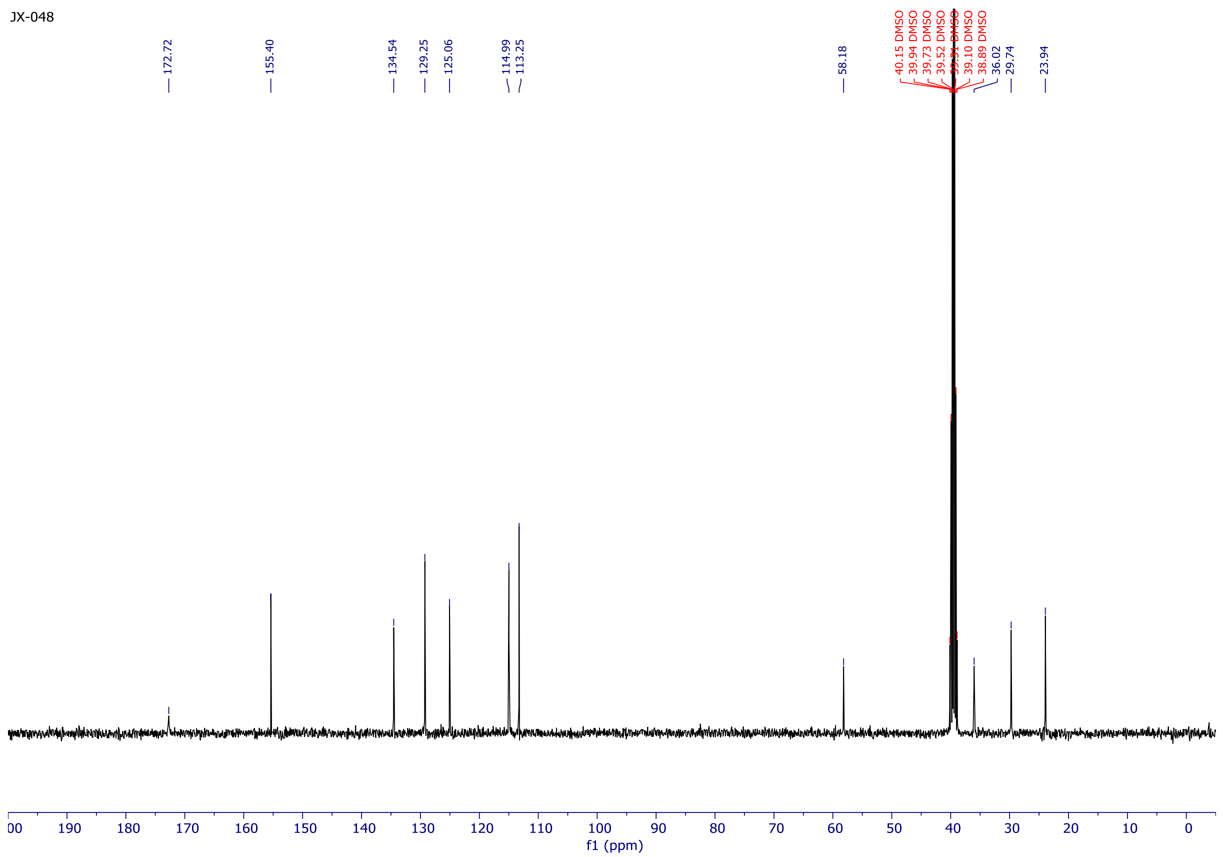
JX-048
172.72
155.40
134.54
129.25
125.06
114.99
113.25
58.18
40.15 DMSO
39.94 DMSO
39.73 DMSO
39.52 DMSO
39.31 DMSO
39.10 DMSO
38.89 DMSO
36.02
29.74
23.94
f1 (ppm)

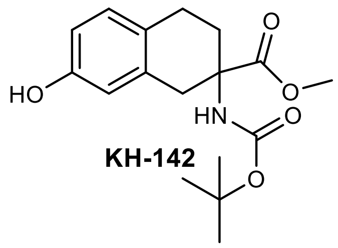

KH-142

Methyl 2-((tert-butoxycarbonyl)amino)-7-hydroxy-1,2,3,4-tetrahydronaphthalene-2-carboxylate (KH-142): To a solution of **JX-048** (500 mg, 2.41 mmol, 1.0 equiv) in dry MeOH (10.0 mL) was added $SOCl_2$ (1.0 mL, 14 mmol, 5.7 equiv) dropwise under an argon atmosphere over 3 min and the mixture was heated to reflux. After 3 h, the solvent was evaporated under reduced pressure and the residue was redissolved in dry MeOH (10.0 mL) and Et_3N (1.3 mL, 9.3 mmol, 3.9 equiv) was added followed by Boc_2O (944 mg, 4.32 mmol, 1.8 equiv) and the mixture was stirred at rt under argon atmosphere for 20 h. The solvent was then evaporated under reduced pressure and the residue was diluted with H_2O (20 mL), the pH was adjusted to 2 with 1 M HCl (2.0 mL), and the mixture was extracted with EtOAc (2 × 20 mL). The combined organic phases were washed with H_2O (2x), dried with $MgSO_4$, filtered, and concentrated under reduced pressure. The crude product was purified by flash column chromatography (CH_2Cl_2/acetone 9:1) to yield **KH-142** (505 mg, 1.57 mmol, 65%) as a beige solid.

TLC (SiO_2; CH_2Cl_2/MeOH 95:5, UV, $KMnO_4$ stain): R_f = 0.34.

^{1}H-NMR (400 MHz, $CDCl_3$): δ (ppm) = 6.96 (d, *J* = 8.3 Hz, 1H), 6.65 (dd, *J* = 8.3, 2.7 Hz, 1H), 6.54 (d, *J* = 2.7 Hz, 1H), 4.82 (brs, 1H), 4.43 (brs, 1H), 3.76 (s, 3H), 3.25 (d, *J* = 16.8 Hz, 1H), 2.90 (d, *J* = 16.9 Hz, 1H), 2.80 – 2.72 (m, 2H), 2.49 – 2.35 (m, 1H), 2.08 (ddd, *J* = 13.5, 9.1, 9.1 Hz, 1H), 1.42 (s, 9H).

^{13}C-NMR (101 MHz, $CDCl_3$): δ (ppm) = 174.76, 155.22, 154.11, 130.02, 126.86, 115.68, 114.11, 76.79, 57.94, 52.66, 38.21, 29.06, 28.40, 24.49. The signal for one quaternary C is missing from the spectrum.

IR (thin film): υ (cm^{-1}) = 3360, 2978, 1693, 1615, 1591, 1505, 1454, 1392, 1367, 1254, 1164, 1109, 1065, 914, 852, 817, 734.

HR-MS (ESI): Calcd for $C_{17}H_{23}NNaO_5$ $[M+Na]^+$, 344.1468 *m/z*; found, 344.1462 *m/z*.

KH-142-1

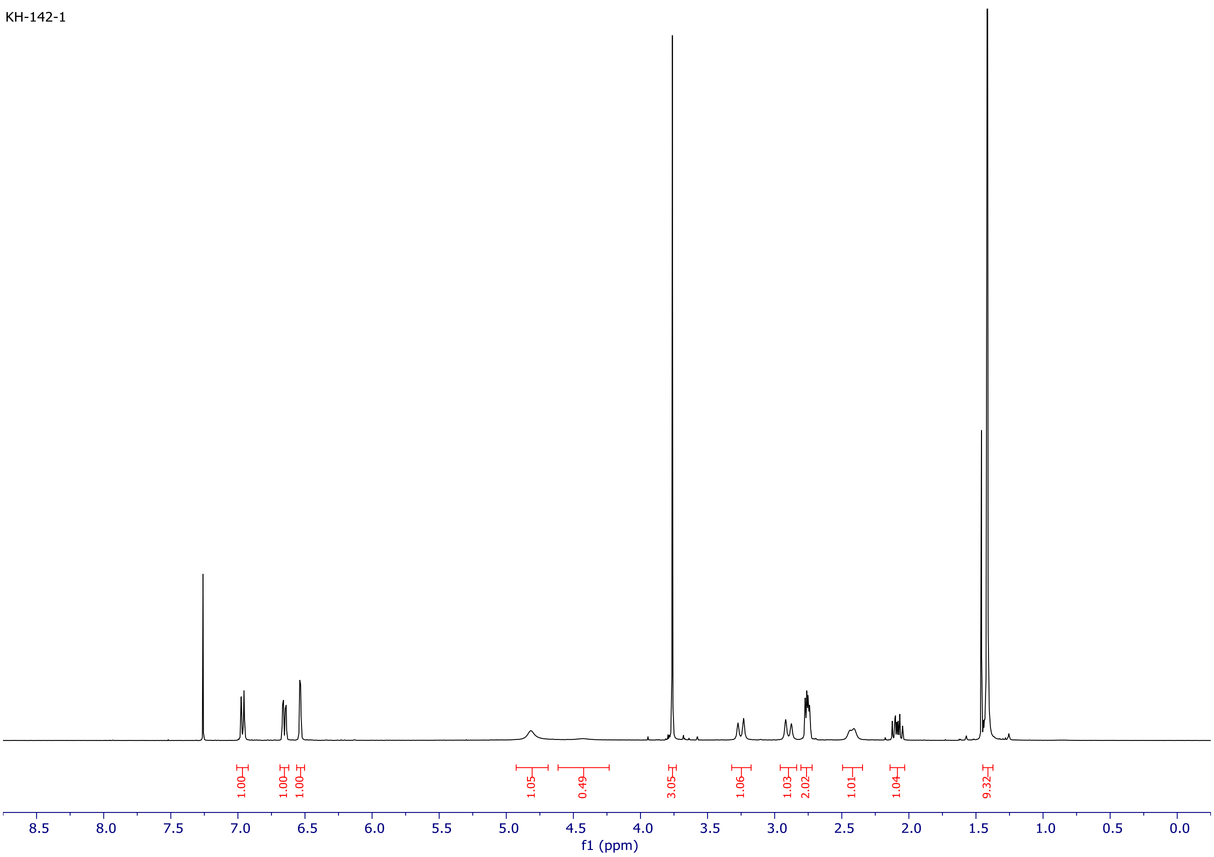

1.00
1.00
1.00
1.05
0.49
3.05
1.06
1.03
2.02
1.01
1.04
9.32
8.5
8.0
7.5
7.0
6.5
6.0
5.5
5.0
4.5
4.0
3.5
3.0
2.5
2.0
1.5
1.0
0.5
0.0
f1 (ppm)

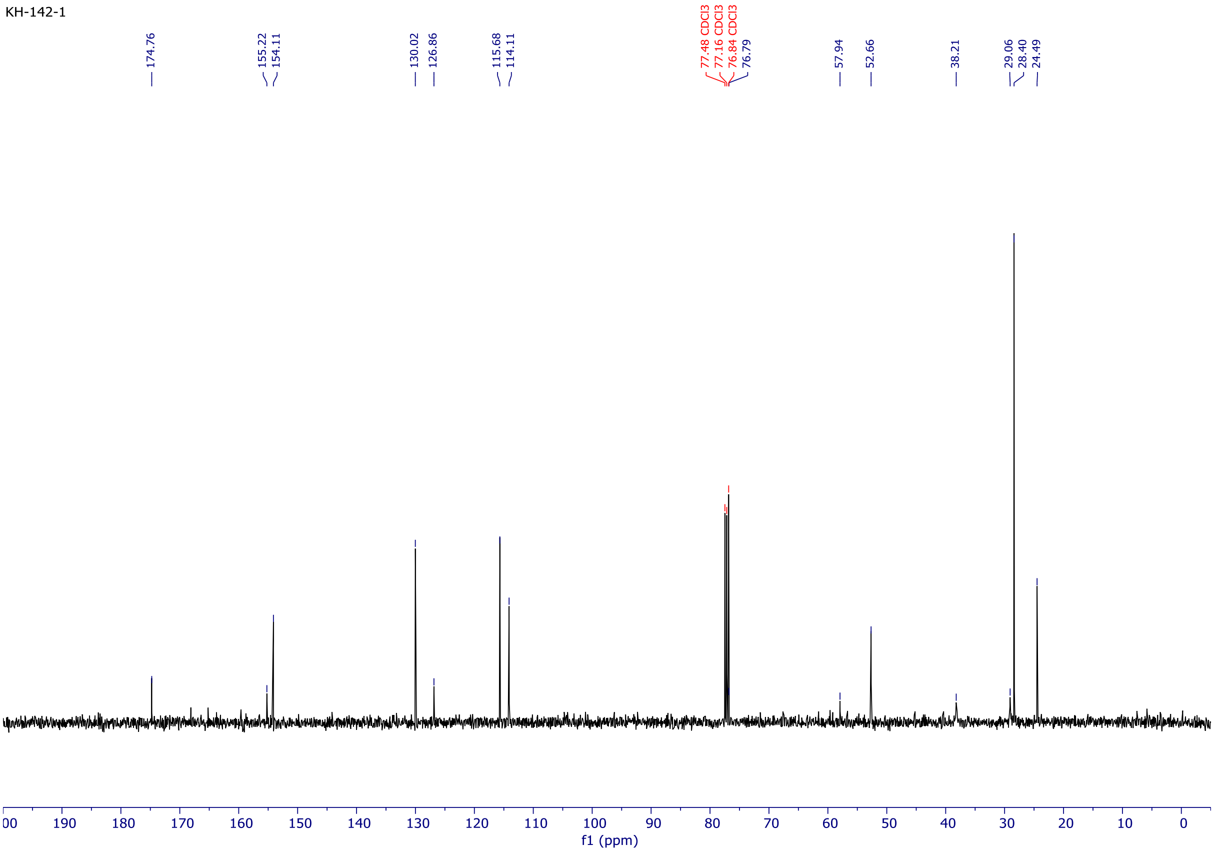

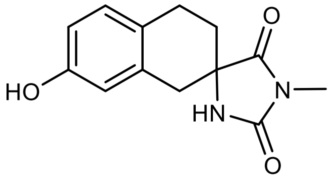

JX-059

7'-Hydroxy-1-methyl-3',4'-dihydro-1'H-spiro[imidazolidine-4,2'-naphthalene]-2,5-dione (JX-059): To a stirred solution of **JX-046** (642 mg, 2.76 mmol, 1.0 equiv) in dry DMF (18 mL) was added K_2CO_3 (764 mg, 5.52 mmol, 2.0 equiv) in one portion at rt under argon and the mixture was continued to stir. After 10 min, MeI (0.17 mL, 2.8 mmol, 1.0 equiv) was added dropwise and the reaction mixture was heated to 35 °C. After 15 h, the solution was concentrated under reduced pressure, the residue was redissolved in EtOAc (150 mL), and the solution was washed with brine (3 × 100 mL). The organic layer was dried with $MgSO_4$, filtered, and concentrated under reduced pressure. The crude product was purified by flash column

chromatography (hexane/EtOAc 4:6 and CH_2Cl_2/MeOH 96:4) to yield **JX-059** (519 mg, 76%) as beige solid.

TLC (SiO_2; CH_2Cl_2/MeOH 95:5, UV, $KMnO_4$ stain): R_f = 0.18.

mp: 215.1-215.6 °C.

^{1}H-NMR (400 MHz, $(CD_3)_2SO$): δ (ppm) = 9.11 (s, 1H), 8.54 (s, 1H), 6.91 (d, *J* = 8.3 Hz, 1H), 6.56 (dd, *J* = 8.2, 2.6 Hz, 1H), 6.47 (d, *J* = 2.5 Hz, 1H), 3.05 (d, *J* = 17.0 Hz, 1H), 2.85 (s, 3H), 2.82 – 2.71 (m, 2H), 2.65 (d, *J* = 16.9 Hz, 1H), 1.91 (ddd, *J* = 13.1, 10.5, 6.9 Hz, 1H), 1.81 – 1.71 (m, 1H).

^{13}C-NMR (101 MHz, $(CD_3)_2SO$): δ (ppm) = 176.67, 156.05, 155.38, 133.34, 129.45, 124.84, 114.98, 113.69, 59.83, 36.99, 30.34, 24.16, 23.85.

IR (thin film): υ (cm^{-1}) = 3287, 2928, 1768, 1615, 1590, 1504, 1466, 1396, 1348, 1313, 1273, 1224, 1155, 1021, 1001, 764, 718, 642, 589.

HR-MS (ESI): Calcd for $C_{13}H_{14}N_2NaO_3$ $[M+Na]^+$, 269.0897 *m/z*; found, 269.0897 *m/z*.

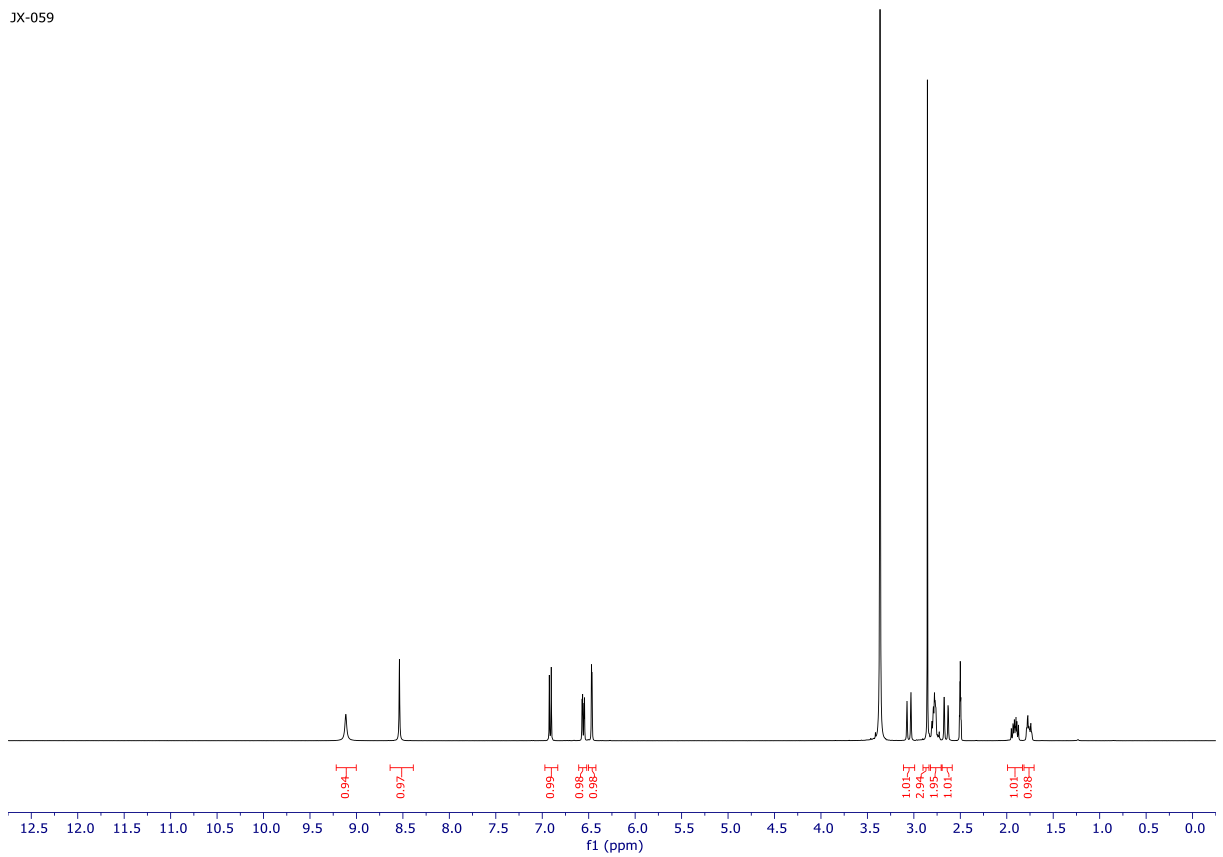
JX-059
0.94
0.97
0.99
0.98
0.98
1.01
2.94
1.95
1.01
1.01
0.98
12.5 12.0 11.5 11.0 10.5 10.0 9.5 9.0 8.5 8.0 7.5 7.0 6.5 6.0 5.5 5.0 4.5 4.0 3.5 3.0 2.5 2.0 1.5 1.0 0.5 0.0
f1 (ppm)

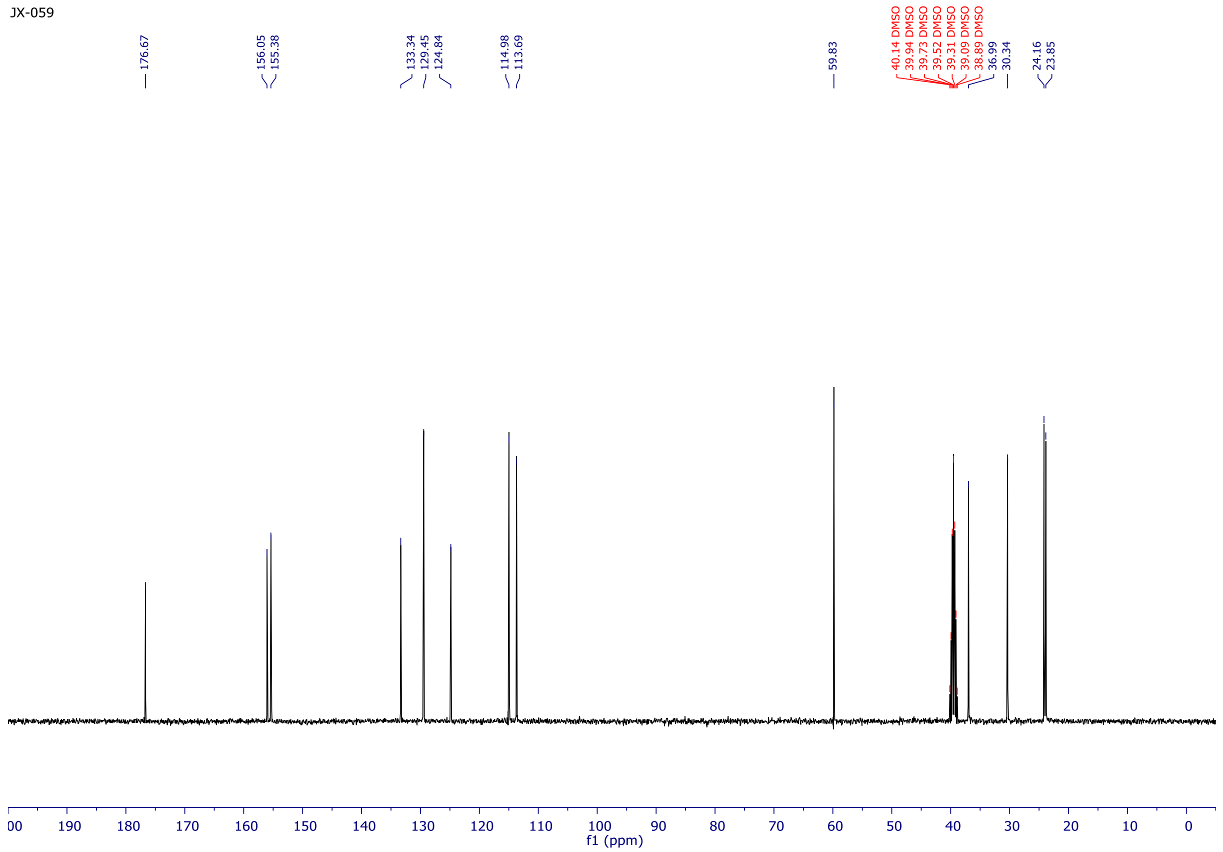
JX-059
176.67
156.05
155.38
133.34
129.45
124.84
114.98
113.69
59.83
40.14 DMSO
39.94 DMSO
39.73 DMSO
39.52 DMSO
39.31 DMSO
39.09 DMSO
38.89 DMSO
36.99
30.34
24.16
23.85
f1 (ppm)

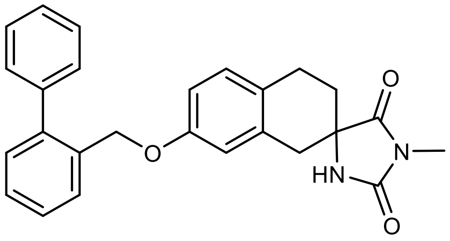

JX-065

7'-([1,1'-Biphenyl]-2-ylmethoxy)-1-methyl-3',4'-dihydro-1'H-spiro[imidazolidine-4,2'-naphthalene]-2,5-dione (JX-065): To a stirred solution of **JX-059** (52 mg, 0.21 mmol, 1.0 equiv) in dry DMF (2.0 mL) was added Cs_2CO_3 (103 mg, 0.317 mmol, 1.5 equiv) in one portion at rt under argon and the mixture was continued to stir. After 10 min, 2-phenylbenzyl bromide (0.040 mL, 0.22 mmol, 1.05 equiv) was added dropwise and the reaction mixture was heated to 60 °C. After 15 h, the reaction was allowed to cool to rt, the mixture was diluted with EtOAc (100 mL), and the solution was washed with brine (3 × 25 mL). The organic layer was dried with $MgSO_4$, filtered, and concentrated under reduced pressure. The crude product was purified by flash column chromatography (CH_2Cl_2/MeOH 98:2) to yield **JX-065** (76 mg, 87%) as a white beige solid.

TLC (SiO_2; CH_2Cl_2/MeOH 98:2, UV, $KMnO_4$ stain): R_f = 0.43.

mp: 112.8-116.8 °C.

^{1}H-NMR (400 MHz, $CDCl_3$): δ (ppm) = 7.60 (dd, J = 5.5, 3.6 Hz, 1H), 7.48 – 7.30 (m, 8H), 7.01 (d, J = 8.5 Hz, 1H), 6.71 (dd, J = 8.5, 2.7 Hz, 1H), 6.52 (d, J = 2.6 Hz, 1H), 5.59 (s, 1H), 4.90 (s, 2H), 3.30 (d, J = 16.4 Hz, 1H), 3.04 (s, 3H), 2.96 (ddd, J = 17.4, 6.7, 2.7 Hz, 1H), 2.81 (ddd, J = 16.4, 11.4, 5.6 Hz, 1H), 2.63 (dd, J = 16.6, 2.3 Hz, 1H), 2.19 (ddd, J = 13.4, 12.0, 6.7 Hz, 1H), 1.87 (dddd, J = 13.4, 6.2, 2.6, 2.6 Hz, 1H).

^{13}C-NMR (101 MHz, $CDCl_3$): δ (ppm) = 176.56, 157.31, 156.64, 141.98, 140.56, 134.06, 132.82, 130.26, 130.04, 129.39, 129.28, 128.42, 128.29, 127.83, 127.48, 125.99, 115.16, 114.41, 68.25, 60.86, 37.80, 30.29, 24.84, 24.24.

IR (thin film): υ (cm^{-1}) = 3291, 2928, 1772, 1704, 1610, 1502, 1456, 1394, 1347, 1308, 1265, 1225, 1160, 1115, 1018, 999, 909, 750, 730, 704, 647, 585.

HR-MS (ESI): Calcd for $C_{26}H_{25}N_2O_3$ $[M+H]^+$, 413.1860 *m/z*; found, 413.1862 *m/z*.

JX-065

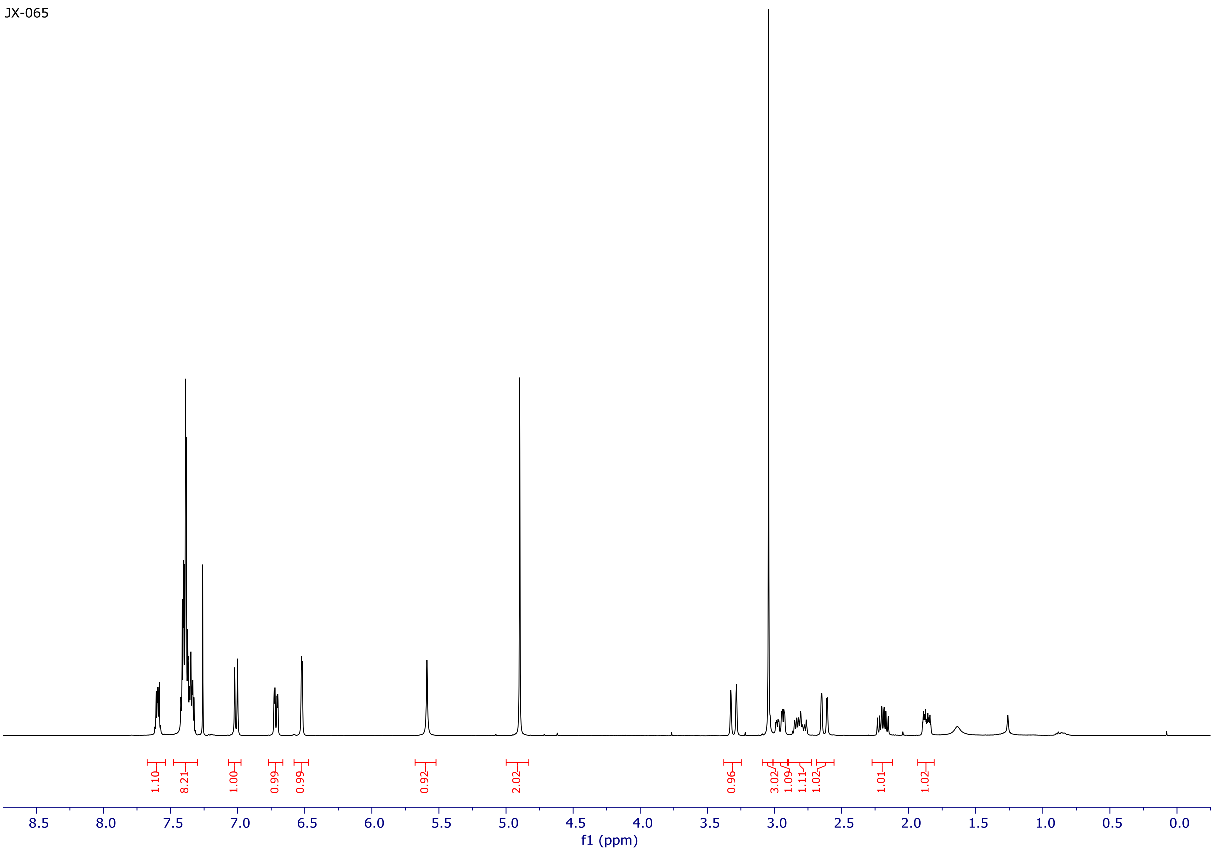
1.10
8.21
1.00
0.99
0.99
0.92
2.02
0.96
3.02
1.09
1.11
1.02
1.01
1.02
8.5
8.0
7.5
7.0
6.5
6.0
5.5
5.0
4.5
4.0
3.5
3.0
2.5
2.0
1.5
1.0
0.5
0.0
f1 (ppm)

JX-065

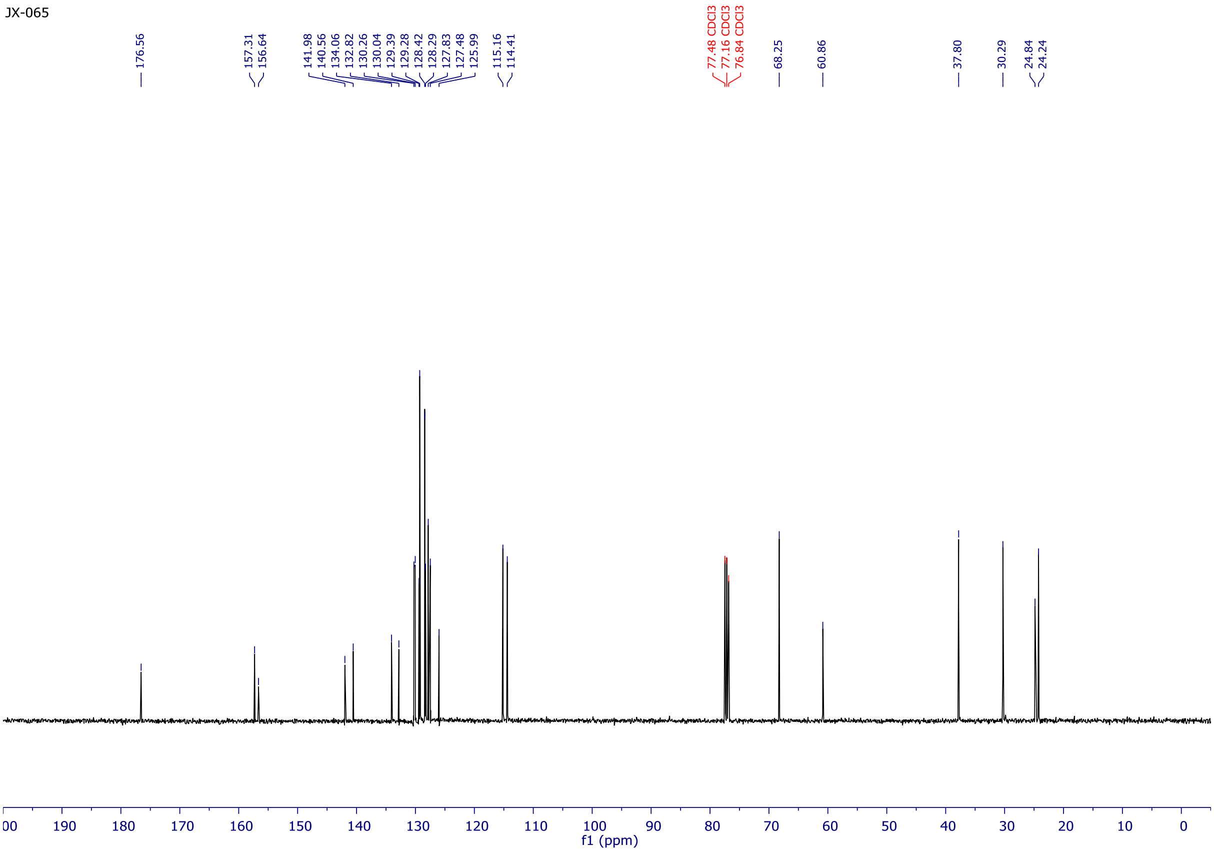
176.56
157.31
156.64
141.98
140.56
134.06
132.82
130.26
130.04
129.39
129.28
128.42
128.29
127.83
127.48
125.99
115.16
114.41
77.48 CDCl3
77.16 CDCl3
76.84 CDCl3
68.25
60.86
37.80
30.29
24.84
24.24
f1 (ppm)

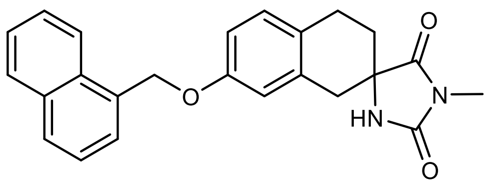

JX-070

1-Methyl-7'-(naphthalen-1-ylmethoxy)-3',4'-dihydro-1'H-spiro[imidazolidine-4,2'-naphthalene]-2,5-dione (JX-070): To a solution of **JX-059** (50 mg, 0.20 mmol, 1.0 equiv) in dry DMF (2.0 mL) was added Cs_2CO_3 (100 mg, 0.306 mmol, 1.5 equiv) in one portion at rt under argon and the mixture was continued to stir. After 10 min, 2-methylnaphthalene bromide (0.047 mg, 0.21 mmol, 1.05 equiv) was added and the reaction mixture was heated to 60 °C. After 15 h, the reaction was allowed to cool to rt, the mixture was diluted with EtOAc (100 mL), and the solution was washed with brine (3 × 25 mL). The organic layer was dried with $MgSO_4$, filtered, and concentrated under reduced pressure. The crude product was purified by flash column chromatography (CH_2Cl_2/MeOH 98:2) to yield **JX-070** (69 mg, 87%) as a white solid.

TLC (SiO_2; CH_2Cl_2/MeOH 98:2, UV, $KMnO_4$ stain): R_f = 0.42.

mp: 208.2-208.9 °C.

^{1}H-NMR (400 MHz, $CDCl_3$): δ (ppm) = 8.07 – 7.98 (m, 1H), 7.92 – 7.87 (m, 1H), 7.86 (d, *J* = 8.2 Hz, 1H), 7.58 (dd, *J* = 7.0, 1.2 Hz, 1H), 7.56 – 7.49 (m, 2H), 7.46 (dd, *J* = 8.2, 7.0 Hz, 1H), 7.08 (d, *J* = 8.4 Hz, 1H), 6.90 (dd, *J* = 8.4, 2.7 Hz, 1H), 6.75 (d, *J* = 2.6 Hz, 1H), 5.74 (s, 1H), 5.45 (s, 2H), 3.36 (d, *J* = 16.5 Hz, 1H), 3.05 (s, 3H), 2.99 (ddd, *J* = 17.3, 6.6, 2.8 Hz, 1H), 2.85 (ddd, *J* = 17.5, 12.0, 6.2 Hz, 1H), 2.70 (dd, *J* = 16.6, 2.4 Hz, 1H), 2.22 (ddd, *J* = 13.4, 12.0, 6.7 Hz, 1H), 1.89 (dddd, *J* = 13.3, 6.2, 2.6, 2.6 Hz, 1H).

^{13}C-NMR (101 MHz, $CDCl_3$): δ (ppm) = 176.57, 157.55, 156.69, 133.91, 133.02, 132.28, 131.61, 130.16, 129.20, 128.85, 126.71, 126.60, 126.28, 126.06, 125.43, 123.75, 115.31, 114.34, 68.85, 60.86, 37.86, 30.32, 24.83, 24.27.

IR (thin film): υ (cm^{-1}) = 3293, 2927, 1771, 1704, 1609, 1502, 1461, 1394, 1344, 1308, 1265, 1224, 1160, 1113, 1017, 999, 794, 755, 715, 636, 586.

HR-MS (ESI): Calcd for $C_{24}H_{23}N_2O_3$ $[M+H]^+$, 387.1703 *m/z*; found, 387.1697 *m/z*.

JX-070

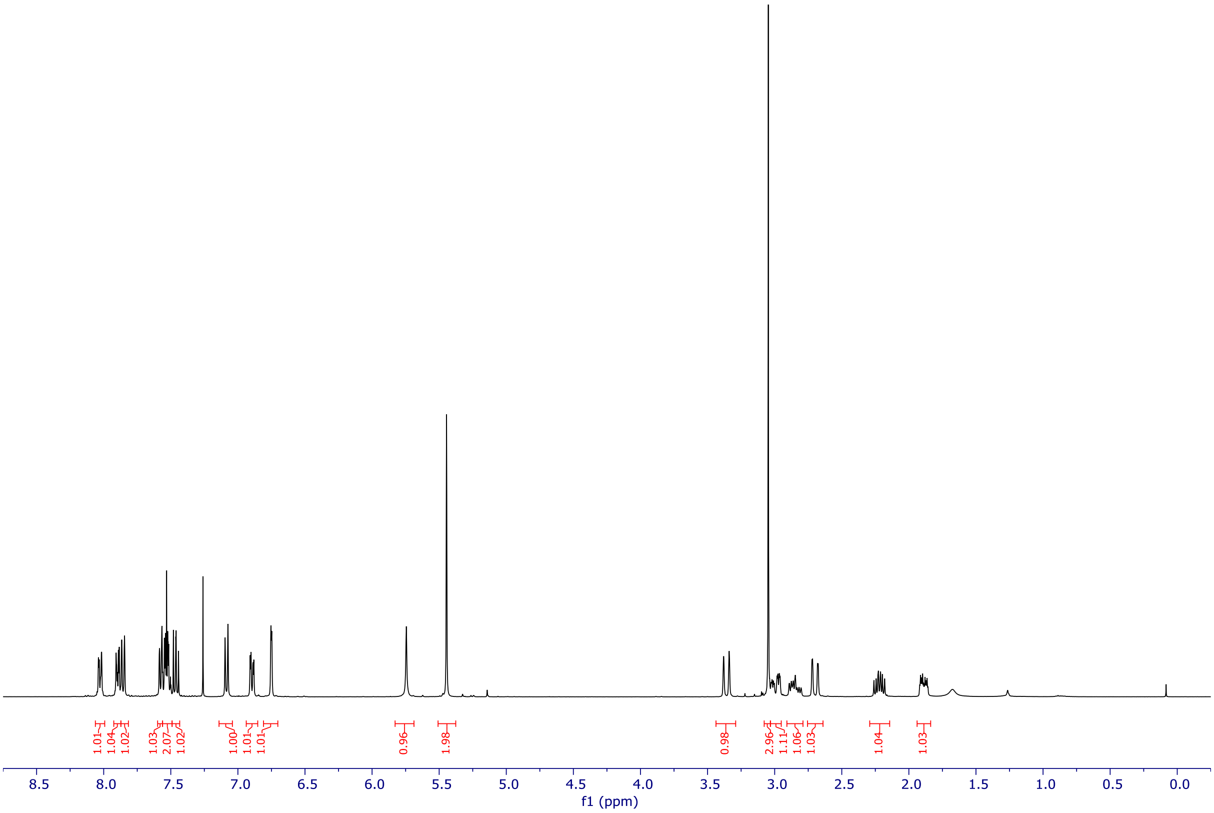

1.01
1.04
1.02
1.03
2.07
1.02
1.00
1.01
1.01
0.96
1.98
0.98
2.96
1.11
1.06
1.03
1.04
1.03
8.5
8.0
7.5
7.0
6.5
6.0
5.5
5.0
4.5
4.0
3.5
3.0
2.5
2.0
1.5
1.0
0.5
0.0
f1 (ppm)

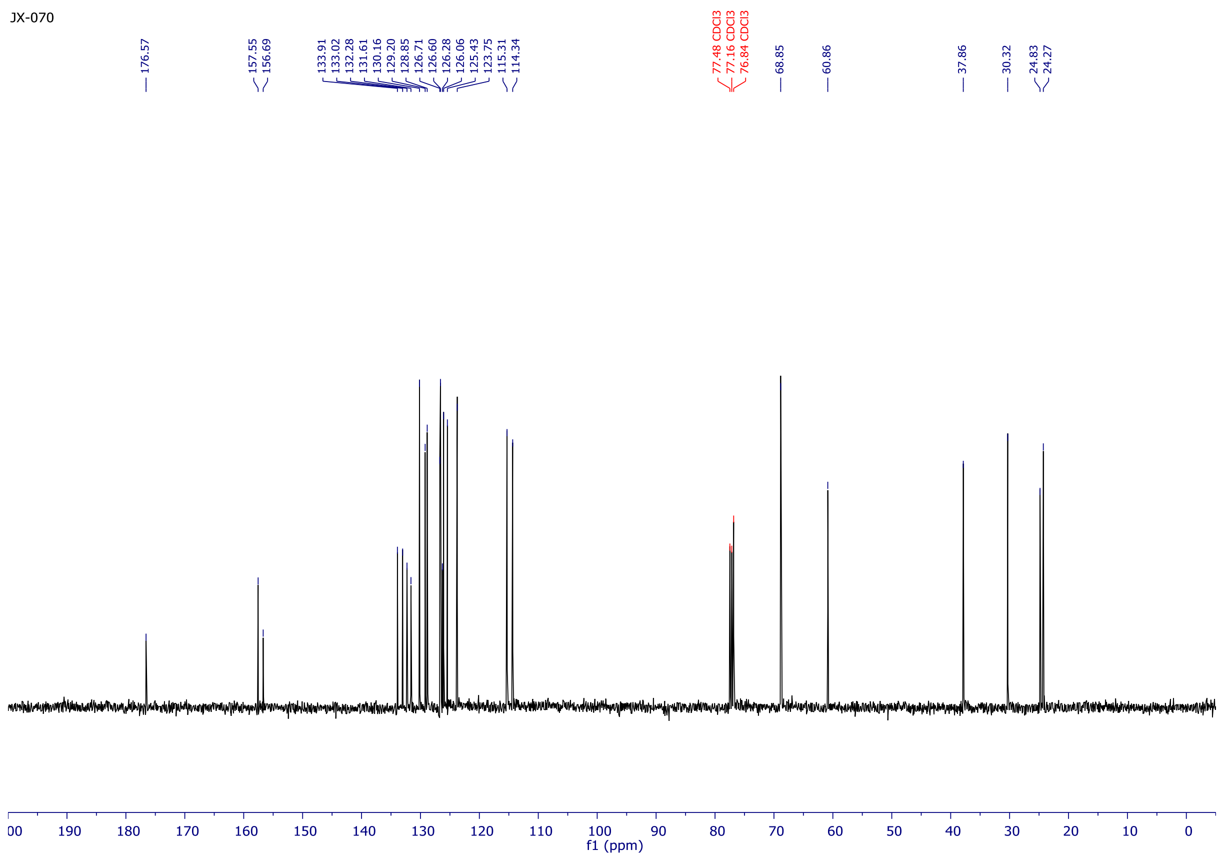
JX-070
176.57
157.55
156.69
133.91
133.02
132.28
131.61
130.16
129.20
128.85
126.71
126.60
126.28
126.06
125.43
123.75
115.31
114.34
77.48 CDCl3
77.16 CDCl3
76.84 CDCl3
68.85
60.86
37.86
30.32
24.83
24.27
f1 (ppm)

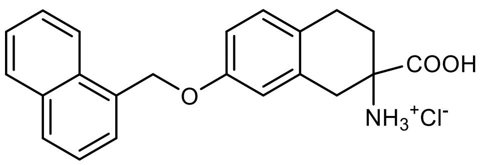

JX-075

2-Carboxy-7-(naphthalen-1-ylmethoxy)-1,2,3,4-tetrahydronaphthalen-2-aminium chloride (JX-075): A sealed microwave vial containing **JX-070** (21 mg, 0.053 mmol, 1.0 equiv) and $Ba(OH)_2{\cdot}8H_2O$ (84 mg, 0.27 mmol, 5.0 equiv) in H_2O/EtOH (2:1, 0.5 mL) was irradiated with microwaves for 2 h of at 150 °C. The pH was then adjusted to 1 using HCl (aq., 3 M) and the resulting mixture was extracted with EtOAc (3 × 25 mL). The combined organic phases were washed with brine (3 × 10 mL), dried with $MgSO_4$, filtered, and concentrated under reduced pressure. The crude product was purified by flash column chromatography ($CHCl_3$/MeOH/H_2O 70:30:5) to yield **JX-075** (16 mg, 0.045 mmol, 85%) as a white solid.

To ascertain that all of the material is present as the HCl-salt, **JX-075** was suspended in HCl in CPME (3 M, 1.5 mL) and stirred at rt under argon atmosphere. After the appearance of a precipitate, the mixture was filtered over celite, and the pad was washed with CPME. The remaining solid was washed down with MeOH and the filtrate was concentrated under reduced pressure to yield **JX-075** (17 mg, 0.044 mmol, 82%) as a white solid.

TLC (SiO_2; $CHCl_3$/MeOH/H_2O 70:30:5), UV, $KMnO_4$ stain): R_f = 0.39.

^{1}H-NMR (400 MHz, CD_3OD): δ (ppm) = 8.10 – 8.03 (m, 1H), 7.93 – 7.87 (m, 1H), 7.85 (d, *J* = 8.2 Hz, 1H), 7.59 (dd, *J* = 7.0, 1.1 Hz, 1H), 7.56 – 7.48 (m, 2H), 7.44 (dd, *J* = 8.3, 7.0 Hz, 1H), 7.12 (d, *J* = 8.5 Hz, 1H), 6.94 (dd, *J* = 8.5, 2.6 Hz, 1H), 6.87 (d, *J* = 2.6 Hz, 1H), 5.49 (s, 2H), 3.48 (d, *J* = 17.1 Hz, 1H), 3.01 (d, *J* = 16.7 Hz, 1H), 2.97 (ddd, *J* = 17.3, 6.4, 6.4 Hz, 1H), 2.84 (ddd, *J* = 16.9, 10.1, 6.0 Hz, 1H), 2.38 (ddd, *J* = 13.9, 10.0, 6.5 Hz, 1H), 2.16 (dddd, *J* = 13.9, 5.6, 5.2, 1.7 Hz, 1H).

^{13}C-NMR (101 MHz, CD_3OD): δ (ppm) = 173.62, 158.94, 135.27, 133.95, 132.95, 131.13, 129.94, 129.65, 127.68, 127.35, 127.33, 126.92, 126.26, 124.85, 116.00, 115.58, 69.69, 59.53, 36.43, 30.40, 24.59.

IR (solid): υ (cm^{-1}) = 3043, 2914, 2854, 1731, 1671, 1611, 1578, 1456, 1445, 1389, 1338, 1274, 1241, 1222, 1158, 1117, 1068, 1017, 1005, 919, 848, 771, 736, 699, 544, 532, 451, 444.

HR-MS (ESI): Calcd for $C_{22}H_{21}NNaO_3$ $[M+Na]^+$, 370.1414 *m/z*; found, 370.1421 *m/z*.

JX-075

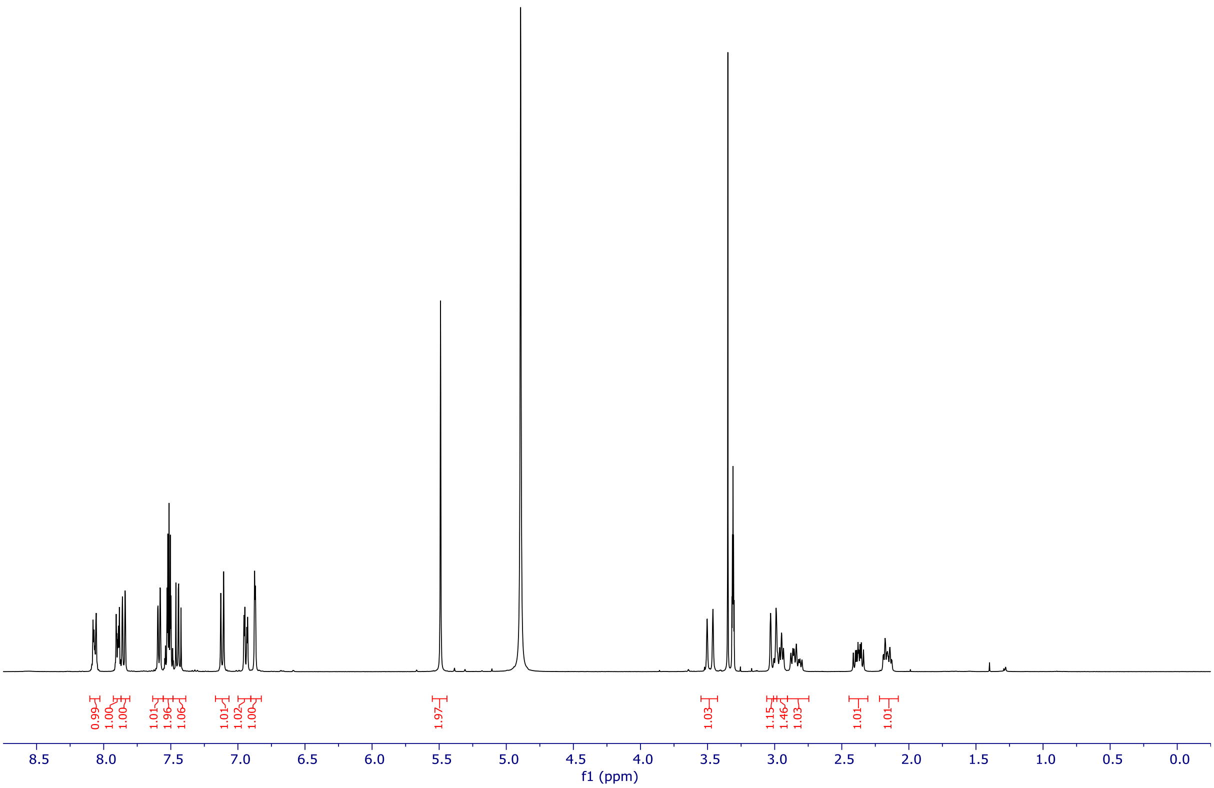

0.99
1.00
1.00
1.01
1.96
1.06
1.01
1.02
1.00
1.97
1.03
1.15
1.46
1.03
1.01
1.01
8.5
8.0
7.5
7.0
6.5
6.0
5.5
5.0
4.5
4.0
3.5
3.0
2.5
2.0
1.5
1.0
0.5
0.0
f1 (ppm)

JX-075

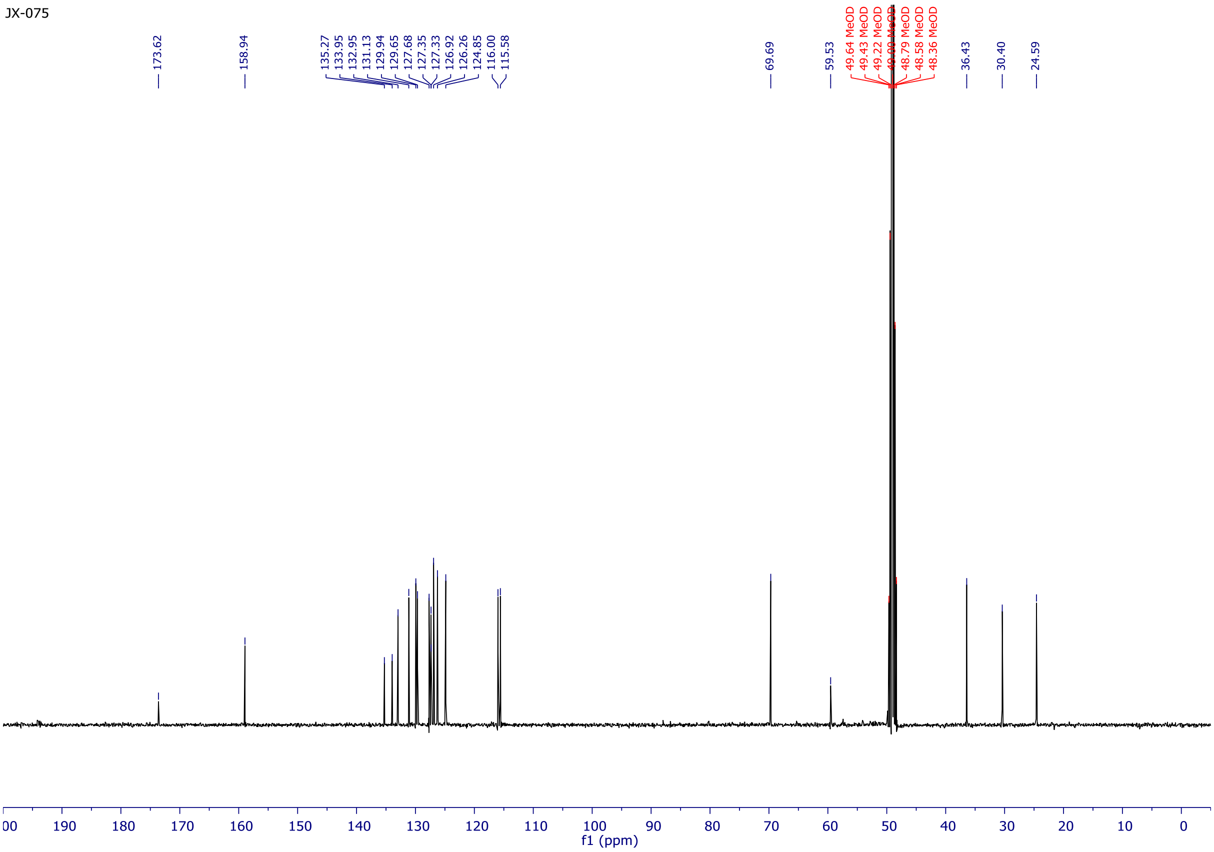
173.62
158.94
135.27
133.95
132.95
131.13
129.94
129.65
127.68
127.35
127.33
126.92
126.26
124.85
116.00
115.58
69.69
59.53
49.64 MeOD
49.43 MeOD
49.22 MeOD
49.00 MeOD
48.79 MeOD
48.58 MeOD
48.36 MeOD
36.43
30.40
24.59
00
190
180
170
160
150
140
130
120
110
100
90
80
70
60
50
40
30
20
10
0
f1 (ppm)

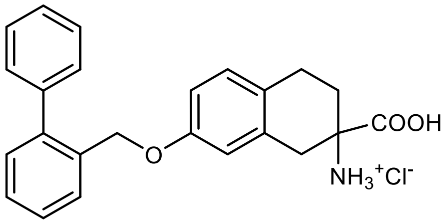

JX-078

7-([1,1'-Biphenyl]-2-ylmethoxy)-2-carboxy-1,2,3,4-tetrahydronaphthalen-2-aminium chloride (JX-078): In a microwave vial, **JX-065** (20 mg, 0.049 mmol, 1.0 equiv) and $Ba(OH)_2{\cdot}8H_2O$ (77 mg, 0.24 mmol, 5.0 equiv) was put in H_2O/EtOH (2:1, 0.5 mL) and the vial was sealed. After 2 h of irradiation at 150 °C in the microwave, the pH was adjusted to 1 using HCl (aq., 3 M). The resulting mixture was extracted with EtOAc (3 × 25 mL) and the combined organic phases were washed with brine (3 × 10 mL), dried with $MgSO_4$, filtered, and concentrated under reduced pressure. The crude product was purified by flash column chromatography ($CHCl_3$/MeOH/H_2O 70:30:5). to yield **JX-078** (13 mg, 0.034 mmol, 69%) as a white solid.

To ascertain that all of the material is present as the HCl-salt, **JX-078** was dissolved in HCl in CPME (3 M, 1 mL) and stirred at rt under argon atmosphere. After the appearance of a precipitate, the mixture was filtered over celite, and the pad was washed with CPME. The remaining solid was washed down with MeOH and concentrated under reduced pressure to yield **JX-078** (13 mg, 0.031 mmol, 63%) as a white solid.

TLC (SiO_2; $CHCl_3$/MeOH/H_2O 70:30:5), UV, $KMnO_4$ stain): R_f = 0.36.

^{1}H-NMR (400 MHz, CD_3OD): δ (ppm) = 7.61 – 7.50 (m, 1H), 7.43 – 7.32 (m, 7H), 7.32 – 7.27 (m, 1H), 7.05 (d, *J* = 8.4 Hz, 1H), 6.72 (dd, *J* = 8.4, 2.6 Hz, 1H), 6.61 (d, *J* = 2.6 Hz, 1H), 4.90 (s, 2H), 3.42 (d, *J* = 17.2 Hz, 1H), 3.02 – 2.87 (m, 1H), 2.93 (d, *J* = 17.6 Hz, 1H), 2.80 (ddd, *J* = 17.0, 10.2, 6.0 Hz, 1H), 2.36 (ddd, *J* = 14.0, 10.2, 6.5 Hz, 1H), 2.20 – 2.07 (m, 1H).

^{13}C-NMR (101 MHz, CD_3OD): δ (ppm) = 173.68, 158.68, 143.59, 142.00, 135.46, 132.83, 131.06, 130.68, 130.26, 129.28, 128.62, 128.40, 127.16, 115.61, 115.48, 69.23, 59.52, 36.39, 30.35, 24.52.

IR (solid): υ (cm^{-1}) = 3397, 3022, 2926, 1725, 1609, 1503, 1481, 1453, 1384, 1263, 1240, 1158, 1118, 1009, 950, 916, 875, 848, 811, 775, 748, 702, 616, 557, 542, 440, 414.

HR-MS (ESI): Calcd for $C_{24}H_{24}NO_3$ $[M+H]^+$, 374.1751 *m/z*; found, 374.1758 *m/z*.

JX-078

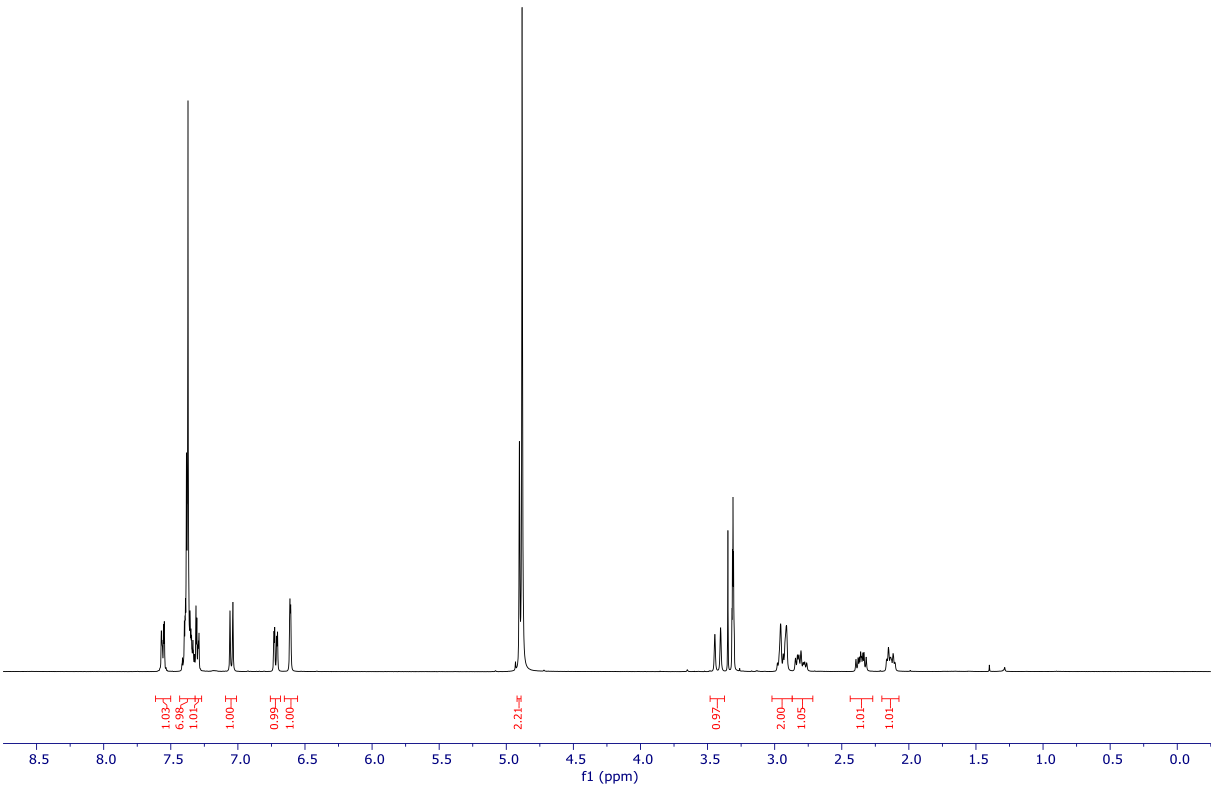
1.03
6.98
1.01
1.00
0.99
1.00
2.21
0.97
2.00
1.05
1.01
1.01
8.5
8.0
7.5
7.0
6.5
6.0
5.5
5.0
4.5
4.0
3.5
3.0
2.5
2.0
1.5
1.0
0.5
0.0
f1 (ppm)

JX-078

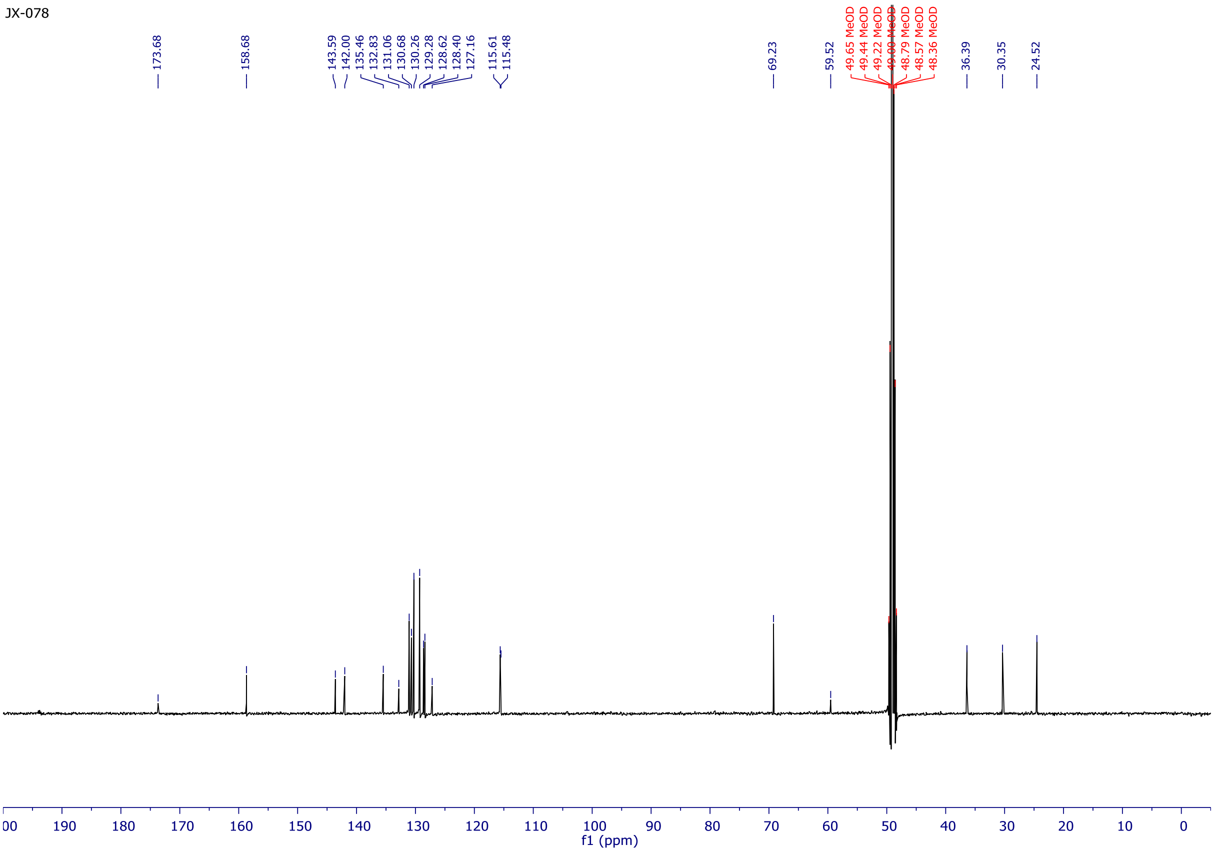
173.68
158.68
143.59
142.00
135.46
132.83
131.06
130.68
130.26
129.28
128.62
128.40
127.16
115.61
115.48
69.23
59.52
49.65 MeOD
49.44 MeOD
49.22 MeOD
49.00 MeOD
48.79 MeOD
48.57 MeOD
48.36 MeOD
36.39
30.35
24.52
f1 (ppm)

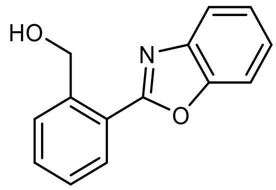

JX-107

(2-(Benzo[d]oxazol-2-yl)phenyl)methanol (JX-107): In a microwave vial, **2-chlorobenzoxazole** (0.10 mL, 0.88 mmol, 1.0 equiv), **2-(hydroxy-methyl)benzeneboronic acid** (200 mg, 1.32 mmol, 1.5 equiv), K_2CO_3 (243 mg, 1.76 mmol, 2.0 equiv), and $Pd(PPh_3)_4$ (51 mg, 0.044 mmol, 0.05 equiv) were dissolved in degassed 1,2-dimethoxyethane/H_2O (8:3, 13.0 mL) and the vial was sealed. After 30 min of microwave irradiation at 90 °C, the organic phase was separated from the aqueous phase, which was extracted with EtOAc (3 × 50 mL). The combined organic phases were washed with brine (3 × 25 mL), dried with $MgSO_4$, filtered, and concentrated under reduced pressure. The crude product was purified by flash column chromatography (CH_2Cl_2/MeOH 98:2) to yield **JX-107** (145 mg, 0.646 mmol, 73%) as a yellowish white solid.

TLC (SiO_2; hexane/EtOAc 7:3, UV, $KMnO_4$ stain): R_f = 0.39.

mp: 90.6-92.3 °C.

^{1}H-NMR (400 MHz, $CDCl_3$): δ (ppm) = 8.24 (ddd, *J* = 6.9, 1.4, 1.4 Hz, 1H), 7.81 – 7.75 (m, 1H), 7.65 – 7.59 (m, 1H), 7.55 – 7.45 (m, 3H), 7.43 – 7.36 (m, 2H), 6.13 (brs, 1H), 4.87 (s, 2H).

^{13}C-NMR (101 MHz, $CDCl_3$): δ (ppm) = 162.51, 150.28, 141.72, 141.38, 131.92, 131.12, 129.88, 128.38, 125.92, 125.83, 124.99, 120.14, 110.87, 64.81.

IR (thin film): υ (cm^{-1}) = 3386, 3064, 2954, 2870, 1614, 1548, 1486, 1475, 1454, 1442, 1343, 1301, 1270, 1244, 1203, 1192, 1126, 1108, 1055, 1024, 959, 924, 894, 836, 820, 798, 778, 759, 742, 725, 708, 607, 452, 427.

HR-MS (ESI): Calcd for $C_{14}H_{11}NNaO_2$ $[M+Na]^+$, 248.0682 *m/z*; found, 248.0685 *m/z*.

JX-107

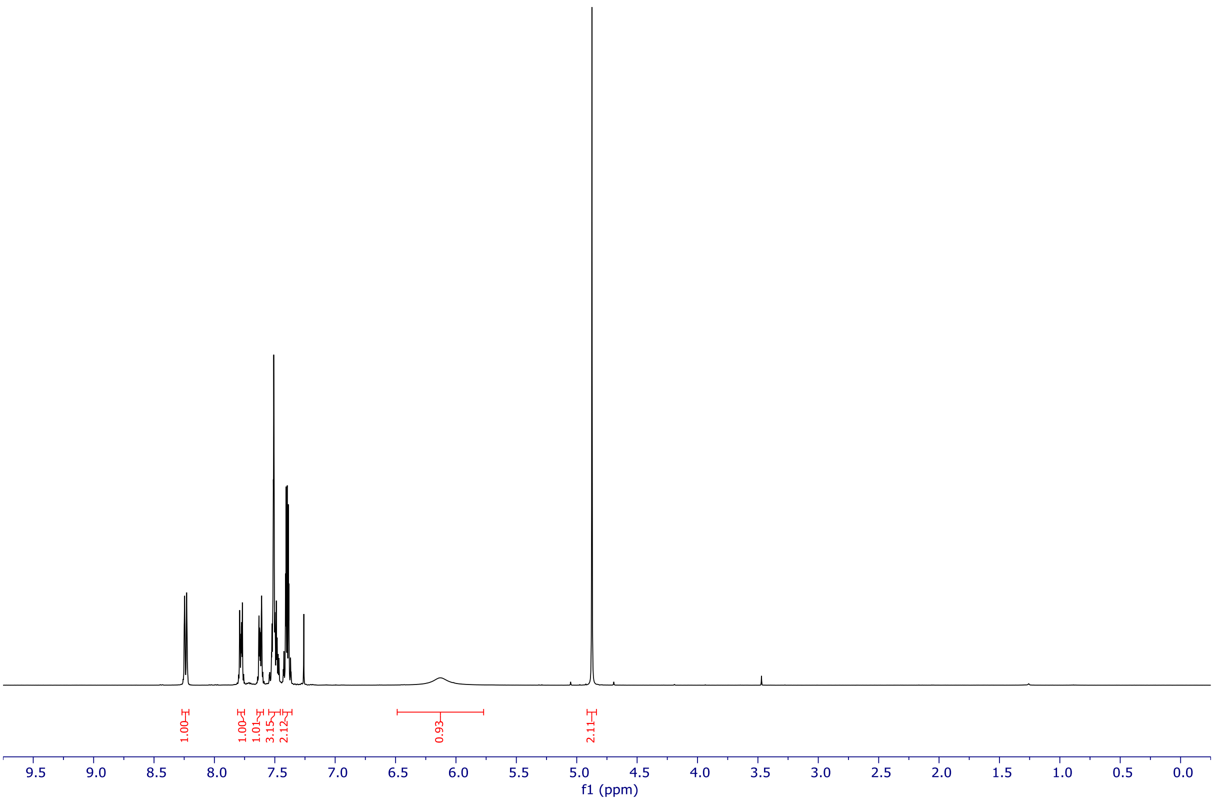
1.00
1.00
1.01
3.15
2.12
0.93
2.11
9.5
9.0
8.5
8.0
7.5
7.0
6.5
6.0
5.5
5.0
4.5
4.0
3.5
3.0
2.5
2.0
1.5
1.0
0.5
0.0
f1 (ppm)

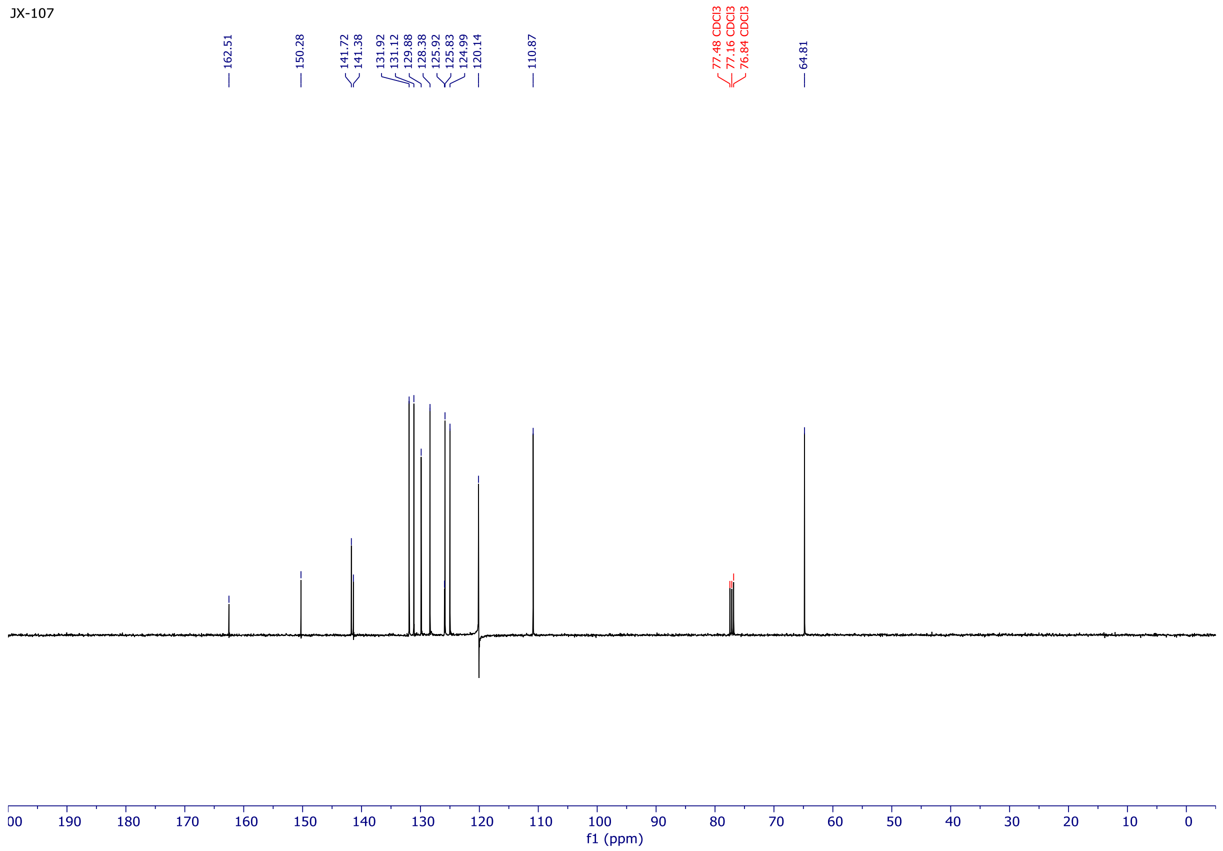

JX-107
162.51
150.28
141.72
141.38
131.92
131.12
129.88
128.38
125.92
125.83
124.99
120.14
110.87
77.48 CDCl3
77.16 CDCl3
76.84 CDCl3
64.81
f1 (ppm)

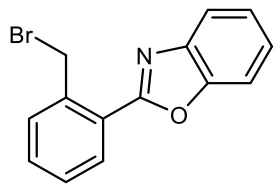

JX-108

2-(2-(Bromomethyl)phenyl)benzo[d]oxazole (JX-108): To a suspension of **JX-107** (78 mg, 0.35 mmol, 1.0 equiv) in dry CH_2Cl_2 (3.5 mL) were added CBr_4 (172 mg, 0.519 mmol, 1.5 equiv) and PPh_3 (136 mg, 0.519 mmol, 1.5 equiv) at 0 °C under argon. After 1 h, the reaction was quenched by adding H_2O. The mixture was extracted with CH_2Cl_2 (3x25 mL) and the combined organi extracts were dried with $MgSO_4$, filtered, and concentrated under reduced pressure. The crude product was purified by flash column chromatography (hexane/EtOAc 9:1) to yield **JX-108** (82 mg, 0.29 mmol, 82%) as a beige white solid.

TLC (SiO_2; hexane/EtOAc 9:1, UV, $KMnO_4$ stain): R_f = 0.35.

mp: 93.6-94.4 °C.

^{1}H-NMR (400 MHz, $CDCl_3$): δ (ppm) = 8.23 (dd, *J* = 7.4, 1.9 Hz, 1H), 7.90 – 7.79 (m, 1H), 7.66 – 7.55 (m, 2H), 7.54 – 7.43 (m, 2H), 7.43 – 7.35 (m, 2H), 5.34 (s, 2H).

^{13}C-NMR (101 MHz, $CDCl_3$): δ (ppm) = 161.66, 150.28, 142.23, 138.09, 132.18, 131.47, 130.37, 128.90, 125.75, 125.59, 124.73, 120.56, 110.71, 32.35.

IR (thin film): υ (cm^{-1}) = 3059, 1613, 1550, 1491, 1474, 1453, 1443, 1342, 1311, 1285, 1268, 1242, 1223, 1208, 1195, 1112, 1031, 1003, 926, 894, 880, 831, 795, 777, 760, 743, 702, 626, 603, 580, 531, 504.

HR-MS (ESI): Calcd for $C_{14}H_{11}BrNO$ $[M+H]^+$, 288.0019 *m/z*; found, 288.0019 *m/z*.

JX-108

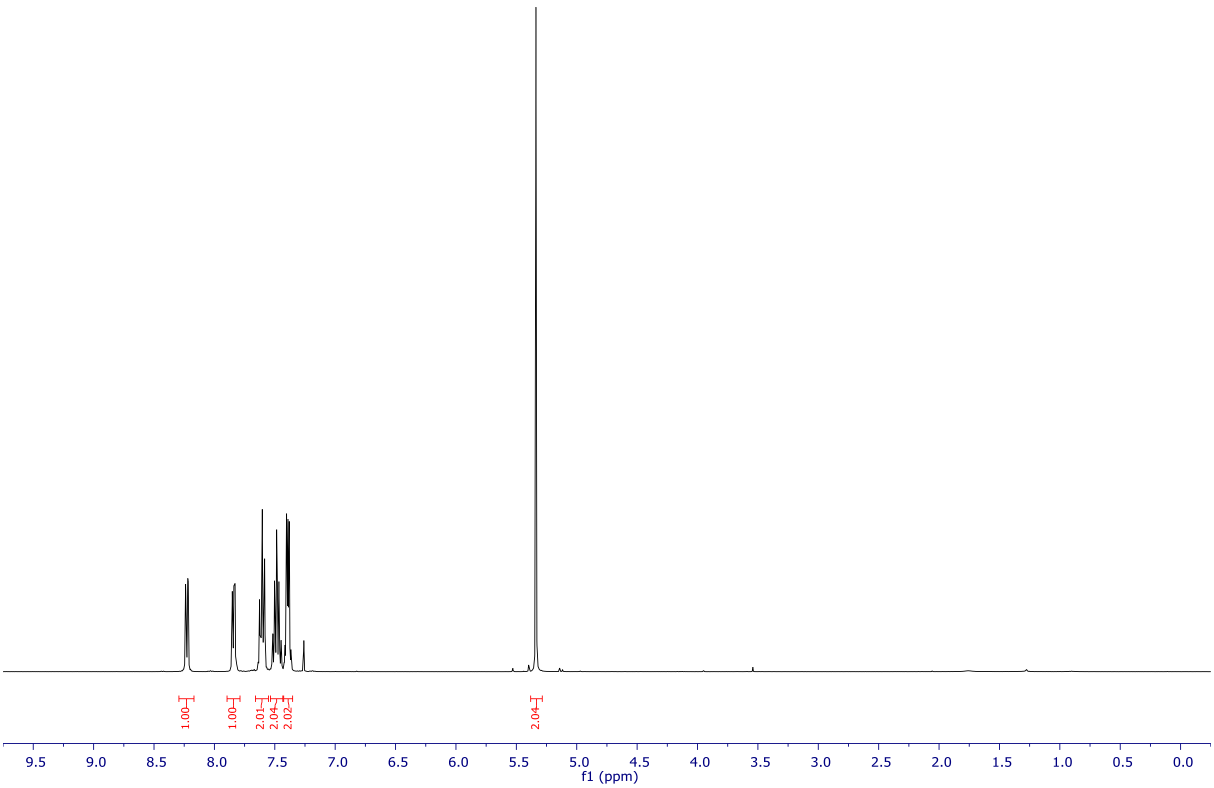
1.00
1.00
2.01
2.04
2.02
2.04
9.5 9.0 8.5 8.0 7.5 7.0 6.5 6.0 5.5 5.0 4.5 4.0 3.5 3.0 2.5 2.0 1.5 1.0 0.5 0.0
f1 (ppm)

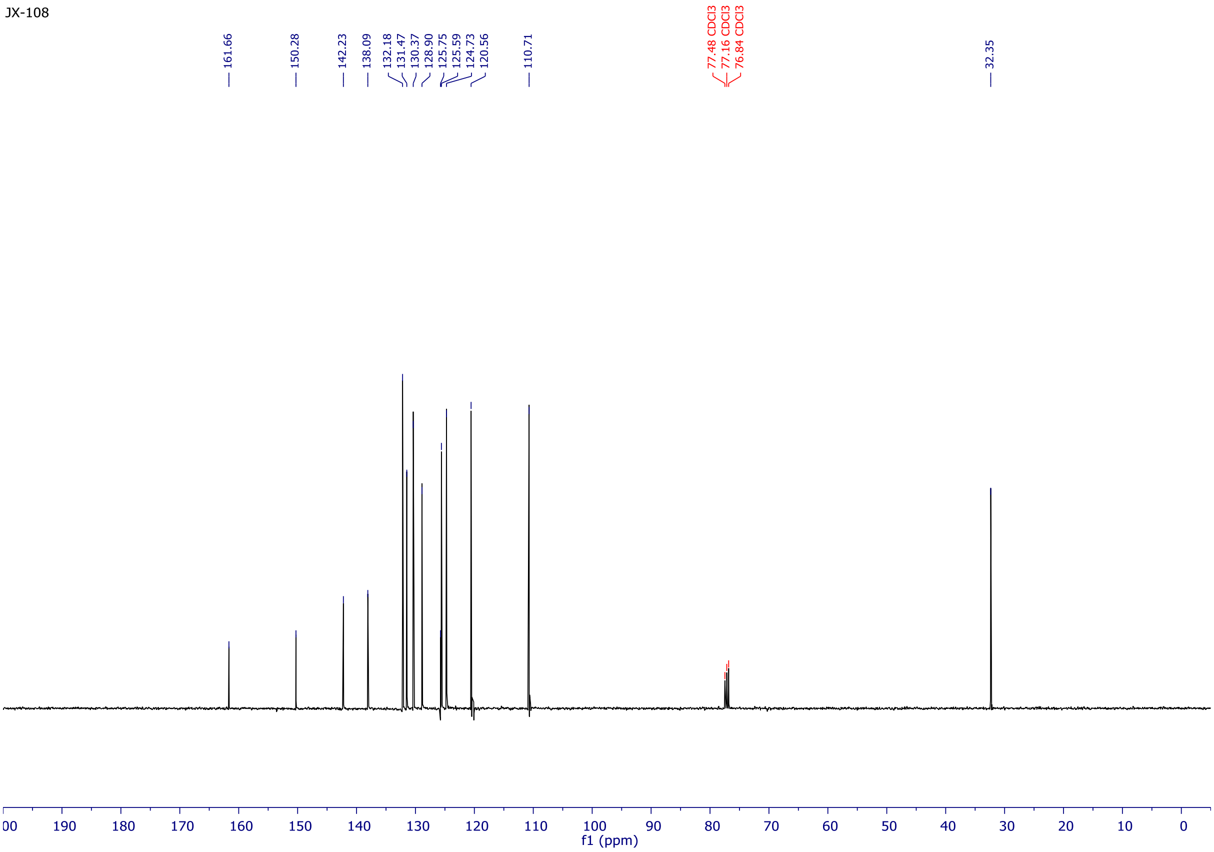

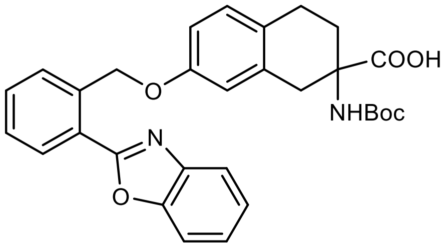

JX-110

7-((2-(Benzo[d]oxazol-2-yl)benzyl)oxy)-2-((tert-butoxycarbonyl)amino)-1,2,3,4-tetrahydronaphthalene-2-carboxylic acid (JX-110): To a stirred solution of **KH-142** (15 mg, 0.048 mmol, 1.0 equiv) in dry THF (0.5 mL) was added NaH (1 mg, 0.06 mmol, 1.2 equiv) , followed by **JX-108** (17 mg, 0.057 mmol, 1.2 equiv) at rt under argon. After 16 h, additional NaH (1 mg, 0.06 mmol, 1.2 equiv) was added and the reaction mixture was left stirring for 8 h. The reaction mixture was diluted with H_2O (1.5 mL) and after 17 h heated to 50 °C with additional NaOH (spatula tip). After 3 h at 50 °C, the reaction mixture was extracted with EtOAc (3 × 25 mL) and the combined organic phases were washed with brine (3 × 10 mL), dried with $MgSO_4$, filtered, and concentrated under

reduced pressure. The crude product was purified by flash column chromatography (CH_2Cl_2/MeOH 93:7) to yield **JX-110** (2 mg, 7%) as a white solid together with unreacted methyl ester.

The unreacted methyl ester and $LiOH \cdot H_2O$ (20 mg, 0.48 mmol, 10.0 equiv) were dissolved in THF/H_2O (1:1, 1.0 mL) and stirred at rt. After 6 h, the reaction mixture was heated to 60 °C. After 17 h, additional $LiOH \cdot H_2O$ (20 mg, 0.48 mmol, 10.0 equiv) was added. After 74 h, the reaction mixture was extracted with EtOAc (3 × 25 mL) and the combined organic phases were washed with brine (3 × 10 mL), dried with $MgSO_4$, filtered, and concentrated under reduced pressure. The crude product was purified by flash column chromatography (CH_2Cl_2/MeOH 93:7 → 95:5) to yield **JX-110** (21 mg, 85%).

TLC (SiO_2; CH_2Cl_2/MeOH 95:5, UV, $KMnO_4$ stain): R_f = 0.18.

mp: 183.9-186.5 °C.

^{1}H-NMR (400 MHz, $(CD_3)_2SO$): δ (ppm) = 12.36 (brs, 1H), 8.23 (dd, *J* = 7.8, 1.5 Hz, 1H), 7.89 – 7.79 (m, 1H), 7.80 – 7.72 (m, 2H), 7.65 (ddd, *J* = 7.5, 1.5 Hz, 1H), 7.57 (ddd, *J* = 7.6, 1.4 Hz, 1H), 7.44 (pd, *J* = 7.4, 1.4 Hz, 2H), 7.03 (s, 1H), 6.96 (d, *J* = 8.5 Hz, 1H), 6.72 (dd, *J* = 8.3, 2.7 Hz, 1H), 6.68 (d, *J* = 2.7 Hz, 1H), 5.66 (s, 2H), 3.11 (d, *J* = 16.7 Hz, 1H), 2.96 (d, *J* = 16.9 Hz, 1H), 2.78 – 2.65 (m, 1H), 2.60 (ddd, *J* = 16.6, 5.3 Hz, 1H), 2.30 – 2.13 (m, 1H), 1.93 – 1.79 (m, 1H), 1.34 (s, 9H).

^{13}C-NMR (101 MHz, $(CD_3)_2SO$): δ (ppm) = 175.71, 161.59, 156.24, 155.06, 149.72, 141.45, 137.71, 135.40, 131.58, 129.55, 129.13, 128.15, 128.06, 127.48, 125.73, 124.82, 124.17, 120.04, 115.11, 112.58, 110.90, 77.86, 67.82, 56.93, 36.62, 28.99, 28.18, 24.15.

IR (thin film): υ (cm^{-1}) = 3426, 3325, 3061, 2976, 2926, 1712, 1613, 1580, 1551, 1501, 1454, 1392, 1367, 1243, 1162, 1127, 1109, 1065, 1028, 924, 894, 853, 805, 778, 760, 743, 704.

HR-MS (ESI): Calcd for $C_{30}H_{30}N_2NaO_6$ $[M+Na]^+$, 537.1996 *m/z*; found, 537.1999 *m/z*.

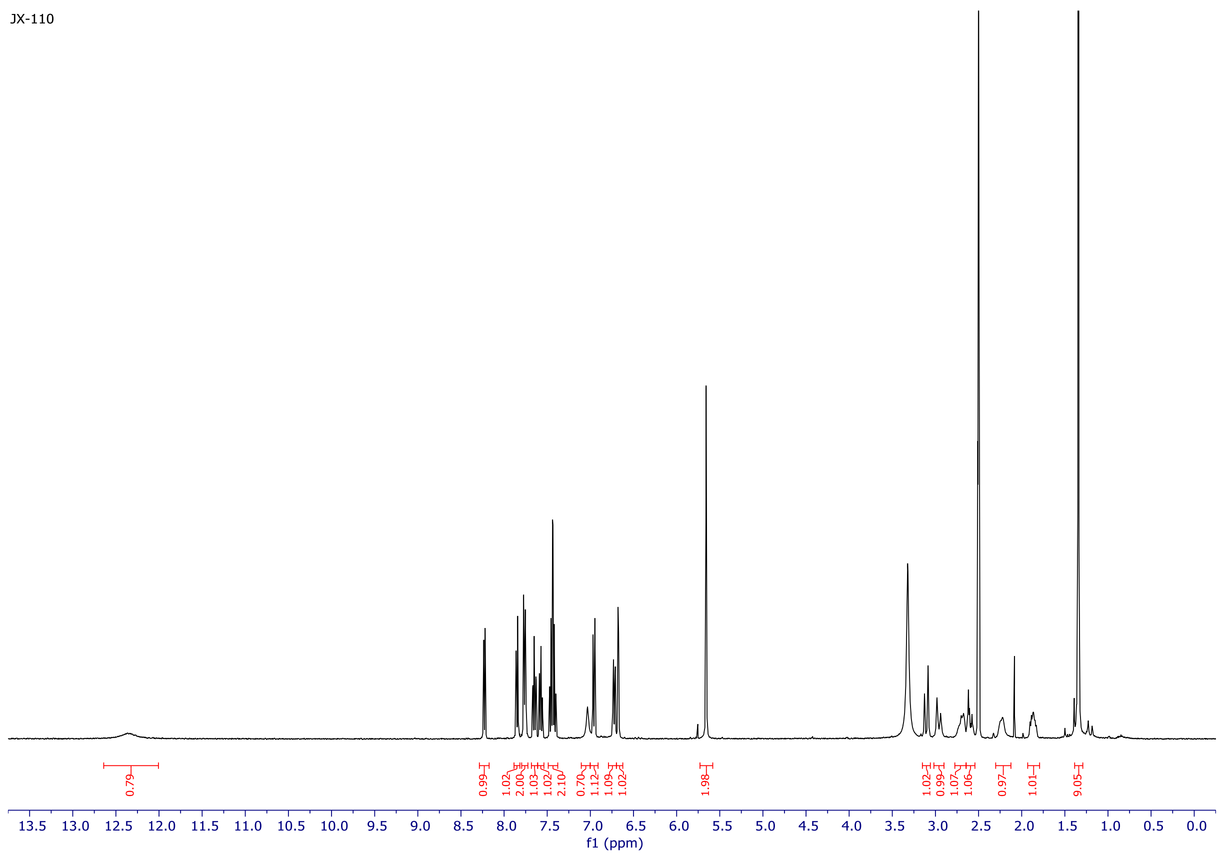
JX-110
0.79
0.99
1.02
2.00
1.03
1.02
2.10
0.70
1.12
1.09
1.02
1.98
1.02
0.99
1.07
1.06
0.97
1.01
9.05
13.5 13.0 12.5 12.0 11.5 11.0 10.5 10.0 9.5 9.0 8.5 8.0 7.5 7.0 6.5 6.0 5.5 5.0 4.5 4.0 3.5 3.0 2.5 2.0 1.5 1.0 0.5 0.0
f1 (ppm)

JX-110

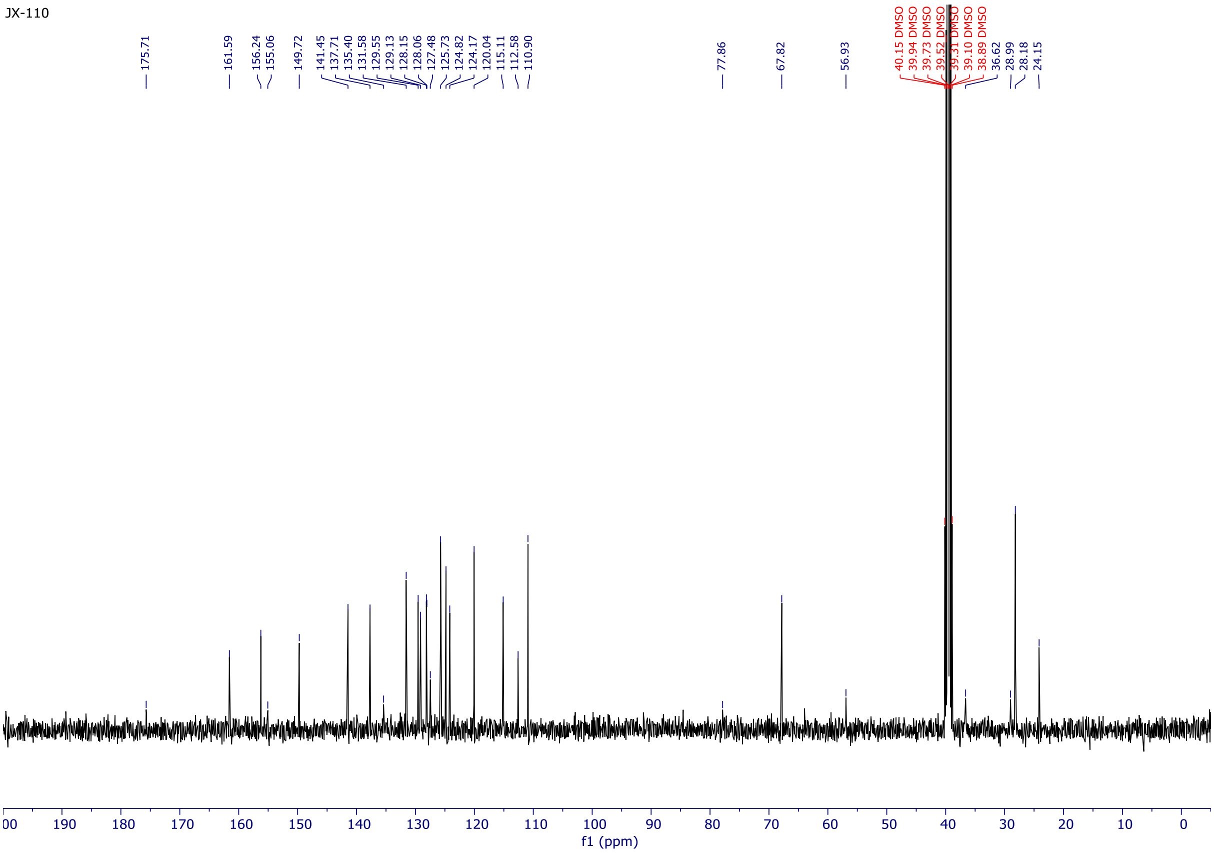
175.71
161.59
156.24
155.06
149.72
141.45
137.71
135.40
131.58
129.55
129.13
128.15
128.06
127.48
125.73
124.82
124.17
120.04
115.11
112.58
110.90
77.86
67.82
56.93
40.15 DMSO
39.94 DMSO
39.73 DMSO
39.52 DMSO
39.31 DMSO
39.10 DMSO
38.89 DMSO
36.62
28.99
28.18
24.15
f1 (ppm)

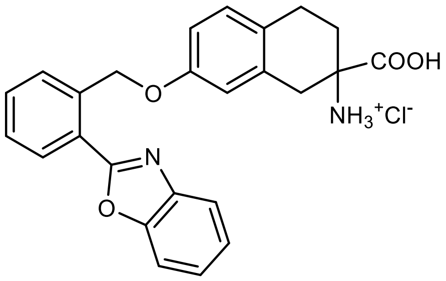

JX-119

7-((2-(Benzo[d]oxazol-2-yl)benzyl)oxy)-2-carboxy-1,2,3,4-tetrahydronaphthalen-2-aminium chloride (JX-119): JX-110 (6 mg, 0.01 mmol, 1.0 equiv) was dissolved in HCl (3 M in CPME, 1.0 mL) and stirred at rt under argon. After 16 h a precipitate was formed, which was collected by filtration using a Pasteur pipette equipped with cotton and a layer of celite. The residue was thoroughly washed with CPME and then flushed down using MeOH. The solvent was evaporated under reduced pressure to yield **JX-119** (4 mg, 0.01 mmol, 78%) as a beige solid.

TLC (SiO_2; $CHCl_3$/MeOH/H_2O 70:30:5), UV, $KMnO_4$ stain): R_f = 0.41.

mp: 234-238 °C (burned/decomposition).

^{1}H-NMR (400 MHz, CD_3OD): δ (ppm) = 8.24 (dd, *J* = 7.7, 1.5 Hz, 1H), 7.79 (dd, *J* = 7.7, 1.0 Hz, 1H), 7.76 (ddd, *J* = 5.9, 2.3, 0.5 Hz, 1H), 7.65 – 7.61 (m, 1H), 7.60 (ddd, *J* = 7.9, 7.5, 1.5 Hz, 1H), 7.54 (ddd, *J* = 7.6, 7.4, 1.5 Hz, 1H), 7.46 – 7.37 (m, 2H), 7.10 (d, *J* = 8.5 Hz, 1H), 6.86 (dd, *J* = 8.5, 2.7 Hz, 1H), 6.75 (d, *J* = 2.6 Hz, 1H), 5.69 (s, 2H), 3.44 (d, *J* = 17.3 Hz, 1H), 3.02 – 2.91 (m, 1H), 2.94 (d, *J* = 16.6 Hz, 1H), 2.82 (ddd, *J* = 17.0, 10.1, 5.9 Hz, 1H), 2.38 (ddd, *J* = 14.0, 10.1, 6.5 Hz, 1H), 2.14 (dddd, *J* = 13.8, 6.1, 4.9, 1.6 Hz, 1H).

^{13}C-NMR (101 MHz, CD_3OD): δ (ppm) = 173.58, 163.74, 158.90, 151.64, 143.02, 139.16, 132.88, 132.56, 131.17, 130.87, 129.49, 129.15, 127.33, 126.84, 126.02, 125.92, 120.96, 116.02, 115.54, 111.71, 69.69, 59.49, 36.40, 30.37, 24.54.

IR (solid): υ (cm^{-1}) = 3411, 2925, 1728, 1614, 1582, 1548, 1498, 1455, 1379, 1315, 1273, 1241, 1160, 1130, 1056, 1030, 920, 874, 849, 803, 759, 751, 721, 537, 484, 452, 415.

HR-MS (ESI): Calcd for $C_{25}H_{23}N_2O_4$ $[M+H]^+$, 415.1652 *m/z*; found, 415.1656 *m/z*. Calcd for $C_{25}H_{21}N_2O_4$ $[M-H]^-$, 413.1507 *m/z*; found, 413.1514 *m/z*.

JX-119

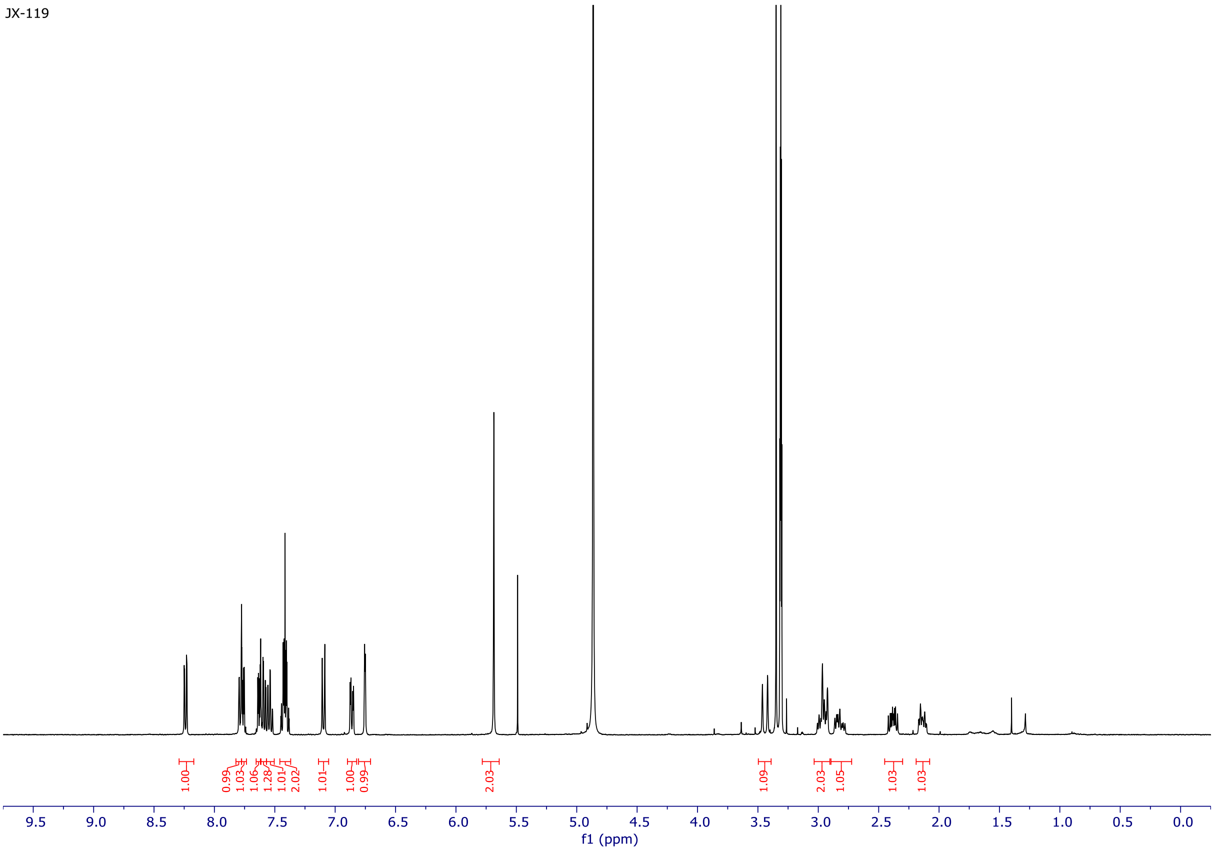

1.00
0.99
1.03
1.06
1.28
1.01
2.02
1.01
1.00
0.99
2.03
1.09
2.03
1.05
1.03
1.03
9.5 9.0 8.5 8.0 7.5 7.0 6.5 6.0 5.5 5.0 4.5 4.0 3.5 3.0 2.5 2.0 1.5 1.0 0.5 0.0
f1 (ppm)

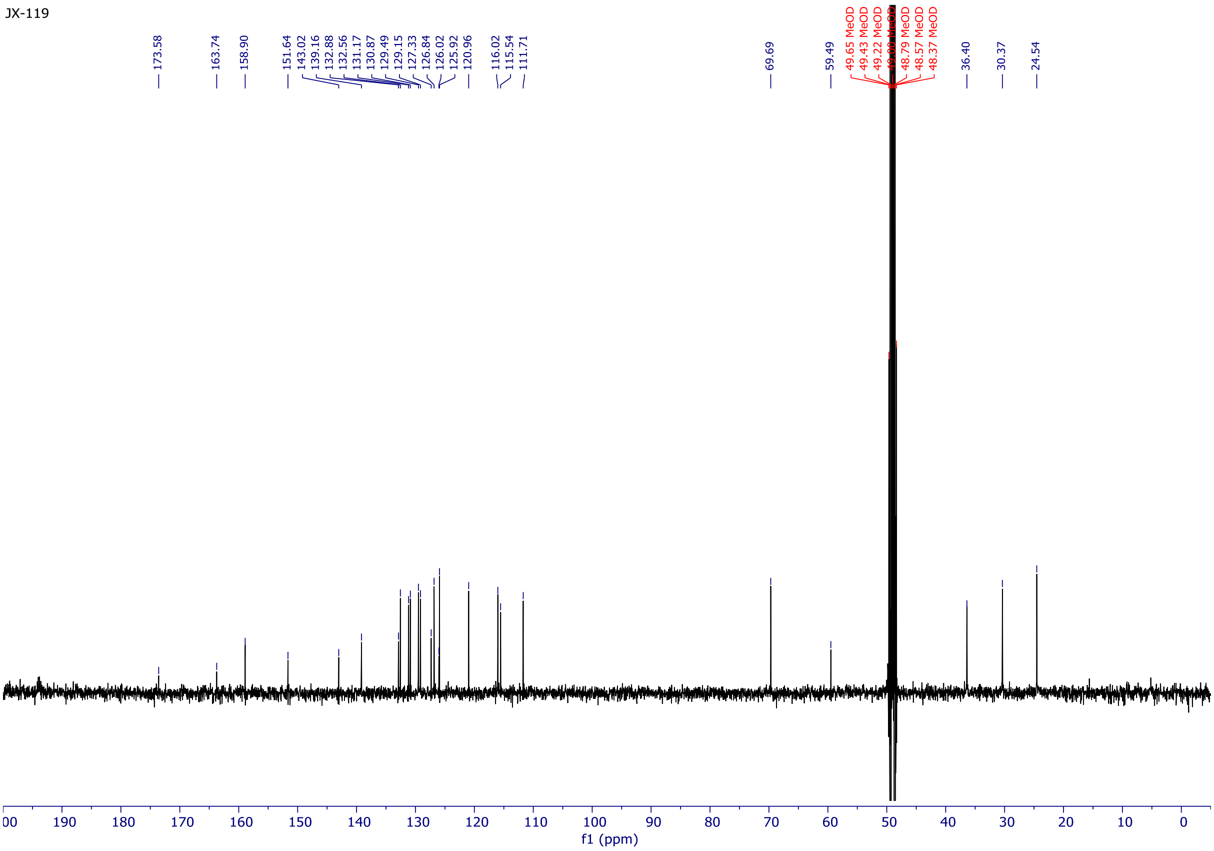

JX-119
173.58
163.74
158.90
151.64
143.02
139.16
132.88
132.56
131.17
130.87
129.49
129.15
127.33
126.84
126.02
125.92
120.96
116.02
115.54
111.71
69.69
59.49
49.65 MeOD
49.43 MeOD
49.22 MeOD
48.79 MeOD
48.57 MeOD
48.37 MeOD
36.40
30.37
24.54
f1 (ppm)
